# A cell surface-exposed protein complex with an essential virulence function in *Ustilago maydis*

**DOI:** 10.1038/s41564-021-00896-x

**Published:** 2021-05-03

**Authors:** Nicole Ludwig, Stefanie Reissmann, Kerstin Schipper, Carla Gonzalez, Daniela Assmann, Timo Glatter, Marino Moretti, Lay-Sun Ma, Karl-Heinz Rexer, Karen Snetselaar, Regine Kahmann

**Affiliations:** 1 Department of Organismic Interactions, Max Planck Institute for Terrestrial Microbiology, Marburg, Germany; 2 Mass Spectrometry and Proteomics, Max Planck Institute for Terrestrial Microbiology, Marburg, Germany; 3 Department of Evolutionary Ecology of Plants, Philipps-Universität Marburg, Marburg, Germany; 4 Department of Biology, Saint Joseph’s University, Philadelphia, PA USA; 5 Present Address: Institut für Mikrobiologie, Heinrich-Heine-Universität Düsseldorf, Düsseldorf, Germany; 6 Present Address: Institute of Plant and Microbial Biology, Academia Sinica, Taipei, Taiwan

**Keywords:** Fungal pathogenesis, Effectors in plant pathology

## Abstract

Plant pathogenic fungi colonizing living plant tissue secrete a cocktail of effector proteins to suppress plant immunity and reprogramme host cells. Although many of these effectors function inside host cells, delivery systems used by pathogenic bacteria to translocate effectors into host cells have not been detected in fungi. Here, we show that five unrelated effectors and two membrane proteins from *Ustilago maydis*, a biotrophic fungus causing smut disease in corn, form a stable protein complex. All seven genes appear co-regulated and are only expressed during colonization. Single mutants arrest in the epidermal layer, fail to suppress host defence responses and fail to induce non-host resistance, two reactions that likely depend on translocated effectors. The complex is anchored in the fungal membrane, protrudes into host cells and likely contacts channel-forming plant plasma membrane proteins. Constitutive expression of all seven complex members resulted in a surface-exposed form in cultured *U. maydis* cells. As orthologues of the complex-forming proteins are conserved in smut fungi, the complex may become an interesting fungicide target.

## Abstract (web summary)

This study reports that five effectors and two transmembrane proteins from the plant pathogenic fungus *Ustilago maydis* form a stable cell surface-exposed protein complex required for virulence.

## Main

The smut fungus *U. maydis* is an important pathogen of corn^1^. Primary disease symptoms are plant tumours in which fungal hyphae differentiate into spores. *U. maydis* is a biotrophic pathogen requiring living plant tissue for proliferation. In its dikaryotic form, *U. maydis* develops appressoria that penetrate the plant cuticle and cell wall. Invading hyphae become encased by the plant plasma membrane, resulting in an extended interaction zone^2^. At this stage, plant responses elicited by fungal MAMPs (microbe-associated molecular patterns) are actively suppressed by a cocktail of mostly novel secreted effectors. A large number of these effectors are expressed during establishment of the biotrophic stage^3^, and in this group are all six *U. maydis* virulence-promoting effectors functionally studied to date^4–11^. These effectors all interact with specific plant proteins and modulate their function either in the interface between fungus and surrounding plant plasma membrane or after translocation into invaded host cells^12–14^. The molecular mechanisms by which translocation is achieved are largely unknown^15,16^.

**Fig. 1:**
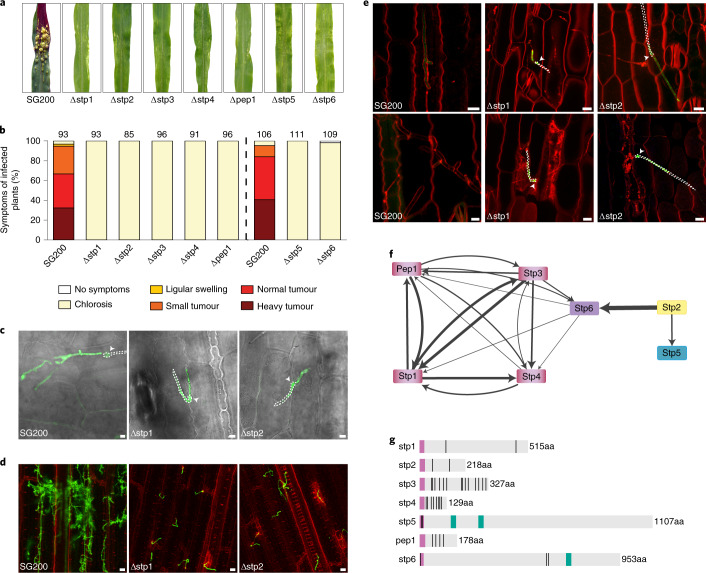
Seven *U. maydis* proteins essential for virulence form a protein complex. **a**,**b**, Seven-day-old maize seedlings were infected with SG200 and the indicated deletion strains. At 12 d.p.i., representative leaves were photographed (**a**) and disease symptoms were scored (**b**). The vertical dashed line separates two independent sets of experiments. Data represent the mean of *n* = 3 biologically independent experiments. Total numbers of infected plants are indicated above the respective columns. **c**, Maize epidermal cells 1 d.p.i. with SG200AN1, SG200AM1∆stp1 and SG200AN1∆stp2 all expressing cytosolic GFP upon plant penetration. The GFP (green) and bright-field (grey) channels are merged. Hyphae on the plant surface are traced in white; untraced hyphae are intracellular. Arrowheads indicate appressoria. Shown are maximum projections of confocal *z*-stacks. Scale bars, 5 μm. **d**, Maize leaves 3 d.p.i. with SG200 and the indicated deletion strains stained with WGA-AF488 (fungal cell wall, green) and propidium iodide (plant cell wall, red). Shown are AF488 (green) and propidium iodide (red) channel overlays of confocal *z*-stack maximum projections. Scale bars, 25 μm. **e**, Maize epidermal cells 1 d.p.i. (upper panel) and 2 d.p.i. (lower panel) with SG200AN1, SG200AM1∆stp1 and SG200AN1∆stp2 all expressing cytosolic GFP upon plant penetration. Plasma membranes were stained with FM4-46 (red). Shown are GFP (green) and FM4-46 (red) channel overlays of confocal *z*-stack maximum projections. Hyphae on the plant surface are traced in white; intracellular hyphae are untraced. Arrowheads indicate appressoria. Scale bars, 10 μm. **f**, Effector protein complex interaction network resulting from co-IP/MS experiments using Stp1, Stp2, Stp3, Stp4, Pep1 and Stp6 as bait proteins across several replicated experiments. Line widths illustrate the average number of spectral counts across experiments. **g**, Domain arrangement of the Stp proteins and calculated molecular weight without signal peptide. Violet, signal peptide; black vertical lines, cysteine residues; green, transmembrane domains predicted based on sequence conservation to membrane domains containing orthologues from other smut fungi (Supplementary Figs. 1–7).

**Extended Data Fig. 1:**
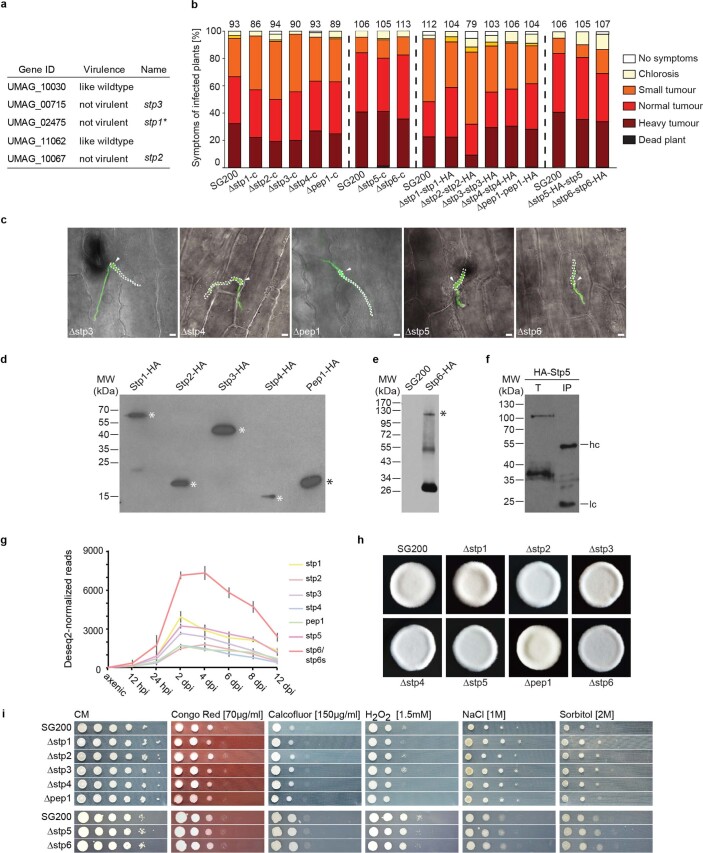
Complementation of the *stp* phenotype with wild type and HA-tagged alleles and characterization of *stp* deletion strains. **a**, List of putative effector genes deleted at the onset of this study. The observed virulence phenotype is indicated. * indicates that *stp1* was originally deleted as part of the virulence gene cluster 5b^18^ and subsequently shown to be responsible for the loss of virulence^19^. **b**, Seven-day-old maize seedlings were infected with the indicated *U. maydis* deletion strains, the respective single copy complemented strains (c), as well as deletion strains complemented with the respective single copy HA fusion protein. 12 d.p.i. disease symptoms were scored. The dashed lines separate sets of independent experiments. All data represent mean of n = 3 biologically independent experiments. Total numbers of infected plants are indicated above the respective columns. **c**, Maize epidermal cells 1 day post infection with the indicated *stp* deletion strains in the SG200AN1 or SG200AM1 background. Shown are confocal z-stack maximum projections of GFP (green) and bright-field (grey) channel overlays. Fluorescent hyphae on the plant surface are traced in white, untraced hyphae are intracellular. Arrows indicate appressoria. Scale bars, 5 μm. **d-e**, Anti-HA western blot of HA-specific immunoprecipitations from total maize leaf extracts 3 d.p.i. with the indicated *U. maydis* strains. **f**, Anti-HA western blot of the total extract (T) and eluate after HA-specific immunoprecipitation (IP) obtained from leaves 3 d.p.i. with the strain SG200Δstp5 HA-stp5. The HA-Stp5 protein, indicated by an asterisk, was only detectable after denaturing SDS-PAGE in total extracts (T) but the HA-tag was not accessible to IP under native conditions (IP). The heavy and light chain (hc, lc) of the HA-antibody used for IP appear here and not in (**d**) and (**e**) because in comparison to (**d**) and (**e**) the eluate from 10 times more beads was loaded. **g**, Relative expression levels of *stp* genes during biotrophic development of *U. maydis* were revealed by RNA sequencing analysis of RNA samples collected from FB1 × FB2 infected maize plants (dataset available via ref. ^3^ and GEO accession number GSE103876). The vertical axis indicates normalized counts from DESeq2 analysis. The horizontal axis indicates the infection stages: 0.5, 1, 2, 4, 6, 8 and 12 d.p.i. Data represent mean ± s.d. of n = 3 biological replicates. Colour codes for the different genes are indicated on the right. **h**, Filamentous growth of the spotted indicated strains was visible on PD-charcoal plates grown for 48 h at room temperature. **i**, Stress sensitivity of *stp* deletion strains. Serial 10-fold dilutions of SG200 and the indicated deletion strains were spotted on complete medium supplemented with 2% (w/v) glucose in the absence of stressors (CM) or in the presence of the indicated stressors. The plates were incubated for 48 h at 28 °C. Source data

To investigate the establishment of biotrophy in more detail, we initiated a systematic deletion analysis of predicted novel effector genes from the biotrophy-associated group and selected those that are already highly induced during plant penetration^17^. Among the top five genes (Fig. 1a,b and Extended Data Fig. 1a), we identified two putative effector genes, *stp2* (*UMAG_10067*) and *stp3* (*UMAG_00715*), where single gene deletions in the solopathogenic strain SG200 (ref. ^18^) abolished virulence completely (Fig. 1a,b). In addition, in this group was *stp1* (*UMAG_02475*), a gene previously shown to abolish virulence when deleted alone^18,19^. In all cases, the loss of virulence could be complemented by introducing a single copy of the respective gene with or without a haemagglutinin (HA) affinity tag (Extended Data Fig. 1b). To study the course of plant colonization by the *stp* mutants, the mutations were introduced into SG200 strains expressing a cytosolic green fluorescent protein (GFP) marker that is induced in appressoria and stays active during colonization^17,20^. One day after infection with SG200, the appressorial marker was expressed and branching hyphae could already be detected in mesophyll tissue (Fig. 1c). At the same time point, *stp1*, *stp2* and *stp3* mutants had penetrated via appressoria, but branching did not occur and hyphae arrested in the epidermal layer (hence the name ‘stop after penetration’ or *stp*; Fig. 1c,d and Extended Data Fig. 1c). Staining with FM4-64 revealed integrity of the plant plasma membrane surrounding biotrophic SG200 hyphae (Fig. 1e). Upon mutant infection, the plant plasma membrane initially invaginated and was intact; however, at time points later than 2 days post infection (d.p.i.), the FM4-64 stain in infected cells accumulated in vesicular structures and the plasma membrane appeared disrupted (Fig. 1e). These are typical signs of programmed plant cell death^21–23^. To obtain clues about the processes affected by the Stp proteins, we performed co-immunoprecipitations (co-IP) followed by mass spectrometry (MS) from extracts of maize leaves 3 d.p.i. with *U. maydis* strains expressing genes for either Stp1-HA, Stp2-HA or Stp3-HA under control of their native promoters. All HA-tagged proteins were detected after IP and subsequent western blot analysis (Extended Data Fig. 1d). However, instead of the expected plant interaction partners, we extracted a fungal protein complex consisting of Stp1, Stp3, Pep1, the putative effector UMAG_12197 (termed Stp4) and UMAG_01695 (termed Stp6) (Fig. 1f and Supplementary Data 1). *stp6* has previously been identified as a Biz1-regulated gene with a critical virulence function (J. Kämper and M. Vraneš, personal communication), and Pep1 has been shown to inhibit the apoplastic maize peroxidase POX12 (ref. ^6^). Stp2-HA also co-immunoprecipitated with Stp6 and, in addition, with the as yet uncharacterized *U. maydis* protein UMAG_04342, termed Stp5. Stp6 and Stp5 orthologues exist in all sequenced smut fungi, and most are predicted to harbour transmembrane domains (Fig. 1g and Supplementary Fig. 6,7). Deletion mutants of either *stp4*, *pep1* (ref. ^4^), *stp6* or *stp5* were unable to cause disease (Fig. 1a,b) and the phenotype could be fully complemented by respective wild-type or HA-tagged genes (Extended Data Fig. 1b). Microscopic analyses revealed a proliferation arrest after penetration (Extended Data Fig. 1c), very similar to the phenotype of *stp1, stp2* and *stp3* mutants (Fig. 1c and Extended Data Fig. 1c). While these experiments were ongoing, *stp4* was published as the essential effector *cce1* (ref. ^24^). Reciprocal co-IP/MS experiments from extracts after infection with strains expressing Stp4-HA, Pep1-HA and Stp6-HA confirmed the presence of a heptameric complex. Consistent co-purification of Stp1, Stp3, Stp4, Pep1 and Stp6 as well as Stp2, Stp5 and Stp6 suggests the existence of two sub-complexes with Stp6 present in both protein assemblies (Fig. 1f and Supplementary Data 1). HA-Stp5 could not be enriched in an anti-HA IP, presumably because of inaccessibility of the HA tag, but could be visualized in total extracts (Extended Data Fig. 1f). Except for Stp6-HA, where prominent smaller-than-full-length products likely resulting from processing were detected, the HA fusion proteins had the expected sizes (Fig. 1g and Extended Data Fig. 1d,e). The seven complex members differ in size, are unrelated in amino acid sequence (Fig. 1g) and reside in different locations in the genome but appeared co-regulated, showing no expression in axenic culture, strong induction during colonization and an expression peak at 2 d.p.i. (Extended Data Fig. 1g). All complex members were detected in all 11 analysed smut genomes (Supplementary Figs. 1–7). None of the mutants were affected in filamentation or sensitivity to various stresses (Extended Data Fig. 1h,i). This illustrates that the heptameric complex identified here, which we have designated the ‘Stp complex’, is specifically required during plant colonization.

**Extended Data Fig. 2:**
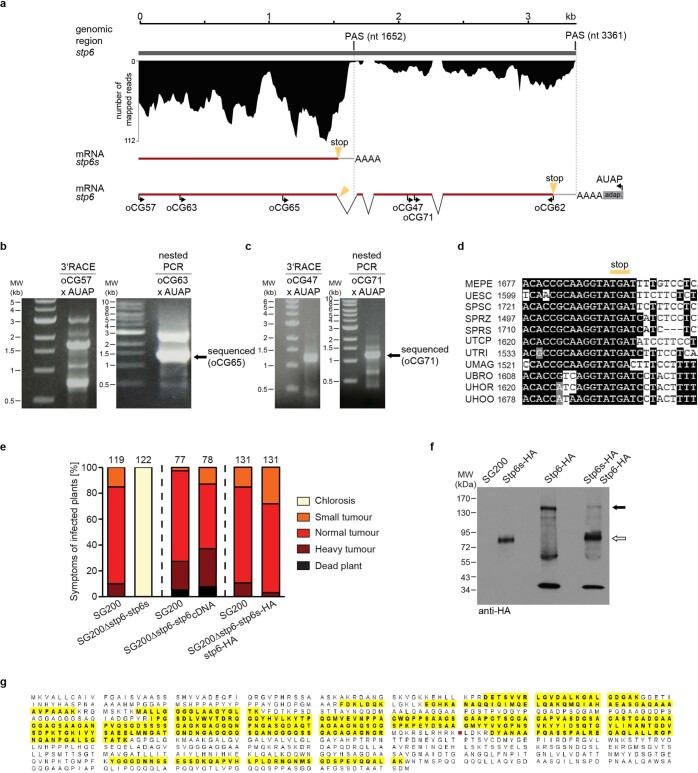
Differential polyadenylation of the *stp6* mRNA produces in addition to Stp6 a short Stp6s protein lacking the putative transmembrane domain. **a**, Scheme of the *stp6* genomic region. The two polyadenylation sites (PAS) are indicated. Displayed in black are mapped RNA sequencing reads generated from FB1×FB2 infected maize plants 24 hours post infection (dataset available via ref. ^3^ and GEO accession number GSE103876). The *stp6s* and *stp6* mRNAs derived from the *stp6* genomic region are displayed as red lines. Yellow arrows indicate the *stp6s* and *stp6* stop codons. Note that the *stp6s* stop codon is located in the first intron of *stp6*. Binding sites of oligonucleotides used in 3’RACE experiments and nested PCRs are indicated. The differences in abundance result from efficient polyadenylation after nucleotide position 1652 downstream of a stop codon conserved in all orthologues (leading to a 511 amino acid Stp6s (Stp6small) protein that lacks a transmembrane domain. Occasionally the first polyA signal is skipped and polyadenylation occurs after nucleotide 3661. Splicing of this mRNA eliminates the stop codon of *stp6s*, leading to a 953 amino acid Stp6 protein predicted to have a single transmembrane domain (Supplementary Fig. 7). **b-c**, DNA gels of products from 3’RACE PCR (left) and the corresponding nested PCR (right) with the indicated primer pairs. AUAP : PolyA adaptor primer provided by the 3’RACE kit. Black arrows indicate PolyA containing fragments of *stp6s* (**b**) and *stp6* (**c**) that were sequenced. **d**, The stop codon of *stp6s* is conserved in orthologues genes. Nucleotide sequence alignment of the region containing the stop codon of *stp6s* from 11 sequenced smut fungi. MEPE: *Melanopsichium pennsylvanicum*; UESC: *Ustilago esculenta*; SPSC: *Sporisorium scitamineum*; SPRZ: *Sporisorium reilianum* f. sp. *zeae*; SPRS: *Sporisorium reilianum* f. sp. *sorghi*; UTCP: *Ustilago trichophora*; UTRI: *Ustilago tritici*; UMAG: *U. maydis*; UBRO: *Ustilago bromivora*; UHOR: *Ustilago hordei* (host: barley); UHOO: *Ustilago hordei* (host: oats). **e**, *stp6* cDNA complements the *stp6* deletion phenotype while *stp6s* is unable to complement. Seven-day-old maize seedlings were infected with the *U. maydis stp6* deletion strain complemented with either *stp6s*, the *stp6* cDNA or the *stp6* genomic region with HA-tags inserted directly upstream of the *stp6s* stop codon and the *stp6* stop codon (stp6s-HA stp6-HA). 12 d.p.i. disease symptoms were scored. The dashed lines indicate separate experiments. Data represent mean of n = 3 biologically independent experiments. Total numbers of infected plants are indicated above the respective columns. **f**, Detection of Stp6s-HA and Stp6-HA after immunoprecipitation. Plants were infected with strains SG200Δstp6-stp6s-HA (a *stp6* deletion strain complemented with the *stp6* genomic region with the HA-tag inserted directly upstream of the Stp6s stop codon), SG200Δstp6-stp6-HA (carrying the HA-tag at the C-terminus of Stp6) as well as the SG200Δstp6-stp6s-HA-stp6-HA described in **e**. Extracts from leaves were generated at 3 d.p.i. and subjected to HA-specific immunoprecipitation. Shown is an anti-HA western blot. Lanes are labeled with the expected HA-tagged proteins expressed. The filled arrow indicates Stp6-HA, the open arrow indicates Stp6s-HA. **g**, Stp6 is expressed during colonization. Plants were infected with SG200Δstp2-stp2-HA. Extracts from leaves were prepared at 3 d.p.i., subjected to co-IP/MS. Shown is a screenshot from Scaffold (v4.6.2, Proteome Software) of the Stp6 amino acid sequence. Highlighted in yellow are Stp6 peptides identified. The red square marks the end of Stp6s. Peptides identified from the region downstream of Stp6s indicate that the full-length Stp6 protein is expressed and is part of the complex. Source data

**Extended Data Fig. 3:**
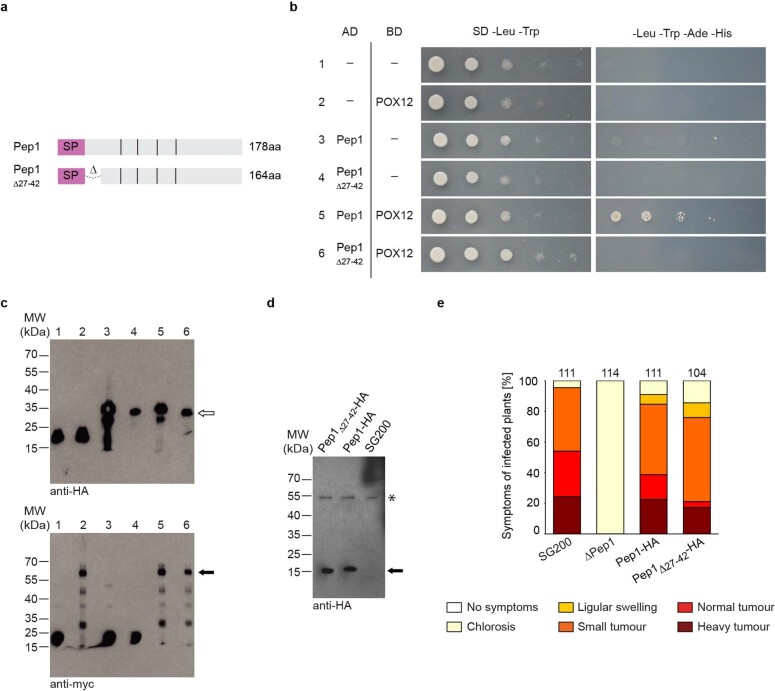
Pep1 is a dual function effector and its presence in the Stp complex is essential for virulence. **a**, Scheme of Pep1 and Pep1_Δ27-42_. Violet: signal peptide; black vertical lines: cysteine residues. **b**, In contrast to the full-length protein, Pep1_Δ27-42_ does not interact with maize POX12. Serial dilutions of AH109 yeast strains numbered 1 to 6, co-transformed with two plasmids expressing the indicated proteins without signal peptide as fusion to the GAL4 activation domain (AD) or GAL4 binding domain (BD) were grown on SD –Leu –Trp plates for growth control and SD –Leu –Trp –Ade –His plates to assess protein interaction. **c**, Expression of the fusion proteins in strains 1-6 from (b) was confirmed by HA-specific western blot for AD fusion proteins (top panel) and Myc-specific western blot for BD fusion proteins (lower panel). The open arrow labels AD-HA-Pep1, the closed arrow labels BD-myc-POX12. **d**, anti-HA western blot of the eluate after HA-specific immunoprecipitation from total extracts of leaves 3 d.p.i. with *U. maydis* strains expressing the indicated HA-fusion proteins. The arrow marks Pep1. The asterisk marks an unspecific signal. **e**, Seven-day-old maize seedlings were infected with the indicated *U. maydis* strains. Disease symptoms were scored at 12 d.p.i. using the disease scores depicted on the bottom. Data represent mean of n = 3 biologically independent experiments. Total numbers of infected plants are indicated above the respective columns. Source data

*stp6* has a complex gene structure encoding two mRNAs that are expressed at different levels^3^ and encode both a short N-terminal polypeptide (Stp6s) and a long version (Stp6), with Stp6 predicted to have a single transmembrane domain (Extended Data Fig. 2a–d). *stp6* cDNA expressed from the *stp6* promoter was able to complement the *stp6* deletion phenotype, while *stp6s* alone was not able to complement it (Extended Data Fig. 2e). Co-IP/MS analysis from tissue infected with a Stp2-HA expressing strain identified peptides covering the entire length of Stp6 (Extended Data Fig. 2g), confirming that Stp6 is a component of the complex. Finding that the effector Pep1 (ref. ^6^) was now present in the Stp complex raised the possibility that Pep1 might be a dual function effector that acts very early in the apoplast when the peroxidase POX12 is expressed^3^ and subsequently acts in the complex. To address this, we tried to separate the two functions by mutational analysis and found that a Pep1_Δ27–42_-HA protein lacking 15 amino acids downstream of the signal peptide was unable to interact with POX12 in a yeast two-hybrid assay (Extended Data Fig. 3a–c). The fact that this mutant protein was expressed to similar levels as Pep1-HA and complemented the *pep1* mutant phenotype (Extended Data Fig. 3d,e) must therefore reflect the function of Pep1 in the effector complex.

**Extended Data Fig. 4:**
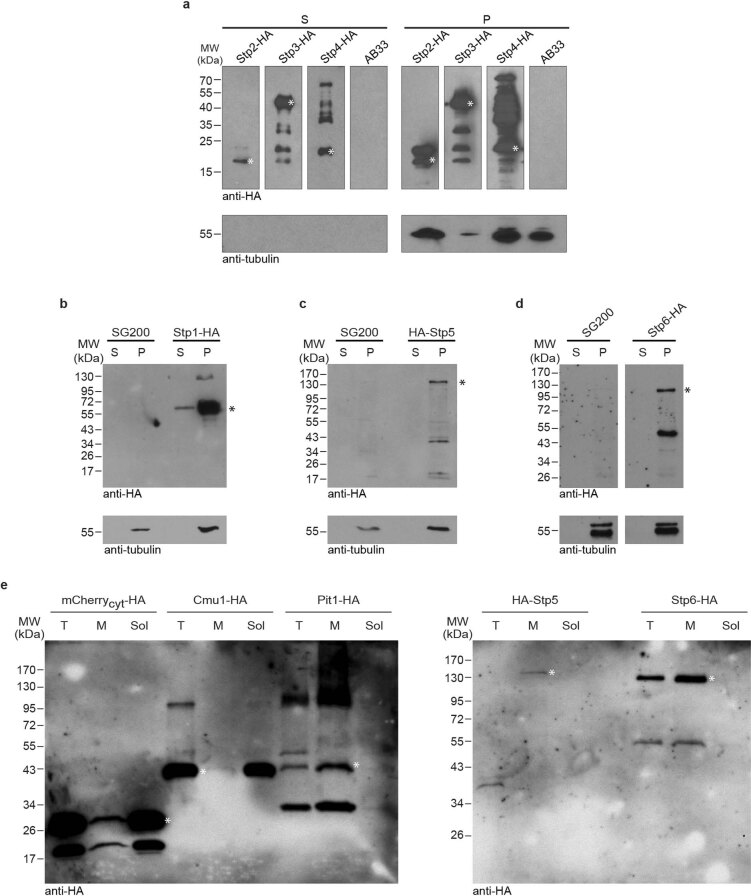
Stp1, Stp2, Stp3, Stp4 are secreted whereas Stp5 and Stp6 localize to the fungal plasma membrane upon constitutive expression. **a-d**, HA-specific western blot to reveal secretion of Stp proteins after constitutive expression from the *otef* promoter in AB33 (**a**), SG200Δkex2 (**b**) or SG200 (**c, d**). Supernatant fractions (S), as well as pellet fractions (P) were loaded. Cultures in (**d**) were grown in the presence of protease inhibitors. Stp1 contains several predicted Kex2 processing sites (Supplementary Fig. 1) and to detect Stp1-HA in culture supernatants it was necessary to express the protein in a strain lacking *kex2*. The tubulin specific western blot serves as a lysis control. Proteins of the expected size are marked with an asterisk. The molecular weight in kDa is indicated. **e**, HA-specific western blot revealing membrane association of HA-Stp5 and Stp6-HA. Total extract (T), plasma membrane fraction (M) and soluble fraction (Sol) from SG200-derived strains expressing the indicated fusion proteins were loaded. mCherry_cyt_ serves as control for a cytoplasmic protein, Cmu1-HA serves as control for a secreted protein^5^ and Pit1-HA serves as control for a fungal plasma membrane protein^57^. Full-length proteins are marked with an asterisk and molecular weight markers are indicated. HA-Stp6 co-migrates with an unspecific signal also detected in the pellet fraction of SG200 in (**d**). Source data

To test whether Stp complex members are secreted, we expressed all HA-tagged Stp proteins from a constitutive promoter. In the supernatants of their respective cultures, Stp1, Stp2, Stp3 and Stp4 proteins could be detected by western blot, while HA-Stp5 and Stp6-HA could not be detected (Extended Data Fig. 4a–d). Instead, HA-Stp5 and Stp6-HA localized to the fungal plasma membrane fraction (Extended Data Fig. 4e). These results strongly suggest that the Stp complex is anchored in the fungal membrane.

**Fig. 2:**
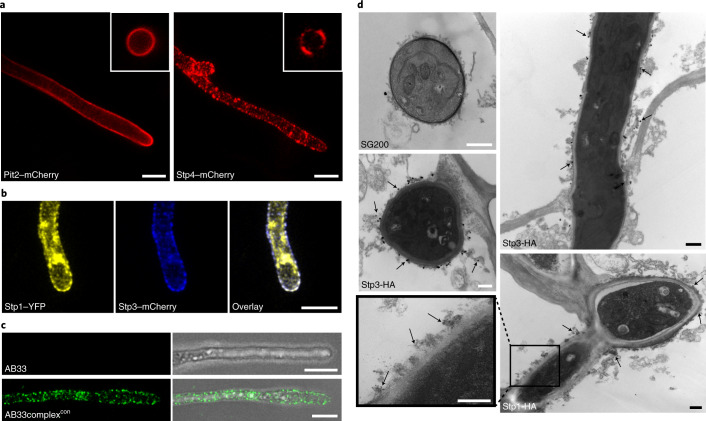
The Stp complex localizes to speckles on the surface of fungal hyphae. **a**, SG200 derived strains expressing the indicated fusion proteins growing in the epidermal layer at 2 d.p.i. mCherry signal, red. Images represent maximum projections of confocal *z*-stacks. Scale bars, 5 μm. Insets show cross-sections of fungal hyphae. **b**, Hyphal tip of SG200 derived strain expressing the indicated fusion proteins growing in the epidermal layer at 2 d.p.i. Yellow fluorescent protein (YFP) signal, yellow; mCherry signal, blue. Signal overlay (white) is shown on the right. Images represent maximum projections of confocal *z*-stacks. Scale bars, 5 µm. **c**, Left panel shows hyphae of AB33complex^con^ and AB33 immunostained with anti-HA primary antibodies and AF488 conjugated secondary antibody (green) without prior permeabilization. Right panel shows an overlay with differential interference contrast (DIC). Scale bars, 5 µm. **d**, Silver-enhanced immunogold labelling of SG200Δstp1-Stp1-HA, SG200Δstp3-Stp3-HA and SG200 in biotrophic hyphae. Transmission electron microscopy images of immunolabelled hyphal tips inside epidermal plant cells. Images confirm specificity of the antibody (no signal in SG200 hypha), and the label is in clusters about 50 nm across. Arrows indicate labelled particles. Particle size varies with silver enhancement time. Scale bars, 400 nm.

**Extended Data Fig. 5:**
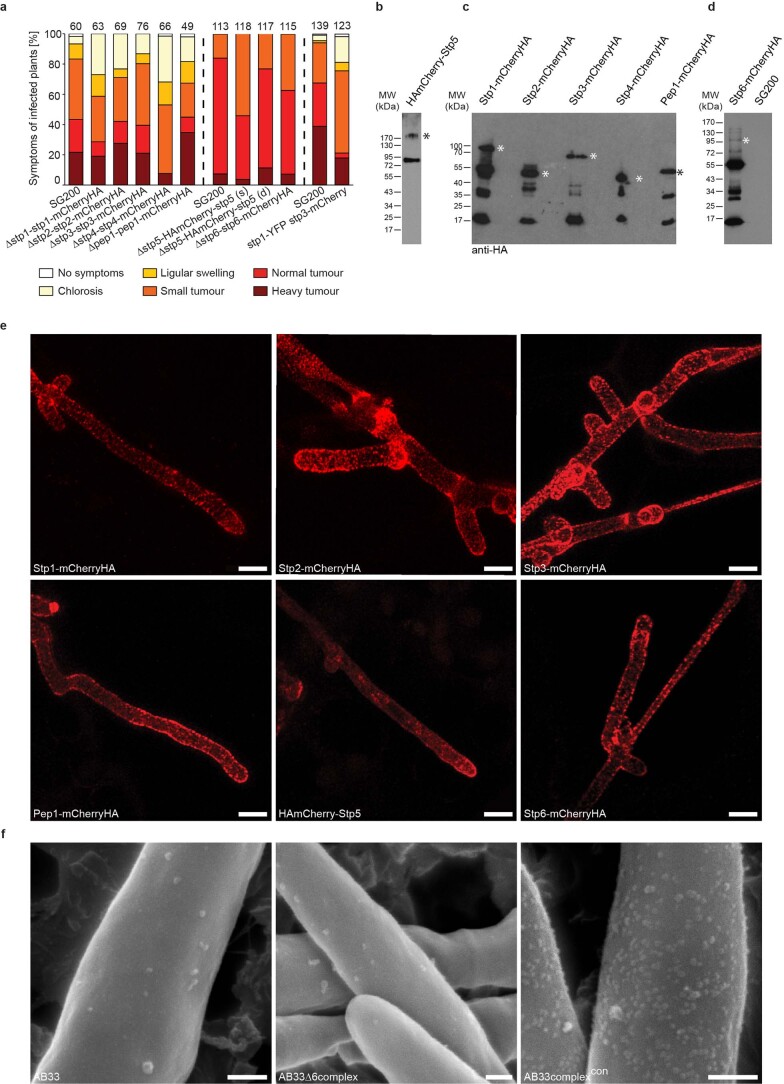
Stp-mCherry fusion proteins are functional and localize to speckles surrounding fungal hyphae after plant colonization. **a**, Seven-day-old maize seedlings were infected with *stp* deletion strains complemented with the respective mCherry fusion alleles. For *stp5* complementation strains with single (s) as well as two (d) copies of the construct are shown. Dashed lines separate independently tested groups of strains. The rightmost panel presents infections with strain SG200stp1-YFP stp3-mCherry, where both fusion proteins are expressed from genes in their native loci. 12 d.p.i. disease symptoms were scored using the scores depicted below. Data represent mean of n = 3 biologically independent experiments. Total numbers of infected plants are indicated above the respective columns. **b**, Anti-HA western blot of the total extract obtained from leaves 3 d.p.i. with strain SG200Δstp5 HAmCherry-stp5 (s). **c, d**, Anti-HA western blot after HA-specific immunoprecipitations from total maize leaf extracts prepared 3 d.p.i. with the indicated *U. maydis* strains. **b-d**, Signals corresponding to full-length fusion proteins are marked with an asterisk. **e**, Hyphal tips of SG200 strains expressing the indicated mCherry fusion proteins in the plant epidermis observed 2 d.p.i. mCherry signal: red. The images represent maximum projections of confocal z-stacks. Scale bars, 5 µm. **f**, Constitutive expression of all seven complex members changes surface topology of hyphae. Scanning electron micrographs (SEM) of AB33 and AB33Δ6complex show smooth hyphae with single scattered structures on the surface. In AB33complex^con^ a large number of small surface exposed structures are seen in addition. Scale bars, 500 nm. Source data

To localize the complex members during plant colonization, strains expressing genes for functional mCherryHA fusion proteins under the respective native promoters were constructed (Extended Data Fig. 5a). Except for HAmCherry-Stp5, which was only detected in total extracts (Extended Data Fig. 5b), full-length fusion proteins together with degradation products could be detected (Extended Data Fig. 5c,d). For microscopic analysis, a strain expressing the apoplastic effector Pit2-mCherryHA (ref. ^7^) served as control. Pit2-mCherryHA was uniformly distributed around biotrophic hyphae (Fig. 2a) while Stp1-mCherryHA, Stp2-mCherryHA, Stp3-mCherryHA, Stp4-mCherryHA, Pep1-mCherryHA and Stp6-mcherryHA all accumulated in speckles (Fig. 2a and Extended Data Fig. 5e). For HAmCherry-Stp5, a weak signal lined the hyphal tips and was detected inside hyphae (Extended Data Fig. 5e), in line with its presumed membrane localization.

**Fig. 3:**
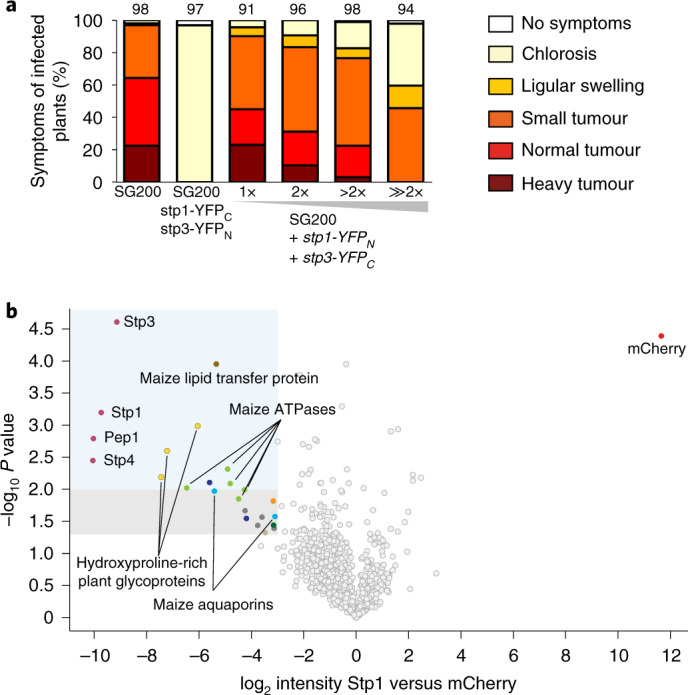
Virulence contribution of the Stp complex and plant protein interactors. **a**, Seven-day-old maize seedlings were infected with the indicated strains. Disease symptoms were scored 12 d.p.i. Strain SG200stp1-YFP_C_ stp3-YFP_N_ expresses indicated BiFC fusion proteins from genes tagged in their native locus. All other columns display SG200 with increasing numbers of ectopic insertions of *stp1*-*YFP*_*N*_ and *stp3*-*YFP*_*C*_ in addition to the native untagged copies. All data represent the mean of *n* = 3 biologically independent experiments. Total numbers of infected plants are shown above the respective columns. **b**, Plant interactors of the Stp complex shown by a volcano plot illustrating the *P* values versus log_2_ protein abundance ratios between Stp1–HA and mCherry–HA for co-IP/MS experiments, which were performed in biological triplicates with material isolated 3 d.p.i. and extracted with high detergent mix of maize infected with FB1stp1–HA × FB2stp1–HA. Displayed are all proteins for which at least four peptides were detected. Coloured dots show the indicated proteins. Significance was calculated using Student’s *t-*test. The blue- and grey-shaded areas indicate *P* values ≤0.01 and ≤0.05, respectively, and an *x*-axis value (log_2_-intensity difference of mCherry versus Stp1) below −3.

**Extended Data Fig. 6:**
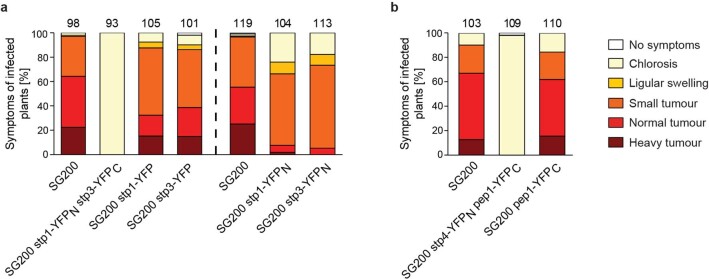
Virulence assays of strains expressing C- or N-terminal halves of YFP. Seven-day-old maize seedlings were infected with the indicated strains. 12 d.p.i. disease symptoms were scored using the colour code depicted on the right. The dashed lines indicate separate experiments. All data represent mean of n = 3 biologically independent experiments. Total numbers of infected plants are indicated above the respective columns. Infections with the control strain SG200 on the left were done in parallel with the infections shown in Fig. 3 on the left and are therefore displayed in both figures.

To determine whether the speckles contained the complex, we generated a strain expressing both Stp1-HAYFP and Stp3-mCherryHA from their native promoters (Extended Data Fig. 5a). Confocal microscopy detected co-localization of the two fusion proteins in speckles (Fig. 2b). To obtain evidence that the speckles contained the effector complex, we attempted bimolecular fluorescence complementation (BiFC). However, all strains expressing two complex members tagged with the N-terminal domain of YFP and the C-terminal domain of YFP in their respective genomic loci were unable to cause disease (Fig. 3a and Extended Data Fig. 6a,b). This suggested that the complex does not tolerate two complex members with tags that can associate. Given this strong phenotype, we next generated wild-type strains co-expressing Stp1-YFP_N_ and Stp3-YFP_C_ in different copy numbers in SG200 (that is, in a context where all untagged complex members are also present). In these strains, virulence decreased concomitantly with integrated copy number (Fig. 3a). This dominant negative phenotype indicates that complex formation is critical for virulence.

**Extended Data Fig. 7:**
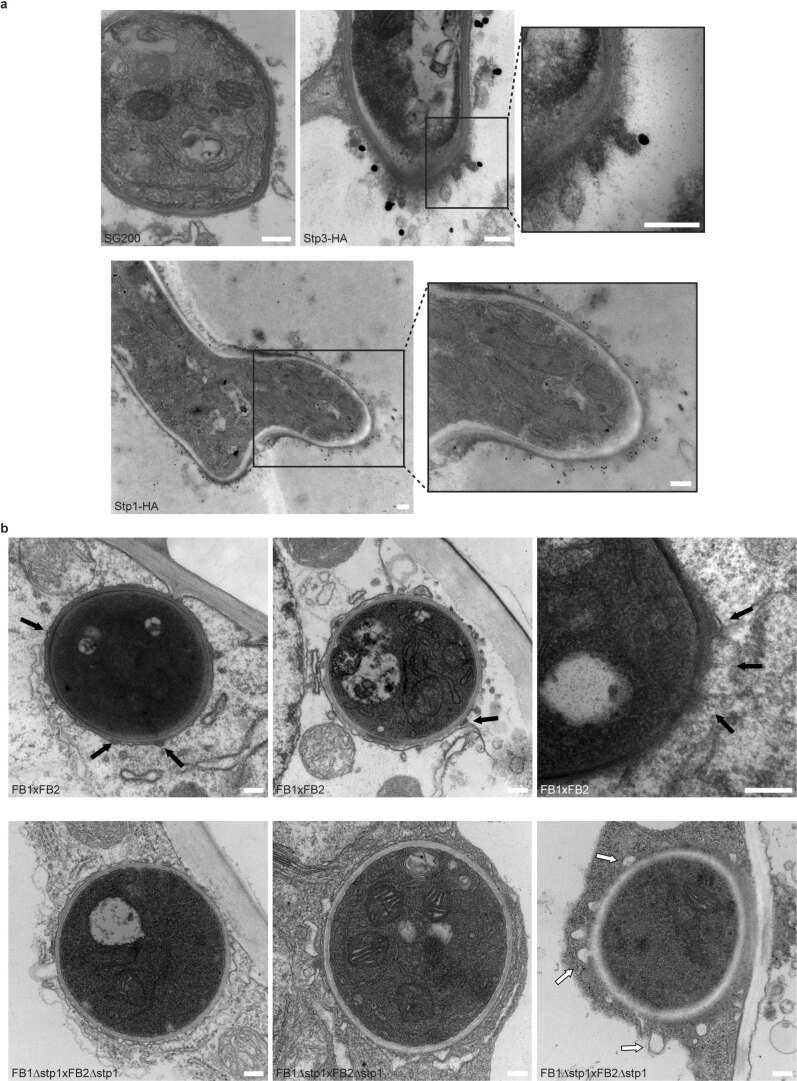
The plant-fungus interaction zone visualized by EM. **a**, Silver-enhanced immunogold labeling of Stp1-HA and Stp3-HA in biotrophic hyphae. Immuno-labeled hyphal tips inside epidermal plant cells are viewed with transmission electron microscopy. Images confirm specificity of the antibody (no signal in SG200 hyphae) and presence of signals in SG200Δstp1 Stp1-HA and SG200Δstp3 Stp3-HA. Particle size varies with silver enhancement time. **b**, Cross sections of active fungal hyphae in plant cells visualized by EM. Strains are indicated on the left. Filled arrows indicate electron-dense structures. Open arrows indicate empty vesicular structures. Note that the interaction zone in wild-type infections includes electron-dense structures that are approximately 50 nm. Scale bars, 200 nm.

To localize Stp1 and Stp3 at higher resolution, immunoelectron microscopy was performed on leaf sections infected with strains expressing HA fusion proteins from their native promoters. Antibody labelling was carried out prior to fixation and thin sectioning of maize leaves, and the only fungal hyphae accessible to the label were those inside plant cells that had been cut open so that plant cytoplasm leaked out. Here, the immunolabel was found outside the hyphal cell wall and in contact with protrusions extending into the plant cell for both Stp1-HA and Stp3-HA. Sometimes the tips of these protrusions appeared attached to membrane chunks/cell debris (Fig. 2d and Extended Data Fig. 7a). In an untagged SG200 strain, protrusions were seen, but no specific labelling was detected (Fig. 2d). When biotrophic wild-type hyphae were analysed without removal of cytosolic content, electron-dense structures were detected in the interaction zone, similarly to published observations (Extended Data Fig. 7b)^25^. The interaction zone of *stp1* mutant hyphae sometimes contained large vesicular structures in proximity of hyphae (Extended Data Fig. 7b), which could reflect attacks by the plant defence system.

Because it is only during the biotrophic interaction that all components of the Stp complex are expressed, we attempted to reconstitute the complex in filaments of the *U. maydis* strain AB33 by expressing all seven HA-tagged genes under control of different constitutive promoters selected to match the maximum expression levels of the respective genes^3^ after colonization (AB33complex^con^). Unpermeabilized filaments of AB33 and AB33complex^con^ were subjected to immunolocalization using AF488-coupled secondary HA antibodies. While fluorescent signals appeared in speckles in AB33complex^con^, AB33 was unlabelled (Fig. 2c). When filaments of these strains and, as an additional control, a derivative of AB33 lacking all complex members except *pep1* (AB33Δ6complex) was analysed by scanning electron microscope (SEM), we detected a high density of exposed structures on the surface in AB33complex^con^, the majority of them small, with some larger ones scattered among them. Such larger structures were also visible in AB33 and AB33Δ6complex, while the small structures appeared to be absent from these strains (Extended Data Fig. 5f). Given these data, it is likely that constitutive expression of the seven complex members allowed the complex to be reconstituted in a surface-exposed structure.

As we failed to detect prominent plant proteins interacting with the complex despite seeing protrusions extending into plant cells, we increased fungal biomass and improved solubilization of membrane proteins with a high detergent buffer that had previously been used successfully to extract the type IVa pilus machine proteins from *Myxococcus xanthus*^26^. With these adjustments, we identified substantially more spectral counts for Stp1-HA, Stp3, Stp4 and Pep1 after co-IP/MS (Supplementary Data 2). Combining the improved protein extraction procedure with replicated experiments allowed us to perform sensitive label-free quantification (Fig. 3b), making a strong and substantial enrichment of the four complex members evident. Surprisingly, a number of discrete plant proteins, including plasma membrane ATPases and PIP2-type aquaporins, a predicted GPI-anchored maize lipid transfer protein and hydroxyproline-rich glycoproteins, also showed elevated levels (Fig. 3b and Supplementary Data 3). This indicates that the fungal protein complex might be connected to proteins in the plant plasma membrane.

**Fig. 4:**
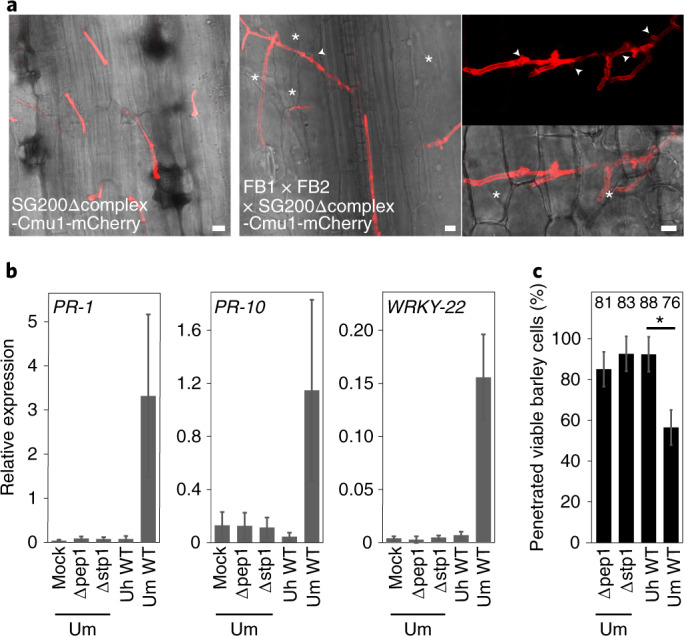
Indirect evidence for an involvement of the Stp complex in effector translocation. **a**, A mutant lacking all seven *stp* complex genes can be partially rescued by wild-type strains. Plants were co-infected by SG200Δcomplex-Cmu1-mCherry and a mixture of compatible FB1 × FB2 wild-type strains. The Δcomplex mutant was visualized by its fluorescence at 2 d.p.i. Clamp connections are indicated by arrowheads, and non-fluorescent hyphae of wild type are labelled with asterisks. Images represent maximum projections of confocal *z*-stacks. Scale bars, 10 µm. **b**, Quantitative real-time PCR of indicated barley defence genes 2 d.p.i. with strains FB1:mock, FB1Δpep1 × FB2Δpep1 (UmΔpep1), FB1Δstp1 × FB2Δstp1 (UmΔstp1) and compatible *Ustilago hordei* wild-type strains (UhWT) and FB1 × FB2 (UmWT) as controls. The vertical axis displays expression values relative to water-infected leaf samples. Data shown are the mean values of relative expression and correspond to *n* = 5 biological replicates. Error bars indicate standard deviation. **c**, Values shown are the mean of penetrated viable barley epidermal cells quantified at 2 d.p.i. after staining with fluorescein diacetate (FDA). The number above each column indicates the total number of cells counted. All experiments were performed in five biological replicates. Error bars indicate standard deviation. Asterisk indicates significance *P* = 0.0251, calculated by an unpaired two-sided *t*-test.

**Extended Data Fig. 8:**
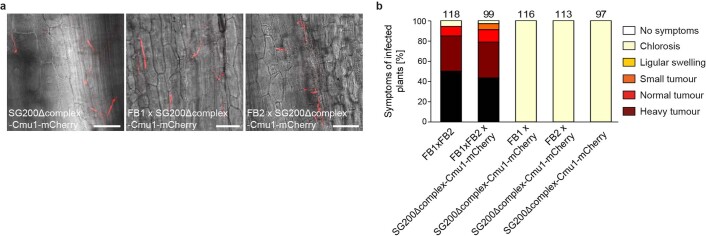
Biotrophic growth of SG200Δcomplex cannot be rescued in co-infections with haploid strains. **a**, SG200Δcomplex-Cmu1-mCherry (red) was infected alone or co-infected with either FB1 or FB2 and analysed by confocal microscopy as indicated. Growth of SG200Δcomplex-Cmu1mCherry could not be rescued by co-infections with FB1 or FB2. This illustrates that these haploid cells are unable to fuse with SG200Δcomplex-Cmu1-mCherry. Shown are maximum projections of confocal z-stacks. Scale bars, 50 µm. **b**, Maize seedlings were infected with the indicated strains. 12 d.p.i. disease symptoms were scored using the colour code shown on the right. Data represent mean of n = 3 biologically independent experiments. Total numbers of infected plants are indicated above the respective columns.

To address the function of the complex and its critical role in virulence, we considered an involvement in effector delivery, because this would fit with the protrusions extending into host cells and explain the massive defence responses seen when plants are infected with complex mutants. Biotrophic pathogens usually suppress plant cell death very efficiently by secreted effectors^27,28^. We were unable to visualize a defect in effector delivery directly and therefore tried to provide indirect evidence for an involvement of the complex. As attempts to suppress the cell death phenotype of complex mutants by chemical intervention were unsuccessful, we performed co-infections of SG200Δcomplex with a mixture of compatible untagged wild-type strains. SG200Δcomplex lacks all seven complex members but expresses a Cmu1-mCherry fusion protein and can be distinguished from wild-type cells by its fluorescence. While biotrophic hyphae of SG200Δcomplex showed the *stp* phenotype, in co-infections with compatible wild-type strains, growth of SG200Δcomplex was partially rescued, and we observed fluorescing hyphae with clamp connections and branches (Fig. 4a). Solopathogenic strains like SG200 are attenuated in mating^29^ and, consequently, neither growth nor virulence of SG200Δcomplex could be restored in co-infections with haploid FB1 or FB2 strains (Extended Data Fig. 8). In addition, we considered it highly unlikely that complementation of all seven complex components could occur by wild-type cells, which would have to provide the complex proteins in *trans*. Therefore, this result is a strong indication that wild-type hyphae have successfully downregulated plant defence responses through their arsenal of effectors, and this has allowed the complex mutant to extend its biotrophic growth. The second indirect argument for an involvement of the complex in effector delivery stems from the observation that the induction of non-host resistance is abolished in complex mutants. Non-host resistance is a common immune response likely caused by detection of non-adapted effectors^30,31^. For *U. maydis*, it has been shown previously that wild-type strains cause non-host resistance in barley and that this is associated with a massive induction of the hypersensitive response marker genes *PR-1*, *PR-10* and *WRKY-22* and cell death. By contrast, barley infection with *U. maydis pep1* mutants does not cause this response^32^. As only the peroxidase inhibitor function of Pep1 was known at the time^32^, this latter result was difficult to explain. We have now demonstrated that in addition to *pep1* mutants, *stp1* mutants also failed to elicit the expression of the hypersensitive response marker genes and death of penetrated cells (Fig. 4b,c). This can be explained by proposing that the mutants do not deliver the effectors that are recognized in barley.

## Discussion

In this study, we have identified a protein complex of seven proteins critical for the virulence of *U. maydis*. All seven proteins are present in all analysed smut species, and successful cross-species complementations for Pep1, Stp4 (Cce1) and Stp1 suggest a conserved function of the complex^24,33,34^. Formation of the complex is essential for both suppressing plant defence responses in the compatible situation and triggering non-host resistance, making it likely that the complex is required for the delivery of effectors that both downregulate these responses in host plants and are recognized in the non-host situation. Based on its insensitivity to high detergent concentrations, we believe that the Stp complex represents an ordered complex of structural proteins assembled in as yet unknown stoichiometry, present in many copies in biotrophic hyphae. That we were able to reconstitute the complex in an antibody-accessible form by expressing its seven components constitutively, together with the clustering in speckles similar to the ones seen via fluorescently tagged complex members during plant colonization, suggests a structure that is surface-exposed. Although we do not yet know whether these structures are functional, availability of such a strain will now aid in acquisition of structural data by cryo-electron tomography. The surface-exposed form is also consistent with the immunolocalization data that reveal protrusions extending into host cells. These structures might be similar to vesicular tubular structures seen in previous electron microscopy analyses of smut fungi^35^ as well as in rust fungi^36,37^. A connection between the fungal complex and the host plasma membrane seems likely given our finding of maize aquaporins and maize plasma membrane ATPase in the detergent-resistant Stp complex. While we presently do not know through which component the complex connects to the plant plasma membrane, interestingly, it has recently been shown that in rice plants, the plant plasma membrane resident aquaporin PIP1;3 and harpin Hpa1 (a translocator protein of the bacterial pathogen *Xanthomonas oryzae* pv. *oryzae* type III secretion system) interact and cooperate in effector translocation^37^. Hence, it is conceivable that the Stp complex of *U. maydis* also targets maize aquaporin to facilitate effector delivery. Plant plasma membrane ATPases, the other interactors of the Stp complex, facilitate membrane transport processes^38^ and thus might also promote effector delivery. While bacterial pathogens can use at least eight different systems for effector translocation to host cells^39^, knowledge about effector delivery by eukaryotic pathogens is restricted to apicomplexan parasites. In plasmodium parasites, many translocated effectors carry a characteristic PEXEL motif, which directs them to a distinct endoplasmic reticulum site for processing^40,41^. This step is followed by secretion into the parasitophorous vacuole, from which proteins in their unfolded state are channelled, via a translocon, into the host cell^40^. Fungal translocated effectors lack a common motif, and it is presently unknown how they are selected for delivery. In *Magnaporthe oryzae*, translocated effectors preferentially accumulate in the biotrophic interfacial complex, a membrane-rich structure of plant origin associated with biotrophic hyphae^42^. In addition, *M. oryzae* translocated effectors use a novel form of secretion involving exocyst components and the Sso1 *t*-SNARE while apoplastic effectors follow the conventional secretory pathway^43^. In *U. maydis*, the deletion of the exocyst component gene *exo70* did not affect virulence (K. Münch and R.K., unpublished observation), suggesting that a different mechanism of effector delivery is used. The finding of the Stp complex in a fungal structure extending into host cells could indicate such a device for effector delivery or the delivery of effectors in vesicles.

The essential role of all complex members in virulence and their conservation in Ustilaginaceae makes the complex a highly promising target for disease intervention. Although the complex currently appears restricted to Ustilaginaceae, we consider it likely that structural insights may reveal features that can then be used to search for related proteins in other fungal species.

## Methods

### Strain construction and growth conditions

Strains generated and used in this study are listed in Supplementary Table 1. Information concerning plasmids and how they were generated is found in Supplementary Table 2. The *Escherichia* *coli* strains DH5α (Bethesda Research Laboratories) and TOP10 (Life Technologies) were used for the cloning and amplification of plasmids. Oligonucleotides are listed in Supplementary Table 3.

Deletion mutants were generated by gene replacement using a PCR-based approach^44^. For the integration of genes into the *ip* locus, plasmids containing a carboxin resistant *ip* allele (*ip*^R^) were used^45^. These plasmids were linearized with the restriction enzymes SspI or AgeI and subsequently inserted via homologous recombination in the carboxin-sensitive *ip* allele (*ip*^S^) of *U. maydis*. If not otherwise indicated, transformed strains carrying a single insertion in the *ip* locus were generated as previously described^46^. Tagging of endogenous loci was done using CRISPR-Cas9 technology^47^.

*U. maydis* strains were grown on a rotary shaker (200 r.p.m.) at 28 °C in liquid YEPSL medium (0.4% yeast extract, 0.4% peptone and 2% sucrose), in CM medium^48^ or on PD solid medium (2.4% potato dextrose broth and 2% agar). To test filamentous growth, cell suspensions were spotted on PD-charcoal plates containing 1% activated charcoal. Stress assays were performed as previously described^49^. Transformation and selection of *U. maydis* transformants followed published procedures^18^. To assess virulence, seven-day-old maize seedlings of the maize variety Early Golden Bantam (Urban Farmer) were syringe-infected as previously described^18^. At least three independent infections were carried out, and disease symptoms were scored according to Kamper et al.^18^.

For *Saccharomyces* *cerevisiae* two-hybrid interaction studies, strain AH109 (Clontech) was used^50^. Yeast growth, transformation, selection and protein extraction were done according to the Clontech Matchmaker GAL4 Two-Hybrid user manual. To visualize proteins in western blots, mouse anti-HA monoclonal primary antibody (1:5,000 dilution; Sigma-Aldrich), mouse anti-myc monoclonal primary antibody (1:10,000 dilution; Sigma-Aldrich) and anti-mouse lgG secondary antibody (1:10,000 dilution; Cell Signaling Technology) were used.

### Staining and microscopy

Plant samples were harvested at the indicated time points and a small piece of leaf tissue was cut from about 1–2 cm below the infection mark. Either immediately or following staining, tissue pieces were placed on a glass slide in H_2_O prior to confocal microscopy.

To visualize biotrophic hyphae, infected leaf tissue was harvested at 3 d.p.i. and placed into 100% ethanol to remove the chlorophyll. Fungal hyphae were stained with Alexa Fluor 488 conjugate of wheat germ agglutinin (Invitrogen), and plant cell walls were visualized by staining with propidium iodide (Sigma-Aldrich) as previously described^51^. To stain the plant plasma membrane, infected leaf material was harvested at 1–2 d.p.i. and stained with 0.016 mM FM4-64 (Thermo Fisher) in H_2_O for 1–2 h at room temperature. To quantify the viability of barley epidermal cells that contained fungal appressoria, a microscopy-based method was used^32^. The experiment was performed in three biological replicates. An unpaired *t*-test was used (GraphPad Software, Inc.; https://www.graphpad.com) to assess statistically relevant differences.

Confocal microscopy was performed using a white light laser Leica TCS SP8x/WLL confocal laser-scanning microscope (Leica). GFP fluorescence was excited at 488 nm and detected at 495–530 nm. AF488 was excited at 488 nm and detected at 506–536 nm. Propidium iodide was excited at 561 nm and detected at 640–725 nm. FM4-64 was excited at 514 nm and detected at 623–700 nm. Calcofluor white was excited at 405 nm and detected at 425–445 nm. FDA was excited at 488 nm and detected at 495–530 nm. mCherry fluorescence was excited at 585 nm and detected at 597–635 nm. YFP fluorescence was excited at 514 nm and detected at 520–550 nm. Detection of mCherry and YFP fluorescence was achieved with a hybrid detector (HyD) where the detection time was gated (between 0.3 and 6.0 nanoseconds) after the excitation laser pulse. For image deconvolution, the HyVolution software package (Leica and Scientific Volume Imaging B.V.) was used with the highest resolution settings. Image data were processed using the Leica Application Suite X v.3.1.5. Confocal images are representative of at least three biological replicates yielding similar results.

To visualize the reconstituted complex in the filaments of AB33, the strains were shifted to the filamentous form as described previously^52^. For SEM, shifted cells were spotted on a membrane (Hybond-N+, GE Healthcare) placed on a nitrate-minimal-agar plate and kept at 28 °C for several hours. Specimens were then fixed in 4% glutaraldehyde in Sörensen buffer pH 7.8 overnight before being washed six times with distilled water for 10 min each. Then, the specimens were washed a further six times, for 10 min each time, with water–acetone mixtures with increasing percentages of acetone (30, 50, 75, 90, 95 and 100%; the last step was repeated twice). Specimens were critical point dried (Polaron E 3000), fixed on stubs with electrically conducting adhesive pads and sputter coated (Balzers Union) with gold. Specimens were observed with a Hitachi S-530 SEM at 25 kV. For immunolocalization, the staining was performed according to Ma et al.^10^. The shifted cells were washed with PBS, blocked in 3% BSA and subsequently incubated in PBS containing mouse anti‐HA antibody (1:1,500 dilution; Sigma‐Aldrich) and 3% BSA at 4 °C overnight. The cells were washed three times with PBS and incubated in PBS containing goat anti‐mouse IgG secondary antibody conjugated with Alexa Fluor 488 (1:15,000 dilution; Life Technologies) for 1 h at room temperature. After washing three times with PBS, the cells were analysed by confocal laser‐scanning microscopy. Images are representative of at least three biological replicates yielding similar results.

For immuno-transmission electron microscopy, cross-sections of maize leaves were cut 36–48 hours after they had been inoculated with *U. maydis* strains expressing Stp3-HA or Stp1-HA from their respective native promoters. The HA-tagged proteins were localized prior to resin embedment and sectioning for electron microscopy, adapting the technique described by Sesack et al.^53^. Briefly, sections were fixed in 0.5% glutaraldehyde and 4% freshly prepared formaldehyde in pH 7.4 phosphate buffer for 30 min, rinsed, blocked and labelled with the primary antibody (1:60 dilution; HA tag monoclonal antibody 2-2.2.14, Invitrogen) in blocking buffer for 8 h. After rinsing, leaf pieces were incubated overnight in secondary antibody conjugated to ultra-small gold (1:50 dilution; Electron Microscopy Sciences). After rinsing, leaf pieces were fixed in 2% glutaraldehyde in pH 7.4 phosphate buffer for 15 min. The small gold particles were then enhanced with silver, using methods and reagents as provided in the Aurion R-Gent Silver Enhancement kit (AURION Immuno Gold Reagents & Accessories). Only maize cells that were cut open in preparation could be labelled, because the antibodies and silver enhancement reagents did not penetrate intact plant cell walls. After enhancement, sections were briefly postfixed in osmium tetroxide, dehydrated, infiltrated and embedded in Ultrabed Low Viscosity resin (Electron Microscopy Sciences). Longer enhancement and osmication times favoured larger particle size and better membrane visualization, but shorter times favoured retention of the silver-enhanced particles. Sectioning and imaging were performed as described elsewhere^54^. Images are representative of at least two independent experimental replicates yielding similar results.

### Protein extraction and co-IP from infected plant material

For co-IPs, samples were harvested by excising infected plant parts and shock freezing in liquid nitrogen before being stored at −80 °C. Frozen plant material was ground to a fine powder using a prechilled mortar and pestle or a Retsch CryoMill. For protein extraction, 1 g of frozen powder was added to a prechilled mortar containing 4 ml of ice-cold HNN lysis buffer and approximately 100 µl of 0.1 mm silica spheres (Lysing Matrix B, Bulk MP Biomedicals). HNN lysis buffer (50 mM HEPES pH 7.5, 150 mM NaCl, 50 mM NaF, 5 mM EDTA, 0.1% NP-40 and 1% polyvinylpyrrolidone). Prior to use, one tablet of cOmplete (Mini Protease Inhibitor, Merck) was added to 50 ml of HNN lysis buffer. After being ground in buffer with a chilled pestle, samples were transferred to fresh 5 ml tubes and incubated on ice for 30 min. After incubation, samples were centrifuged at 4 °C for 15 min at 20,000 *g*, and the resulting supernatant was transferred to a fresh tube. After adding 12 µl of magnetic beads (Pierce Anti-HA magnetic beads, Thermo Fisher Scientific), the samples were incubated for 1 h at 4 °C with rotation. The magnetic beads were separated on a magnetic separator. In experiments using the detergent-rich buffer, 1 g of ground powder was transferred to 2 ml of the detergent-rich buffer (50 mM HEPES pH 7.5, 150 mM NaCl, 50 mM NaF, 5 mM EDTA, 1% polyvinylpyrrolidone, 4% CHAPS, 4% Zwittergent 3-14, 4% *N*-lauroylsarcosine, 0.05% NP-40 and 20% glycerol), to 50 ml of which one tablet of cOmplete (Mini Protease Inhibitor, Merck) was added, and incubated for 10 min at room temperature. Afterwards, the solution was diluted 1:6 with HNN to reduce all detergent to less than 1%, then was incubated for 1 h at 4 °C. The subsequent steps were identical to those for samples prepared in HNN buffer.

IPs followed by western blots were done with HNN lysis buffer, skipping the second grinding step. The antibodies used were rabbit anti-HA primary antibody (1:10,000 dilution; Sigma-Aldrich) and anti-rabbit lgG secondary antibody (1:10,000 dilution; Cell Signaling Technology).

### Identification of proteins by liquid chromatography–mass spectrometry (LC–MS)

Sample preparation of purified proteins and LC–MS proteomics analysis were performed as described previously, with minor modifications^55^. In brief, an on-bead tryptic digest (Mass Spectrometry Grade Trypsin, Promega) was carried out on protein enrichments with subsequent reverse-phase C18 solid phase extraction using Minispin columns (Harvard Apparatus). Peptides eluted from the spin columns were dried, reconstituted in 0.1% trifluoroacetic acid and subjected to LC–MS analysis.

LC–MS analysis of the peptide samples was carried out on a Q-Exactive Plus instrument in conjunction with an Ultimate 3000 RSLC nano and a nanospray flex ion source (all Thermo Fischer Scientific). A 42 cm self-packed C18 column was used for peptide separation using a 2–35% acetonitrile gradient prior to the Orbitrap-MS analysis. The MS analysis was performed using settings identical to those described by Gómez-Santos et al.^55^.

For the database search, the combined protein database for *U. maydis* and *Zea mays* downloaded from Uniprot (https://www.uniprot.org) was used. Spectral counting based analysis was performed using MASCOT v.2.5 executed from the Proteome Discoverer platform v.1.4 (Thermo Scientific). The following search parameters were used: full tryptic specificity required (cleavage after lysine or arginine residues), two missed cleavages allowed, carbamidomethylation (C) set as a fixed modification and oxidation (M) and deamidation (N,Q) set as a variable modification. The mass tolerance was set to 10 ppm for precursor ions and 0.02 Da for high energy-collision dissociation (HCD) fragment ions. The results were then imported into Scaffold v.4.6.2 (Proteome Software), and a 1% false discovery rate was adjusted within the Scaffold software.

Twenty bait purifications (3× Stp1-HA, 3× Stp2-HA, 3× Pep1-HA, 3× Stp6-HA, 4× Stp3-HA and 4× Stp4HA) versus an equal number of control experiments were used to detect a set of specifically bait-interacting proteins. Protein spectral counts from identified interactors were extracted and ‘0’ spectral counts were replaced with the background value of 0.5, then log_2_ ratios of spectral counts were calculated between baits and mCherry control IPs. The log ratio values were then *z* transformed. In order to identify potential protein interactors, proteins enriched in a bait purification required a minimum *Z*-score of 2 in at least three experiments for each bait, the only exception being Stp6, which showed slightly higher variations. For Stp6 IP experiments, proteins were considered enriched when a minimum *Z*-score of 2 was detected in two out of three replicate experiments. The *U. maydis* effector protein components surviving the described filtering criteria were visualized in Cytoscape v.3.3.0. Average spectral counts of identified complex members across experiments are represented by edge width.

Label-free quantification (LFQ) of the samples was performed using MaxQuant v.1.6.3.4. For Andromeda database searches, the forward protein databases for *U. maydis* and *Z. mays* obtained from Uniprot were used. The search was carried out using full tryptic specificity while allowing two missed cleavages. Carbamidomethylation (C) was set as fixed modification; oxidation (M) and deamidation (N, Q) as variable modification. MaxQuant was operated with default settings. To calculate protein enrichment in co-IP experiments, MaxQuant-LFQ values were loaded into Perseus v.1.5.2.6 (ref. ^56^). As a basis for log ratio calculation between the conditions, missing values were imputed using the imputation function from normal distribution implemented in Perseus with default settings (width, 0.3; down-shift, 1.8).

### RACE PCR

Total RNA was extracted from leaves infected with SG200 and harvested 3 d.p.i. using TRIzol reagent (Invitrogen). The 3′ RACE System for Rapid Amplification of cDNA Ends (Invitrogen) was used to perform 3′ RACE PCR. cDNA was prepared using 5 µg total RNA and the adapter primer (AP) provided in the kit. The first 3′ RACE PCR was performed on the cDNA using primer pairs oCG57 and AUAP and the following reaction conditions: 5 min at 98 °C followed by 35 cycles of the sequence 10 s at 98 °C, 30 s at 59 °C, 3 min at 72 °C and 5 min at 72 °C. Then, 1 µl of product was used as a template for nested PCR using oligonucleotides oCG63/AUAP and Phusion DNA polymerase (Invitrogen) and the following reaction conditions: 5 min at 98 °C followed by 35 cycles of the sequence 10 s at 98 °C, 30 s at 61 °C, 3 min at 72 °C and 5 min at 72 °C. This yielded two products, one 3 kilobase (kb) product and one 1.6 kb product. The 1.6-kb product could be sequenced and was shown to correspond to the polyadenylated product of *stp6s* after sequencing with oligonucleotide oCG65. To determine the nature of the larger fragment, PCR reactions were also performed on cDNA using primer pair oCG57/oCG62, revealing a 2.8-kb product corresponding to a region of *stp6* containing three introns. To determine the structure of the 3′ part of *stp6*, the 3′ RACE PCR was repeated on cDNA with primer pair oCG47/AUAP. Reaction conditions were 5 min at 98 °C followed by 35 cycles of 10 s at 98 °C, 30 s at 67 °C, 35 s at 72 °C and 5 min at 72 °C. The products were used as a template for a nested PCR with primers oCG71/AUAP, yielding a 1.2-kb fragment that could be sequenced using primer oCG71. This fragment contained the 3′ end of *stp6*, including the polyA tail after nucleotide 3361.

### Visualizing secretion and membrane localization of Stp proteins

To visualize secretion of Stp2-HA, Stp3-HA and Stp4-HA, AB33-derived strains expressing the respective genes constitutively from the *otef* promoter were generated and shifted to the filamentous form following published procedures^52^. The formation of *b* filaments was followed microscopically. Cultures were centrifuged 6 h after the shift, and cell pellets were kept at −20 °C. For western blot analysis, pellets were resuspended in sample buffer (50 mM Na-HEPES pH 7.5, 200 mM sodium acetate pH 7.5, 1 mM EDTA, 1 mM EGTA, 5 mM magnesium acetate, 5% glycerol, 0.25% NP-40, 3 mM DTT and 1 mM PMSF). Prior to use, one tablet of cOmplete (Mini Protease Inhibitor, Merck) was added to 50 ml of the suspension. Cells were disrupted using a Fast Prep-24 Instrument (MP Biomedicals), boiled for 5 min and centrifuged for 1 min at 13,000 r.p.m. at room temperature. To analyse supernatant fractions by western blot, trichloroacetic acid was added to a final concentration of 20% to cell-free supernatants from the initial centrifugation. Samples were kept overnight at 4 °C and then centrifuged at 8,000 r.p.m. for 2 h at 4 °C. Pellets were washed in 2 ml ice-cold acetone and transferred to a 5 ml microcentrifuge tube. After three additional washes with 5 ml of ice-cold acetone, pellets were dried at 60 °C, resuspended in 40 µl of sample buffer and subjected to western blot analysis. For HA protein fusion detection, rabbit anti-HA primary antibody (1:10,000 dilution; Sigma-Aldrich) and anti-rabbit lgG secondary antibody (1:10,000 dilution; Cell Signaling Technology) were used. For lysis control, anti-α-tubulin monoclonal primary antibody from mouse (1:2,000 dilution; Calbiochem) and anti-mouse lgG secondary antibody (1:10,000 dilution; Cell Signaling Technology) were used.

For SG200Potef-HA-Stp5, SG200Potef-Stp6-HA and SG200∆kex2-Stp1-HA expressing the respective genes from the constitutive *otef* promoter cells and were propagated to an optical density at 600 nm (OD_600_) of 0.8 in CM (2% glucose) to which one tablet of cOmplete (Mini Protease Inhibitor, Merck) was added per 50 ml. Proteins in the pellet fraction as well as in the supernatant were isolated as described for AB33-derived strains.

To visualize membrane localization of Stp5 and Stp6, SG200Potef-HA-Stp5 and SG200Potef-Stp6-HA were grown in YEPSL until an OD_600_ of 0.8 and fractionated into a supernatant fraction containing soluble proteins and a plasma membrane containing the pellet fraction, as previously described^57^. The pellet fraction was resuspended in 10 ml of extraction buffer to which both Triton X-100 and SDS were added to final concentrations of 2%. The suspension was kept on ice for 30 min and centrifuged at 22,000 r.p.m. for 10 min at 4 °C to yield a supernatant fraction containing solubilized membrane proteins.

### PR gene expression upon non-host infection

For the analysis of PR gene expression, barley plants of the variety Golden Promise were cultivated in a phytochamber at 60% relative humidity with a 16 h light period (22 °C, 30,000 lux) and 8 h dark period (18 °C). For barley infections, *U. maydis* cultures were grown at 28 °C and *U. hordei* (4857‐4 Mat‐1 and 4857‐5 Mat‐2) cultures were grown at 22 °C to an OD_600_ of 0.8–1.5. Cells were harvested for 10 min at 1,000 *g* and resuspended in 0.1% Tween20 to an OD_600_ of 3. Next, 300 µl of 1:1 mixtures of the respective compatible strains were needle-inoculated into the base of leaf whorls of 11-day-old barley seedlings using a syringe. At 48 h post infection, leaf sections from 2 cm below the infection holes to the beginning of the leaf sheet were excised from the second and third leaves from 13 plants, combined, frozen in liquid nitrogen and ground to a fine powder with a mortar and pestle cooled in liquid nitrogen. RNA extraction and RT-qPCR of HvPR1, HvPR19, HvWRKY22 and HvGAPDH were carried out as previously described^32^. The experiment was done in five biological and two technical replicates, and gene expression levels were calculated relative to the expression levels of the constitutively expressed barley glyceraldehyde dehydrogenase gene and the target gene expression level in water-infected plants using the ΔΔCt method^58^.

### Data and bioinformatic analyses

Microsoft Excel 2013 was used for data analysis. For genomes lacking annotations, amino acid sequences were extracted with the program CLC benchtop by tBlastn blast of the genome sequence data using the respective *U. maydis* or the *U. hordei* (barley) protein as a reference and the program CLC genomics workbench (Qiagen v.9.5.3) in combination with the Augustus gene prediction program (http://bioinf.uni-greifswald.de/augustus/)^59^. Protein and DNA sequences of *stp* orthologues from 11 sequenced smuts were derived from the following sources: *Ustilago esculenta* accession number JTLW00000000, version JTLW01000000 (ref. ^60^); *Ustilago trichophora* RK089 accession number LVYE00000000, version LVYE01000000 (ref. ^61^); *Ustilago tritici* accession number NSHH00000000, version NSHH01000000 (ref. ^62^); *Sporisorium reilianum* f.sp. *zeae* SRZ2 accession number FQ311430-FQ311474 (ref. ^63^), *Sporisorium reilianum* f.sp. *reilianum* SRS1_H2-8 accession number LT795054-LT795076 (refs. ^64,65^), *Sporisorium scitamineum* Sscl8 accession number LK056649-LK056695 (ref. ^66^); *U. maydis* 521, https://mycocosm.jgi.doe.gov/Ustma2_2/Ustma2_2.home.html (ref. ^18^); *U. hordei* Uhor01 accession number NSDP00000000 version NSDP01000000 (ref. ^62^); *U. hordei* Uh4875-4 accession number CAGI01000001-CAGI01000713 (ref. ^67^); *Ustilago bromivora* UB2112 accession number PRJEB7751 (ref. ^68^); *M. pennsylvanicum* Mp4 project accession number PRJEB4565, accession number HG529494-HG529928 (ref. ^69^). *M. pennsylvanicum* Mp4 was resequenced by PacBio and gene information from this project was included (R.K., unpublished).

Nucleotide sequences were aligned using Clustal Omega provided by the Swiss Institute of Bioinformatics (https://www.ebi.ac.uk/Tools/msa/clustalo/)^70^, and the multiple nucleotide alignment file was processed with BOXSHADE v.3.21 provided by the EMBnet (https://embnet.vital-it.ch/software/BOX_form.html). Signal peptide prediction was performed with SignalP v.5 (http://www.cbs.dtu.dk/services/SignalP/)^71^. The occurrence of transmembrane domains was predicted using TMHMM v.2.0 (http://www.cbs.dtu.dk/services/TMHMM/)^72^.

### Reporting Summary

Further information on research design is available in the Nature Research Reporting Summary linked to this article.

## Supplementary information

Supplementary Information Supplementary Figs. 1–7, Tables 1–3 and references.

Reporting Summary

Supplementary Data 1 Table of spectrum counts per protein across all co-IP/MS experiments.

Supplementary Data 2 Table of spectrum counts per protein identified after co-IP/MS experiments with Stp1-HA after infection with FB1∆stp1-stp1-HA × FB2∆stp1-stp1-HA.

Supplementary Data 3 LFQ data matrix exported from Perseus after data imputation and significance test of mCherry versus Stp1 co-IP/MS experiments.

## Data Availability

*U. maydis* genes and encoding protein sequences are available at NCBI under the following accession numbers: *U. maydis stp1* (UMAG_02475), XP_011388756.1; *U. maydis stp2* (UMAG_10067), XP_011388794.1; *U. maydis stp3* (UMAG_00715), XP_011386505.1; *U. maydis stp4* (UMAG_12197), XP_011389576.1; *U. maydis pep1* (UMAG_01987), XP_011387901.1; *U. maydis stp5* (UMAG_04342), XP_011391052.1; *U. maydis stp6* (UMAG_01695), XP_011387671.1. Source data are provided with this paper.

## Source data

Source Data Extended Data Fig. 1 Unprocessed western blots. Source Data Extended Data Fig. 2 Unprocessed western blots and gels. Source Data Extended Data Fig. 3 Unprocessed western blots. Source Data Extended Data Fig. 4 Unprocessed western blots. Source Data Extended Data Fig. 5 Unprocessed western blots.
