# Supplementary material for: A cell surface-exposed protein complex with an essential virulence function in Ustilago maydis
Source: Nat Microbiol. 2021 May 3;6(6):722–30. doi: 10.1038/s41564-021-00896-x (PMC8159752; doi:10.1038/s41564-021-00896-x)
Supplement: Supplementary file 1 — Supplementary Figs. 1–7, Tables 1–3 and references. [file 41564_2021_896_MOESM1_ESM.pdf]

nature portfolio

https://doi.org/10.1038/s41564-021-00896-x

Supplementary information

A cell surface-exposed protein complex with an essential virulence function in *Ustilago maydis*

In the format provided by the authors and unedited

Supplementary Information

Article title: **A cell surface-exposed protein complex with an essential virulence function in *Ustilago maydis***

Authors: Nicole Ludwig, Stefanie Reissmann, Kerstin Schipper, Carla Gonzalez, Daniela Assmann, Timo Glatter, Marino Moretti, Lay-Sun Ma, Karl-Heinz Rexer, Karen Snetselaar, and Regine Kahmann

Supplementary Figures

Supplementary Fig. 1: Alignment of Stp1 orthologues from 11 sequenced smut fungi.

Supplementary Fig. 2: Alignment of Stp2 orthologues from 11 sequenced smut fungi.

Supplementary Fig. 3: Alignment of Stp3 orthologues from 11 sequenced smut fungi.

Supplementary Fig. 4: Alignment of Stp4 orthologues from 11 sequenced smut fungi.

Supplementary Fig. 5: Alignment of Pep1 orthologues from 11 sequenced smut fungi.

Supplementary Fig. 6: Alignment of Stp5 orthologues from 11 sequenced smut fungi.

Supplementary Fig. 7: Alignment of Stp6 orthologues from 11 sequenced smut fungi.

Supplementary Tables

Supplementary Table 1: Strains used in the study.

Supplementary Table 2: Plasmids and gBlocks used in the study.

Supplementary Table 3: Oligonucleotides used in the study.

Supplementary Information References

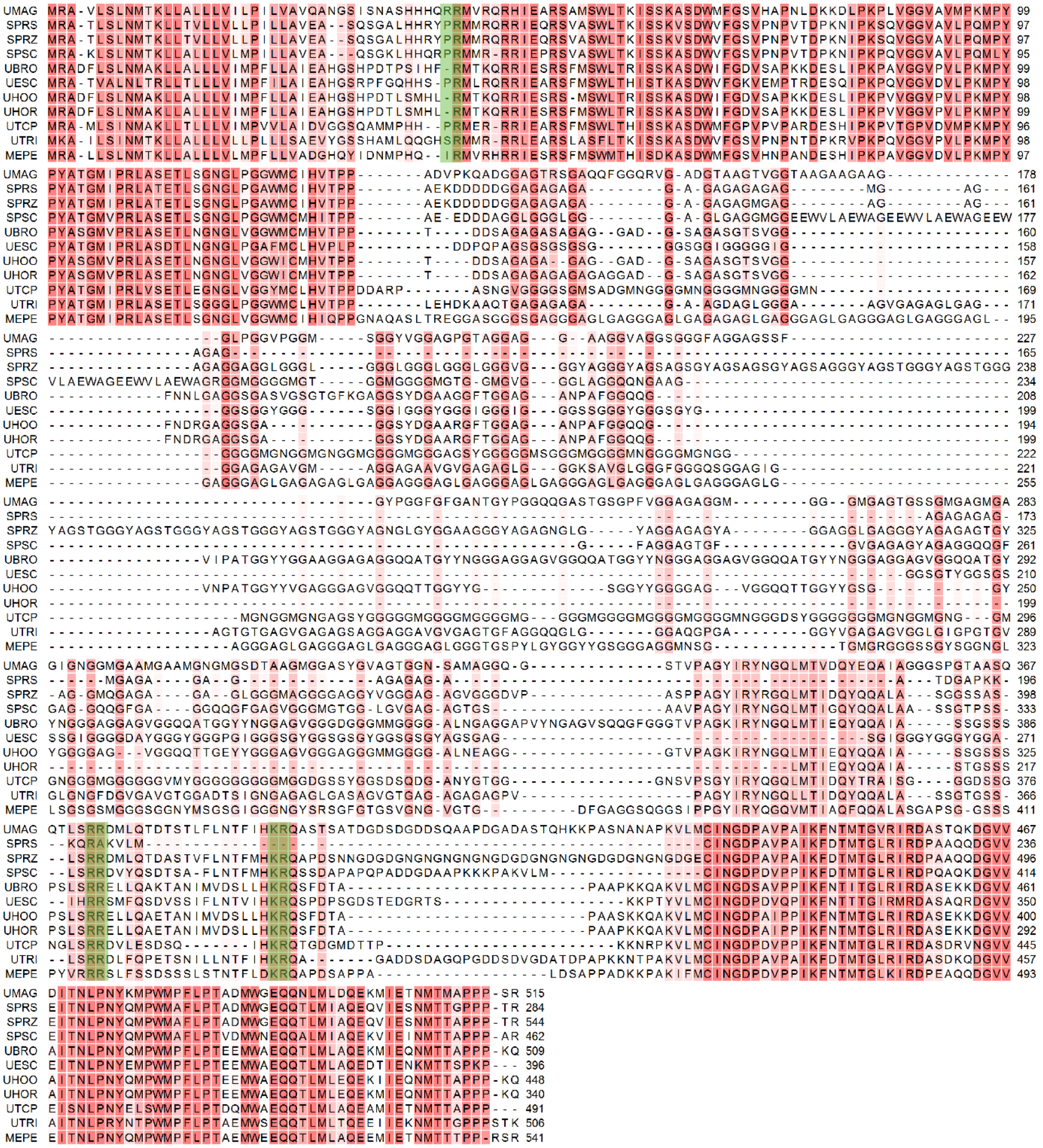

Supplementary Fig.1 Alignment of Stp1 orthologues from 11 sequenced smut fungi. For genomes lacking annotation amino acid sequences were extracted with the program CLC benchtop by running tBlastn predictions of the genome sequence data using the respective *U. maydis* or the *U. hordei* (host: barley) protein as a reference. The alignments were generated with CLC benchtop. Red background colour indicates conservation between more than 50% of the sequences. The darker the colour the higher the conservation. MEPE: *Melanopsichium pennsylvanicum*; UESC: *Ustilago esculenta*; SPSC: *Sporisorium scitamineum*; SPRZ:

Sporisorium reilianum f. sp. *zeae*; SPRS: *Sporisorium reilianum* f. sp. *sorghi*; UTCP: *Ustilago trichophora*; UTRI: *Ustilago tritici*; UMAG: *U. maydis*; UBRO: *Ustilago bromivora*; UHOR: *Ustilago hordei* (host: barley); UHOO: *Ustilago hordei* (host: oats). Putative Kex2 cleavage sites are highlighted in green.

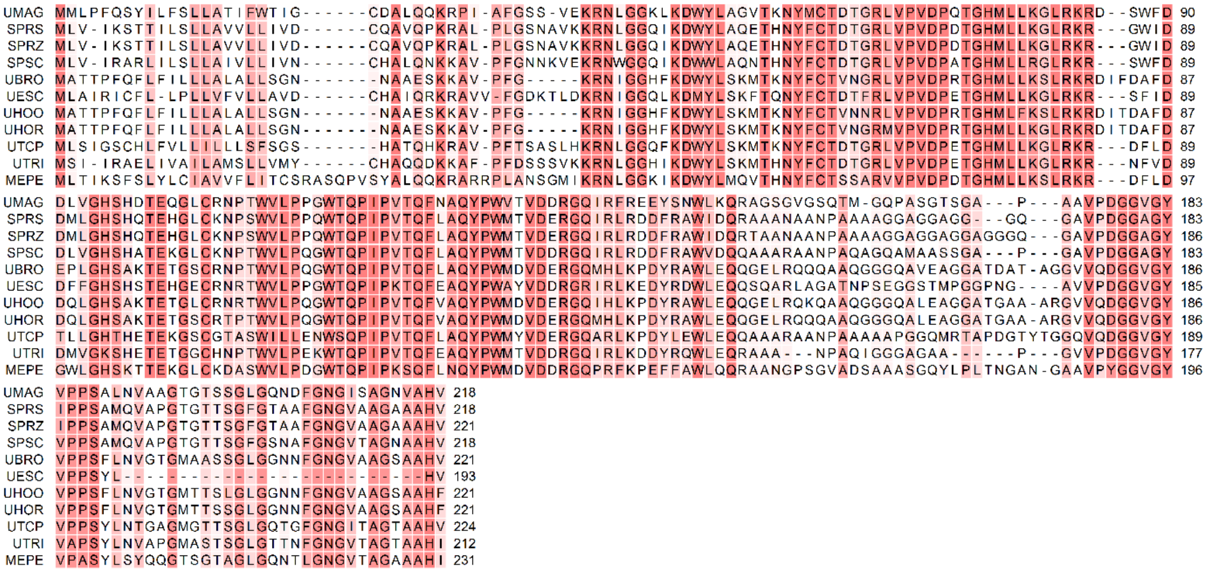

Supplementary Fig.2 Alignment of Stp2 orthologues from 11 sequenced smut fungi. For genomes lacking annotation amino acid sequences were extracted with the program CLC benchtop by running tBlastn predictions of the genome sequence data using the respective *U. maydis* or the *U. hordei* (host: barley) protein as a reference. The alignments were generated with CLC benchtop. Red background colour indicates conservation between more than 50% of the sequences. The darker the colour the higher the conservation. MEPE: *Melanopsichium pennsylvanicum*; UESC: *Ustilago esculenta*; SPSC: *Sporisorium scitamineum*; SPRZ: *Sporisorium reilianum* f. sp. *zeae*; SPRS: *Sporisorium reilianum* f. sp. *sorghi*; UTCP: *Ustilago trichophora*; UTRI: *Ustilago tritici*; UMAG: *U. maydis*; UBRO: *Ustilago bromivora*; UHOR: *Ustilago hordei* (host: barley); UHOO: *Ustilago hordei* (host: oats).

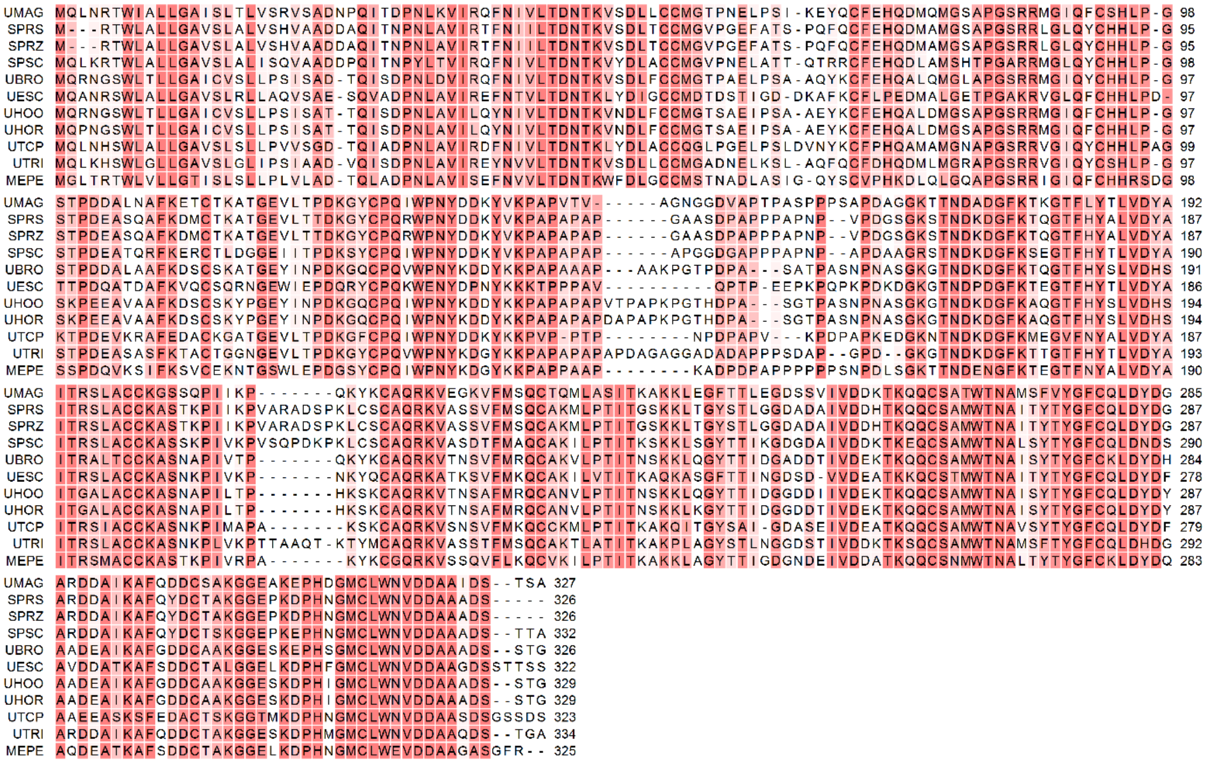

Supplementary Fig.3 Alignment of Stp3 orthologues from 11 sequenced smut fungi. For genomes lacking annotation amino acid sequences were extracted with the program CLC benchtop by running tBlastn predictions of the genome sequence data using the respective *U. maydis* or the *U. hordei* (host: barley) protein as a reference. The alignments were generated with CLC benchtop. Red background colour indicates conservation between more than 50% of the sequences. The darker the colour the higher the conservation. MEPE: *Melanopsichium pennsylvanicum*; UESC: *Ustilago esculenta*; SPSC: *Sporisorium scitamineum*; SPRZ: *Sporisorium reilianum* f. sp. *zeae*; SPRS: *Sporisorium reilianum* f. sp. *sorghi*; UTCP: *Ustilago trichophora*; UTRI: *Ustilago tritici*; UMAG: *U. maydis*; UBRO: *Ustilago bromivora*; UHOR: *Ustilago hordei* (host: barley); UHOO: *Ustilago hordei* (host: oats).

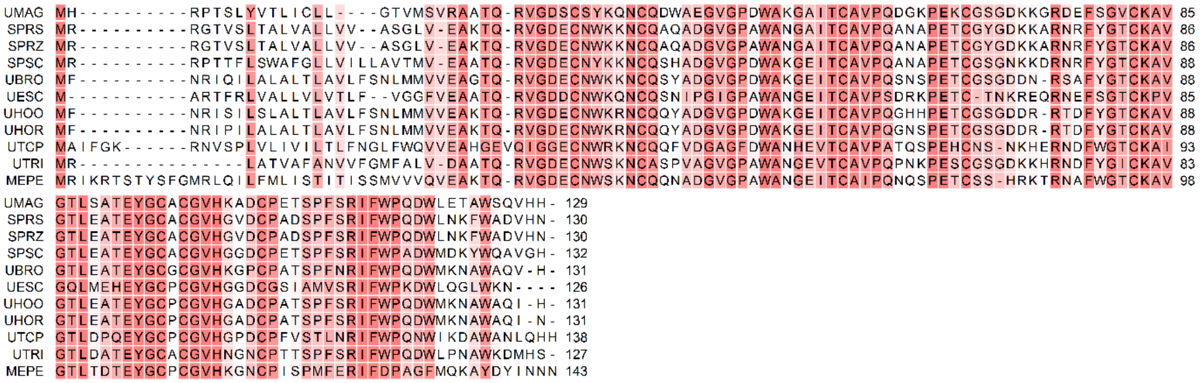

Supplementary Fig.4 Alignment of Stp4 orthologues from 11 sequenced smut fungi. For genomes lacking annotation amino acid sequences were extracted with the program CLC benchtop by running tBlastn predictions of the genome sequence data using the respective *U. maydis* or the *U. hordei* (host: barley) protein as a reference. The alignments were generated with CLC benchtop. Red background colour indicates conservation between more than 50% of the sequences. The darker the colour the higher the conservation. MEPE: *Melanopsichium pennsylvanicum*; UESC: *Ustilago esculenta*; SPSC: *Sporisorium scitamineum*; SPRZ: *Sporisorium reilianum* f. sp. *zeae*; SPRS: *Sporisorium reilianum* f. sp. *sorghi*; UTCP: *Ustilago trichophora*; UTRI: *Ustilago tritici*; UMAG: *U. maydis*; UBRO: *Ustilago bromivora*; UHOR: *Ustilago hordei* (host: barley); UHOO: *Ustilago hordei* (host: oats).

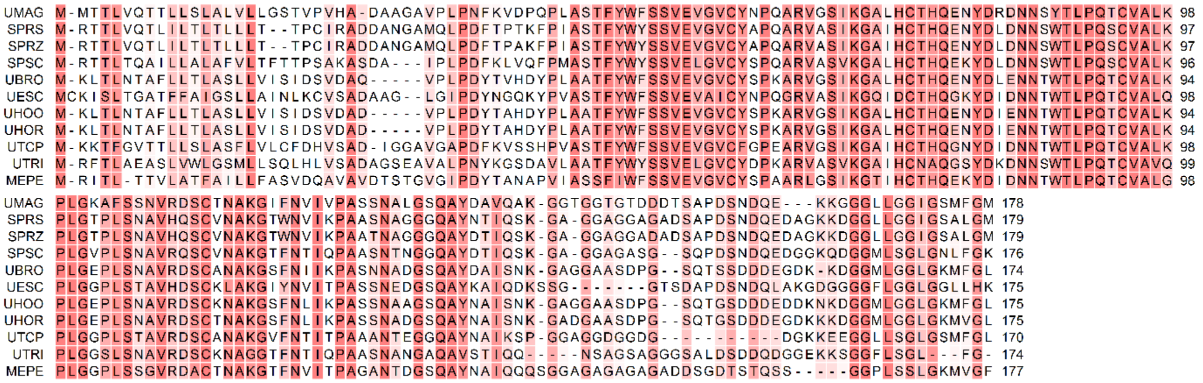

Supplementary Fig.5 Alignment of Pep1 orthologues from 11 sequenced smut fungi. For genomes lacking annotation amino acid sequences were extracted with the program CLC benchtop by running tBlastn predictions of the genome sequence data using the respective *U. maydis* or the *U. hordei* (host: barley) protein as a reference. The alignments were generated with CLC benchtop. Red background colour indicates conservation between more than 50% of the sequences. The darker the colour the higher the conservation. MEPE: *Melanopsichium pennsylvanicum*; UESC: *Ustilago esculenta*; SPSC: *Sporisorium scitamineum*; SPRZ: *Sporisorium reilianum* f. sp. *zeae*; SPRS: *Sporisorium reilianum* f. sp. *sorghi*; UTCP: *Ustilago trichophora*; UTRI: *Ustilago tritici*; UMAG: *U. maydis*; UBRO: *Ustilago bromivora*; UHOR: *Ustilago hordei* (host: barley); UHOO: *Ustilago hordei* (host: oats).

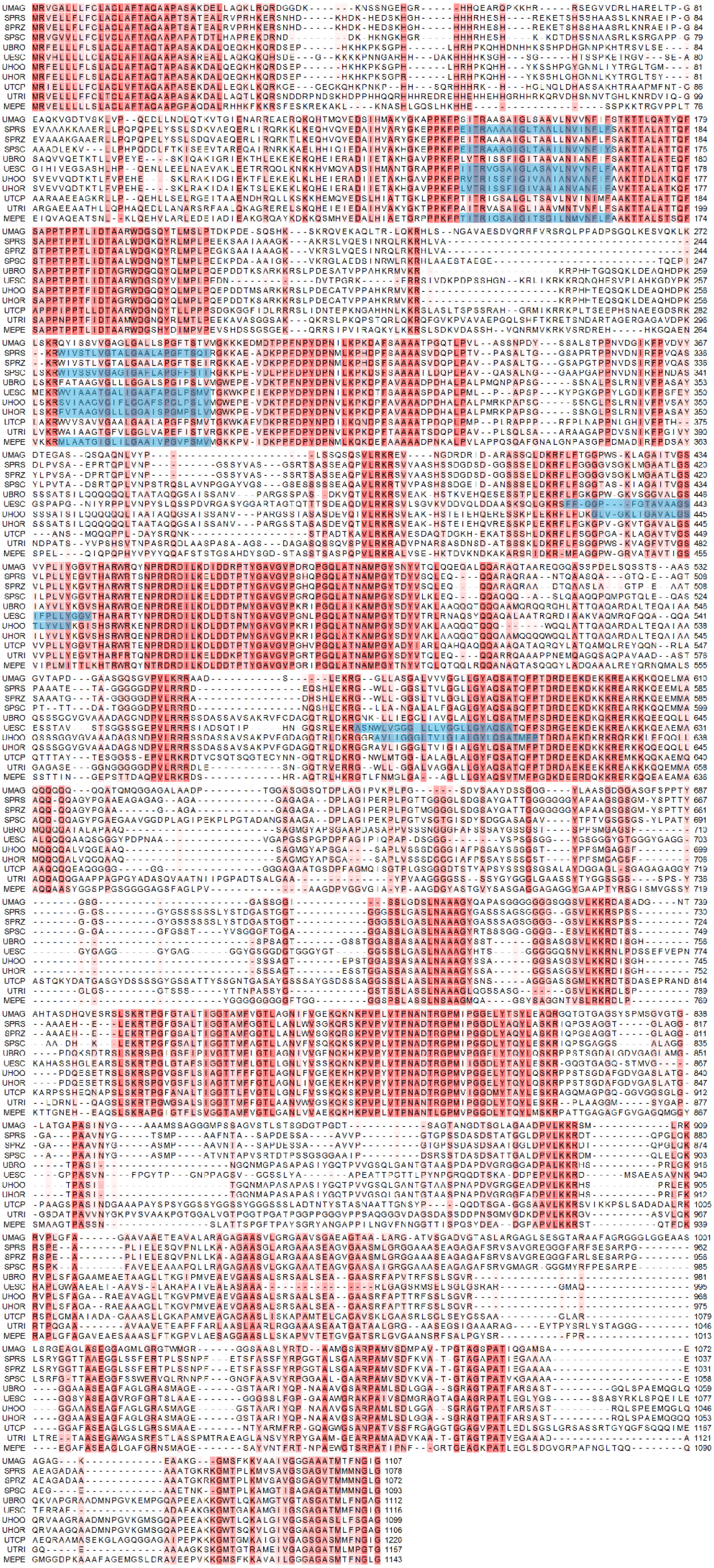

Supplementary Fig.6 Alignment of Stp5 orthologues from 11 sequenced smut fungi. For genomes lacking annotation amino acid sequences were extracted with the program CLC benchtop by running tBlastn predictions of the genome sequence data using the respective *U. maydis* or the *U. hordei* (host: barley) protein as a reference. The alignments were generated with CLC benchtop. Red background colour indicates conservation between more than 50% of the sequences. The darker the colour the higher the conservation. MEPE: *Melanopsichium pennsylvanicum*; UESC: *Ustilago esculenta*; SPSC: *Sporisorium scitamineum*; SPRZ: *Sporisorium reilianum* f. sp. *zeae*; SPRS: *Sporisorium reilianum* f. sp. *sorghi*; UTCP: *Ustilago trichophora*; UTRI: *Ustilago tritici*; UMAG: *U. maydis*; UBRO: *Ustilago bromivora*; UHOR: *Ustilago hordei* (host: barley); UHOO: *Ustilago hordei* (host: oats). Transmembrane domains predicted by TMHMM Server, V2.0 are highlighted in blue.

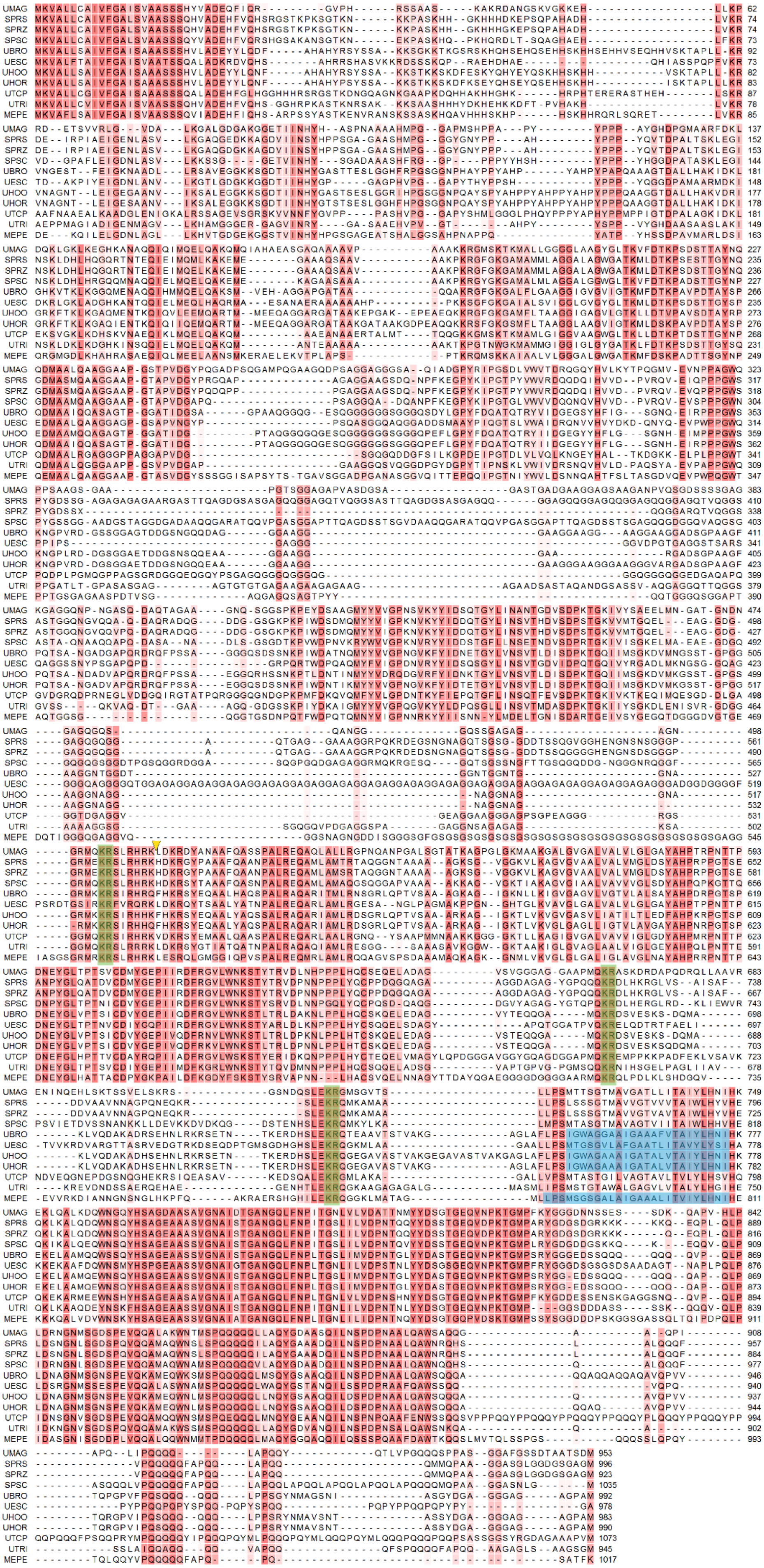

Supplementary Fig.7 Alignment of Stp6 orthologues from 11 sequenced smut fungi. For genomes lacking annotation amino acid sequences were extracted with the program CLC benchtop by running tBlastn predictions of the genome sequence data using the respective *U. maydis* or the *U. hordei* (host: barley) protein as a reference. The alignments were generated with CLC benchtop. Red background colour indicates conservation between more than 50% of the sequences. The darker the colour the higher the conservation. MEPE: *Melanopsichium pennsylvanicum*; UESC: *Ustilago esculenta*; SPSC: *Sporisorium scitamineum*; SPRZ: *Sporisorium reilianum* f. sp. *zeae*; SPRS: *Sporisorium reilianum* f. sp. *sorghi*; UTCP: *Ustilago trichophora*; UTRI: *Ustilago tritici*; UMAG: *U. maydis*; UBRO: *Ustilago bromivora*; UHOR: *Ustilago hordei* (host: barley); UHOO: *Ustilago hordei* (host: oats). Transmembrane domains predicted by TMHMM Server, V2.0 are highlighted in blue. The triangle in *U. maydis* Stp6 marks the position where Stp6s terminates.The Stp6s sequence harbors an additional C-terminal valine that is not present in Stp6 due to splicing. Putative Kex2 cleavage sites are highlighted in green.

Supplementary Table 1: Strains used in the study.

**Name**	**Number**	**Genotype**	**Resistance[1]**	**Plasmid name**	**Comments**
SG200		*a1 mfa2 bW2 bE1*	P		Ref.[18]
SG200Δstp1	EDB26	*a1 mfa2 bW2 bE1 stp1::hyg:egfp*	P, HY	pKS6	This study
SG200Δstp1-c	EDB209	*a1 mfa2 bW2 bE1 stp1::hyg:egfp* $ip^R[P_{stp1}:stp1]ip^S$	P, HY, C	pKS43	This study
SG200Δstp2	EDB75	*a1 mfa2 bW2 bE1 stp2::hyg*	P, HY	pNL3	This study
SG200Δstp2-c	EDB207	*a1 mfa2 bW2 bE1 stp2::hyg* $ip^R\ [P_{stp2}:stp2]ip^S$	P, HY, C	pNL7	This study
SG200Δstp3	EDB76	*a1 mfa2 bW2 bE1 stp3::hyg*	P, HY	pNL2	This study
SG200Δstp3-c	EDB208	*a1 mfa2 bW2 bE stp3::hyg* $ip^R\ [P_{stp3}:stp3]ip^S$	P, HY, C	pNL8	This study
SG200Δstp4	EDB102	*a1 mfa2 bW2 bE1 stp4::hyg*	P, HY	pSW16	This study
SG200Δstp4-c	EDB210	*a1 mfa2 bW2 bE1 stp4::hyg* $ip^R\ [P_{stp4}:stp4]ip^S$	P, HY, C	pSW20	This study
SG200Δpep1		*a1 mfa2 bW2 bE1 pep1::hyg*	P, HY		Ref.[4]
SG200Δpep1-c		*a1 mfa2 bW2 bE1 pep1::hyg* $ip^R\ [P_{pep1}:pep1]ip^S$	P, HY, C		Ref.[4]
SG200Δstp5	EDB112	*a1 mfa2 bW2 bE1 stp5::hyg*	P, HY	pPH5	This study
SG200Δstp5-c	LSM107	*a1 mfa2 bW2 bE1 stp5::hyg* $ip^R\ [P_{stp5}:stp5]ip^S$	P, HY, C	pPH12	This study
SG200Δstp6	CG60	*a1 mfa2 bW2 bE1 stp6::hyg*	P, HY	pCG10	This study
SG200Δstp6-c	CG156	*a1 mfa2 bW2 bE1 stp6::hyg* $ip^R\ [P_{stp6}:stp6]ip^S$	P, HY, C	pCG16	This study

SG200Δ10030	EDB74	*a1 mfa2 bW2 bE1 UMAG_10030::hyg*	P, HY	pNL1	This study
SG200Δ11062	EDB18	*a1 mfa2 bW2 bE1, UMAG_11062::hyg*	P, HY	pLL28	This study
SG200AN1		*a1 mfa2 bW2 bE1* P_{UMAG_01779}*:3xegfp:NatR*	P, N		Ref.[17]
SG200AM1Δstp1	KS352	*a1 mfa2 bW2 bE1 stp1::hyg:egfp* $ip^{R}[P_{UMAG\_01779}:3xegfp]ip^{S}$	P, HY, C	pAM95	This study
SG200AN1Δstp2	NL25	*a1 mfa2 bW2 bE1* P_{UMAG_01779}*:3xegfp:NatR stp2::hyg*	P, HY, N	pNL3	This study
SG200AN1Δstp3	NL40	*a1 mfa2 bW2 bE1* P_{UMAG_01779}*:3xegfp:NatR stp3::hyg*	P, HY, N	pNL2	This study
SG200AN1Δstp4	LL376	*a1 mfa2 bW2 bE1* P_{UMAG_01779}*:3xegfp:NatR stp4::hyg*	P, HY, N	pSW16	This study
SG200AM1Δpep1	NL208	*a1 mfa2 bW2 bE1 pep1::hyg* $ip^{R}[P_{um01779}:3xegfp]ip^{S}$	P, HY, C	pAM95	This study
SG200AM1Δstp5	DA202	*a1 mfa2 bW2 bE1, stp5::hyg* ip^{R} $[P_{um01779}:3xegfp]ip^{S}$	P, HY, C	pAM95	This study
SG200AN1 Δstp6	CG136	*a1 mfa2 bW2 bE1* $P_{UMAG-01779}$*:3xegfp:NatR stp6::hyg*	P, HY, NA	pCG10	This study
SG200Δstp1-stp1-HA	KM511	*a1 mfa2 bW2 bE1 stp1::hyg:egfp* $ip^{R}[P_{stp1}:stp1:HA]ip^{S}$	P, HY, C	pKS136	This study
SG200Δstp2-stp2-HA	NL52	*a1 mfa2 bW2 bE1 stp2::hyg* $ip^{R}[P_{stp2}:stp2:HA]ip^{S}$	P, HY, C	pNL12	This study
SG200Δstp3-stp3-HA	NL58	*a1 mfa2 bW2 bE1 stp3::hyg* $ip^{R}[P_{stp3}:stp3:HA]ip^{S}$	P, HY, C	pNL14	This study
SG200Δstp4-stp4-HA	KM508	*a1 mfa2 bW2 bE1 stp4::hyg* $ip^{R}[P_{stp4}:stp4:HA]ip^{S}$	P, HY, C	pSW23	This study
SG200Δpep1-pep1-HA	KM503	*a1 mfa2 bW2 bE1 pep1::hyg* $ip^{R}[P_{pep1}:pep1:HA]ip^{S}$	P, HY, C	p123-Ppep1-pep1-HA	This study
SG200Δstp6-stp6-HA	CG188	*a1 mfa2 bW2 bE1 stp6::hyg* $ip^{R}[P_{stp6}:stp6:HA]ip^{S}$	P, HY, C	pCG20	This study

SG200mCherryHA	LL106	*a1 mfa2 bW2 bE1* $ip^{R}[P_{cmu1}:SP_{cmu1}:mcherry:biotag:HA]ip^{S}$	P, C		Ref.[73]
SG200Δstp5-HA-stp5	LSM122	*a1 mfa2 bW2 bE1 stp5::hyg* $ip^{R}[P_{stp5}:HA:stp5]ip^{S}$	P, HY, C	pLSM 55	This study
SG200Δstp6-stp6s	CG102	*a1 mfa2 bW2 bE1 stp6::hyg* $ip^{R}[P_{stp6}:stp6s]ip^{S}$	P, HY, C	pCG14	This study
SG200Δstp6-stp6cDNA	CG303	*a1 mfa2 bW2 bE1 stp6::hyg* $ip^{R}[P_{stp6}:cDNAstp6]ip^{S}$	P, HY, C	pCG26	This study
SG200Δstp6-stp6s-HA stp6-HA	CG480	*a1 mfa2 bW2 bE1 stp6::hyg* $ip^{R}[P_{stp6}:stp6s:HA\ stp6:HA]ip^{S}$	P, HY, C	pCG43	This study
SG200Δstp6-stp6s-HA stp6	CG341	*a1 mfa2 bW2 bE1 stp6::hyg* $ip^{R}[P_{stp6}:stp6s:HA\ stp6]ip^{S}$	P, HY, C	pCG30	This study
SG200Δpep1-pep1$\Delta_{27\text{-}42}$-HA	MM518	*a1 mfa2 bW2 bE1 pep1::hyg* $ip^{R}[P_{pep1}:pep1_{\Delta 27\text{-}42}HA]ip^{S}$	P, HY, C	pMM231	This study
AB33		*a2* P_{nar} *bW2 bE1*	P		Ref.[53]
AB33Potef-stp2-HA	NL166	*a2* P_{nar} *bW2 bE1* $ip^{R}[P_{otef}:stp2:HA]ip^{S}$ Two copies	P, C	pNL27	This study
AB33Potef-stp3-HA	NL169	*a2* P_{nar} *bW2 bE1* $ip^{R}[P_{otef}:stp3:HA]ip^{S}$ Two copies	P, C	pNL28	This study
AB33Potef-stp4-HA	NL171	*a2* P_{nar} *bW2 bE1* $ip^{R}[P_{otef}:stp4:HA]ip^{S}$ Two copies	P, C	pNL29	This study
SG200Δkex2	NR 130	*a1 mfa2 bW2 bE1 kex2::hyg*	P, HY		Ref.[74]
SG200Δkex2 Potef-stp1-HA	KS311	*a1 mfa2 bW2 bE1 kex2::hyg* $ip^{R}[P_{otef}:stp1:HA]ip^{S}$ Two copies	P, HY, C	pKS205	This study
SG200Δstp5 Potef-HA-stp5	LSM134	*a1 mfa2 bW2 bE1 stp5::hyg* $ip^{R}[P_{otef}:SP_{stp5}:HA:stp5]ip^{S}$ Two copies	P, HY, C	pLSM58	This study
SG200Δstp6s Potef-stp6s-HA	CG468	*a1 mfa2 bW2 bE1 stp6s::hyg* $ip^{R}[P_{otef}:stp6s:HA]ip^{S}$ Multiple copies	P, HY, C	pCG41	This study
SG200Δstp6 Potef-stp6cDNA-HA	CG448	*a1 mfa2 bW2 bE1 stp6::hyg* $ip^{R}[P_{otef}:stp6cDNA:HA]ip^{S}$ Multiple copies	P, HY, C	pCG40	This study

SG200mCherry$_{cyt}$		*a1 mfa2 bW2 bE1* $ip^{R}[P_{otef}:mcherry:HA]ip^{S}$	P, C		Ref.[4]
SG200Potef-cmu1-HA	AD341	*a1 mfa2 bW2 bE1* $ip^{R}[P_{otef}:cmu1:3xHA]ip^{S}$	P, C	p123-Potef-cmu1-3xHA	This study
SG200Potef-pit1-HA	SR121	*a1 mfa2 bW2 bE* $ip^{R}[P_{otef}:pit1:HA]ip^{S}$	P, C	pSR36	This study
SG200Δpit2-pit2-mCherryHA		*a1 mfa2 bW2 bE1 pit2::hyg* $ip^{R}[P_{pit2}:pit2:mcherry:HA]ip^{S}$	P, HY, C		Ref.[44]
SG200Δstp1-stp1-mCherryHA	KS457	*a1 mfa2 bW2 bE1 stp1::hyg:egfp* $ip^{R}[P_{stp1}:stp1:mcherry:HA]ip^{S}$	P, HY, C	pKS145	This study
SG200Δstp2-stp2-mCherryHA	NL49	*a1 mfa2 bW2 bE1 stp2::hyg* $ip^{R}[P_{stp2}:stp2:mcherry:HA]ip^{S}$	P, HY, C	pNL13	This study
SG200Δstp3-stp3-mCherryHA	NL74	*a1 mfa2 bW2 bE1 stp3::hyg* $ip^{R}[P_{stp3}:stp3:mcherry:HA]ip^{S}$	P, HY, C	pNL15	This study
SG200Δstp4-stp4-mCherryHA	LL370	*a1 mfa2 bW2 bE1 stp4::hyg* $ip^{R}[P_{stp4}:stp4:mcherry:HA]ip^{S}$	P, HY, C	pSW24	This study
SG200Δpep1-pep1-mCherryHA	DA46	*a1 mfa2 bW2 bE1 pep1::hyg* $ip^{R}[P_{pep1}:pep1:mcherry:HA]ip^{S}$	P, HY, C	pDA37	This study
SG200Δstp5-HAmCherry-stp5	DA 259(s) DA261 (d)	*a1 mfa2 bW2 bE1 stp5::hyg* $ip^{R}[P_{stp5}:SPstp5:HA:mcherry:stp5]ip^{S}$	P, HY, C	pDA111	This study
SG200Δstp6-stp6-mCherryHA	CG264	*a1 mfa2 bW2 bE1 stp6::hyg* $ip^{R}[P_{stp6}:stp6:mcherry:HA]ip^{S}$	P, HY, C	pCG25	This study
SG200stp1-HAYFP stp3-mCherryHA	DA205	*a1 mfa2 bW2 bE1 stp1:HA:YFP stp3:mcherry:HA*	P	pDA99, pDA30	This study

SG200stp1-YFP$_C$ stp3-YFP$_N$	DA99	*a1 mfa2 bW2 bE1 stp1:2xHA:YFP$_C$ stp3:V5:HA:YFP$_N$*	P	pDA56, pDA59, pDA42	This study
SG200stp1-YFP$_N$ stp3-YFP$_C$	DA107	*a1 mfa2 bW2 bE1 stp1:2xHA:YFP$_N$ stp3:V5:HA:YFP$_C$*	P	pDA58, pDA57, pDA42	This study
SG200stp1-YFP	KM465	*a1 mfa2 bW2 bE1 stp1:HA:YFP*	P	pDA41, pKM1	This study
SG200stp3-YFP	KM476	*a1 mfa2 bW2 bE1 stp3:HA:YFP*	P	pDA30, pKM3	This study
SG200stp1-YFP$_N$	DA184	*a1 mfa2 bW2 bE1 stp1:2xHA:YFP$_N$*	P	pDA57, pDA75	This study
SG200stp3-YFP$_N$	DA186	*a1 mfa2 bW2 bE1 stp3:V5:HA:YFP$_N$*	P	pDA59, pDA76	This study
SG200stp4-YFP$_N$ pep1-YFP$_C$	DA130	*a1 mfa2 bW2 bE1 stp4:myc:HA:YFP$_N$ pep1:Strep:HA:YFP$_C$*	P	pDA74, pDA73, pDA34	This study
SG200pep1-YFP$_C$	DA131	*a1 mfa2 bW2 bE1 pep1:strep:HA:YFP$_C$*	P	pDA73, pDA34	This study
SG200stp1-YFP$_N$ stp3-YFP$_C$	DA138	*a1 mfa2 bW2 bE1 ip^R[P$_{stp1}$:stp1:2xHA-YFP$_N$: Tnos + P$_{stp3}$:stp3:V5:HA: YFP$_C$:Tnos]ip^S* Single copy	P, C	pDA80	This study
SG200stp1-YFP$_N$ stp3-YFP$_C$	DA135	*a1 mfa2 bW2 bE1 ip^R[P$_{stp1}$:stp1:2xHA:YFP$_N$: Tnos + P$_{stp3}$:stp3: V5:HA: YFP$_C$-Tnos]ip^S* Two copies	P, C	pDA80	This study
SG200stp1-YFP$_N$ stp3- YFP$_C$	DA132	*a1 mfa2 bW2 bE1 ip^R[P$_{stp1}$:stp1:2xHA:YFP$_N$: Tnos + P$_{stp3}$:stp3:V5:HA: YFP$_C$:Tnos]ip^S* More than three copies	P, C	pDA80	This study
SG200stp1-YFP$_N$ stp3-YFP$_C$	DA136	*a1 mfa2 bW2 bE1 ip^R [P$_{stp1}$:stp1:2xHA:YFP$_N$:Tnos + P$_{stp3}$:stp3:V5:HA: YFP$_C$:Tnos]ip^S* More than five copies	P, C	pDA80	This study

SG200Δstp1-stp1-3HA	LL319	*a1 mfa2 bW2 bE1 stp1::hyg:egfp ip^{R}[P$_{stp1}$:stp1:3HA]ip^{S}*	P, HY, C	pLL194	This study
FB1		*a1 b1*			Ref.[75]
FB2		*a2 b2*			Ref.[75]
FB1Δstp1	KS153	*a2 b2 stp1::hyg:egfp*	HY	pKS6	This study
FB2Δstp1	KS150	*a2 b2 stp1::hyg:egfp*	HY	pKS6	This study
AB33 Pum05031-stp4-mcherry-HA-Tnos + Phsp70-stp1-HA-Tstp1 + Pactin-stp3-HA-Tstp3 + Pum02442-pep1-HA-Tpep1	DA386	*a2 Pnar-bW2 Pnar-bE ip^{R}[Pum05031:stp4: mcherry:HA:Tnos + PUMAG_03791:stp1:HA: Tstp1 + UMAG_11232 :stp3:HA: Tstp3 + P UMAG_02442 :pep1:HA: Tpep1]ip^{S} More than two copies*	P, C	pDA140	This study
AB33complex^{con}	DA437	*a2 Pnar:bW2 Pnar:bE ip^{R}[PUMAG_05031:stp4: mcherry:HA:Tnos + PUMAG_03791:stp1:HA: Tstp1 + PUMAG_11232:stp3:HA: Tum00715 + PUMAG_02442:pep1:HA: Tpep1]ip^{S}; [PUMAG_05031 :stp6:HA: Tstp6 + PUMAG_03791:stp2:HA :Tstp2 + PUMAG_02442 :SPstp5:HA:stp5: Tstp5] mig2-6*	P, C, G	pDA141	This study
AB33 stp2en stp5en stp6en	DA463	*a2 Pnar-bW2 Pnar-bE,ble stp2en ; stp5en ; stp6en*	P	pPH52	This study
AB33Δ6complex	NL285	*a2 Pnar-bW2 Pnar-bE,ble stp2en ; stp5en ; stp6en ; stp1en ; stp3en ; stp4en*		pDA155	This study
FB1Δstp1-stp1-HA	KM515	*a1 b1 stp1::hyg:egfp ip^{R}[P$_{stp1}$:stp1:3xHA]ip^{S}*	P, HY, C	pLL194	This study

FB2Δstp1-stp1-HA	KM496	*a2 b2*<br>*stp1::hyg:egfp*<br>*$ip^R[P_{stp1}:stp1:3xHA]ip^S$*	P, HY, C	pLL194	This study
FB1 Pcmu1-mCherryHA	FF 258	*a1 b1*<br>*$ip^R[P_{cmu1}:mcherry:HA]ip^S$*	C	pLL97	This study
FB2 Pcmu1-mCherryHA	FF 259	*a2 b2*<br>*$ip^R[P_{cmu1}:mcherry:HA]ip^S$*	C	pLL97	This study
SG200 Δstp4 Δpep1	DA224	*a1 mfa2 bW2 bE1, ble; stp4::hyg; pep1::neo*	P, H, G	pNL39	This study
SG200 Δstp4 Δpep1 stp1en stp3en	DA256	*a1 mfa2 bW2 bE1, ble; stp4::hyg; pep1::neo; stp1en; stp3en*	P, H, G	pDA103, pDA104, pDA107	This study
SG200 Δstp4 Δpep1 stp1en stp3en stp2en stp5en stp6en	DA444	*a1 mfa2 bW2 bE1, ble; stp4::hyg; pep1::neo; stp1en; stp3en; stp2en; stp5en; stp6en*	P, H, G	PH52	This study
SG200Δcomplex Cmu1-mCherry	DA453	*a1 mfa2 bW2 bE1, ble; stp4::hyg; pep1::neo; stp1en; stp3en; stp2en; stp5en; stp6en*<br>*$ip^R[Pcmu1:cmu1:mcherry:HA:Tnos]ip^S$*<br>*Single copy*	P, H, G, C	pDA123	This study
FB1Δpep1		*a1 b1*<br>*pep1::hyg*			Ref.[4]
FB2Δpep1		*a2 b2*<br>*pep1::hyg*			Ref.[4]
U.hordei 4875-4		*MAT1*			Ref.[67,76]
U. hordei 4875-5		*MAT2*			Ref.[76]

[1] phleomycin (P), hygromycin (HY), carboxin (C), geneticin (G), nourseothricin (NA).

Supplementary Table 2: Plasmids and gBlocks used in the study.

Name	Description	Reference/Construction
PCRII® TOPO®	Plasmid backbone for cloning of PCR products.	Invitrogen, Karlsruhe, Germany
p123	Contains the *gfp* gene under control of the *otef*-promoter and *nos* terminator as well as the *U. maydis* carboxin resistant *ip* allele (ip^R). p123 served as backbone to insert constructs ectopically into the *U. maydis ip* locus.	Ref.[77]
pUMa229	Containing *egfp* and hygromycin resistance cassette.	Kindly provided by M. Feldbrügge.
pCRII TOPOΔstp1<br><br>Alternative name: pKS6	PCRII® TOPO®-derived plasmid containing the *stp1* (*UMAG_02475*) deletion construct, which consist of a hygromycin resistance cassette flanked by the right border of *stp1* and a *stp1* promoter-*egfp* fusion as left border.	The left and right border of *stp1* were PCR amplified from SG200 gDNA with primers oKS9/oKS10 and oKS11/oKS12 and digested with SfiI. The SfiI fragment from pUMa229 containing *egfp* and the hygromycin resistance cassette, and the two borders were ligated and integrated into the PCRII® TOPO® vector.
p123-Pstp1-stp1<br><br>Alternative name: pKS43	The p123-derived plasmid contains the *stp1 g*ene under control of the *stp1* promoter and the *nos* terminator.	The promoter and gene sequence of *stp1* were PCR amplified from SG200 gDNA with primers oKS117/oKS50. The resulting PCR product was digested with HindIII/NotI and integrated into the p123 HindIII/NotI backbone.
pHwtFRT	Plasmid containing the hygromycin resistance cassette (Hyg^R).	Ref.[78]
pJET1 stuffer	Plasmid-derived from pJET1 (Fermentas, St. Leon-Rot). Contains a 0.6 kb stuffer sequence flanked by EcoRV sites to allow amplification in DH5α.	Kindly provided by K.O. Schink und M. Bölker, unpublished.
pJET1Δstp2<br><br>Alternative name: pNL3	pJET1-derived plasmid containing the *stp2* (*UMAG_10067*) deletion construct which consists of a hygromycin resistance cassette flanked by the left and right border of the *stp2* gene.	The left border and right border of *stp2* were PCR amplified from SG200 gDNA with primers oNL18/oNL19 and oNL20/oNL21. The hygromycin resistance cassette was obtained from SfiI digest of pHwtFRT. The three fragments were integrated into the EcoRV linearized pJET1 backbone via Gibson assembly.
p123-Pstp2-stp2<br><br>Alternative name: pNL7	This p123-derived plasmid contains the *stp2* gene under control of the *stp2* promoter and the *nos* terminator.	The promoter and gene sequence of *stp2* were PCR amplified from SG200 gDNA with primers oNL57/oNL58. The resulting

		PCR product was integrated into the p123 SphI/NotI backbone via Gibson assembly.
pJET1Δstp3<br><br>Alternative name: pNL2	pJET1-derived plasmid containing the *stp3* (*UMAG_00715*) deletion construct which consists of a hygromycin resistance cassette flanked by the left and right border of the *stp3* gene.	The left border and right border of *stp3* were PCR amplified from SG200 gDNA with primers oNL10/oNL11 and oNL12/oNL13. The hygromycin resistance cassette was obtained from SfiI digest of pHwtFRT. The three fragments were integrated into the EcoRV linearized pJET1 backbone via Gibson assembly.
p123-Pstp3-stp3<br><br>Alternative name: pNL8	This p123-derived plasmid contains the *stp3* gene under control of the *stp3* promoter and the *nos* terminator.	The promoter and gene sequence of *stp3* were PCR amplified from SG200 gDNA with primers oNL65/oNL66. The resulting PCR product was integrated into the p123 SphI/NotI backbone via Gibson assembly.
pJET1Δstp4<br><br>Alternative name: pSW16	pJET1-derived plasmid containing the *stp4* (*UMAG_12197*) deletion construct which consists of a hygromycin resistance cassette flanked by the left and right border of the *stp4* gene.	The left border and right border of *stp4* were PCR amplified from SG200 gDNA with primers oSW37/oSW38 and oSW39/oSW40. The hygromycin resistance cassette was obtained from SfiI digest of pHwtFRT. The three fragments were integrated into the EcoRV linearized pJET1 backbone via Gibson assembly.
p123-Pstp4-stp4<br><br>Alternative name: pSW20	This p123-derived plasmid contains the *stp4* gene under control of the *stp4* promoter and the *nos* terminator.	The promoter and gene sequence of *stp4* were PCR amplified from SG200 gDNA with primers oSW64/oSW65. The resulting PCR product was integrated into the p123 KpnI/NotI backbone via Gibson assembly.
pBS-hhn	Plasmid containing the hygromycin resistance cassette (Hyg^R).	Ref.[45]
pCRIITopoΔstp5<br><br>Alternative name: pPH5	pCRIITopo-derived plasmid containing a *stp5 (UMAG_04342)* deletion construct which consists of a hygromycin resistance cassette flanked by the left and right border of the *stp5* gene.	The left border and right border of *stp5* were PCR amplified from SG200 gDNA with primers oZA56/oZA55 and oZA57/oZA58. The hygromycin resistance cassette was excised as SfiI fragment from pBS-hhn. The three fragments were integrated into pCRIITopo via Topo cloning.
p123-Pstp5-stp5<br><br>Alternative name: pPH12	This p123-derived plasmid contains the *stp5* gene under control of the *stp5* promoter sequence, the downstream region of the *stp5* gene followed by the *nos* terminator.	The promoter, gene and region downstream of *stp5* were PCR amplified from SG200 gDNA with primers oPH82/oPH83. The resulting PCR product was integrated into the p123 HindIII/NotI backbone via Gibson assembly.
pJET1Δstp6<br><br>Alternative name: pCG10	pJET1-derived plasmid containing the *stp6 (UMAG_01695)* deletion construct which consists of a hygromycin resistance cassette flanked by the left and right border of the *stp6* gene.	The left border and right border of *stp6* were PCR amplified from SG200 gDNA with primers oCG35/oCG36 and oCG44/oCG45. The hygromycin resistance cassette was obtained from SfiI digest of pHwtFRT. The three fragments were integrated into the

		EcoRV linearized pJET1 backbone via Gibson assembly.
p123-Pstp6-stp6<br><br>Alternative name: pCG16	This p123-derived plasmid contains the *stp6* gene under control of the *stp6* promoter and the *nos* terminator.	The promoter and gene sequence of *stp6* were PCR amplified from SG200 gDNA with primers oCG84/oCG85. The resulting PCR product was integrated into the p123 Acc65I/NotI backbone via Gibson assembly.
pJET1ΔUMAG_10030<br><br>Alternative name: pNL1	pJET1-derived plasmid containing the *UMAG_10030* deletion construct which consists of a hygromycin resistance cassette flanked by the left and right border of the *UMAG_10030* gene.	The left border and right border of *UMAG_10030* were PCR amplified from SG200 gDNA with primers oNL2/oNL3 and oNL4/oNL5. The hygromycin resistance cassette was obtained from SfiI digest of pHwtFRT. The three fragments were integrated into the EcoRV linearized pJET1 backbone via Gibson assembly.
pRS426	Plasmid backbone for yeast drag and drop cloning.	Ref.[79]
pRS426-um11062-5UTR-SfiI-Hygro-SfiI-um11062-3UTR<br><br>Alternative name: pLL28	pRS426-derived plasmid containing the *UMAG_11062* deletion construct which consists of a hygromycin resistance cassette flanked by the left and right border of the *UMAG_11062* gene.	The left border and right border of *UMAG_11062* were PCR amplified from SG200 gDNA with primers oLL76/oLL77 and oLL78/oLL79. The hygromycin resistance cassette was obtained from SfiI digest of pHwtFRT. The pRS426 BamHI/KpnI backbone, both borders and the resistance cassette were assembled using yeast drag and drop cloning.
pAM95	p123-derived plasmid containing the AM1 appressorial marker construct.	Ref.[20]
p123-mCherry-HA	p123-mCherry was constructed by excision of the *gfp* coding region from p123 using NcoI and NotI and substitution by *mCherry*.	Ref.[4]
p123-Pstp1-stp1-HA<br><br>Alternative name: pKS136	p123-derived plasmid forthe expression of *stp1–HA* under control of the *stp1* promoter and the *nos* terminator.	The promoter and gene sequence of *stp1* were PCR amplified from SG200 gDNA with primers oKS153/oKS333. The resulting PCR product was digested with MluI /NotI and integrated into the p123 MluI /NotI backbone.
p123-Pstp2-stp2-HA<br><br>Alternative name: pNL12	p123-mCherry-HA-derived plasmid for the expression of *stp2–HA* under control of the *stp2* promoter and the *nos* terminator.	The promoter and gene sequence of *stp2* were PCR amplified from SG200 gDNA with primers oNL57/oNL74. The resulting PCR product was integrated into the p123-mCherry-HA SphI/XbaI backbone via Gibson assembly.

p123-Pstp3-stp3-HA<br><br>Alternative name: pNL14	p123-mCherry-HA-derived plasmid for the expression of *stp3–HA* under control of the *stp3* promoter and the *nos* terminator.	The promoter and gene sequence of *stp3* were PCR amplified from SG200 gDNA with primers oNL65/oNL72. The resulting PCR product was integrated into the p123-mCherry-HA backbone SphI/XbaI via Gibson assembly.
p123-Pstp4-stp4-HA<br><br>Alternative name: pSW23	p123-mCherry-HA-derived plasmid for the expression of *stp4–HA* under control of the *stp4* promoter and the *nos* terminator.	The promoter and gene sequence of *stp4* were PCR amplified from SG200 gDNA with primers oSW64/oSW76. The resulting PCR product was integrated into the p123-mCherry-HA backbone KpnI/XbaI via Gibson assembly.
p123-Ppep1-pep1-HA	p123_Pwt-pep1-mCherry-HA-derived plasmid for the expression of *pep1-HA* under control of the *pep1* promoter and the *nos* terminator.	Fragment containing HA was generated by hybridizing primers K597/K598.<br>The fragment was integrated into NheI/XbaI plasmid pDA37 via Gibson assembly.
p123-Pstp6-stp6-HA<br><br>Alternative name: pCG20	p123-derived plasmid for the expression of *Stp6-HA* under control of the native promoter and the *nos* terminator.	The promoter and gene sequence of *stp6* were PCR amplified from SG200 gDNA with primers oCG97/oCG101. The *nos* terminator was amplifier from p123 with oCG98/oCG102. The two fragments were integrated into EcoRI backbone of p123 via Gibson assembly.
p123-HA-stp5<br><br>Alternative name: pLS55	p123-stp5-derived plasmid for the expression of *HA-Stp5* under control of the native promoter carrying the signal peptide of Stp5.	To generate pHA-stp5, two PCR fragments were generated using primer pairs oLSM63/oLSM62 and oLSM65/oLSM64 and pPH12 as template. The PCR products were integrated in the NcoI/SacI backbone of pPH12 via Gibson assembly.
p123-Pstp6-stp6s<br><br>Alternative name: pCG14	p123-derived plasmid contains *stp6s* under control of the native promoter and terminator.	The promoter, gene and terminator of *stp6s* was PCR amplified from SG200 gDNA with primers oCG72/oCG73.The resulting fragment was integrated into the p123 EcoRV/Acc65I backbone via Gibson assembly.
p123-Pstp6-stp6cDNA<br><br>Alternative name: pCG26	This p123-derived plasmid contains the *stp6* cDNA under control of the *stp6* promoter and the native terminator.	The promoter of *stp6* was PCR amplified from SG200 gDNA with primers oCG84/oCG116. The terminator was amplified from SG200 gDNA with primers oCG119/oCG52. The *stp6* cDNA was amplified from cDNA reverse transcribed from RNA isolated 3 dpi with SG200 using primers oCG117/oCG118. The three resulting PCR products were integrated into the p123 EcoRV/Acc65I backbone via Gibson assembly.
p123-stp6s-HA stp6-HA<br><br>Alternative name: CG43	p123-derived plasmid for the expression of both *stp6s-HA* and s*tp6-HA* under control of the native promoter and the *nos* terminator.	The 3´portion of *stp6s* was amplified with oCG101/oCG125 from SG200 gDNA. The 3´part of *stp6* was amplified with oCG126/oCG102 from plasmid p123-Pstp6-stp6-HA. The two fragments were

		integrated in plasmid p123-stp6 via Gibson assembly.
p123-stp6s-HA stp6<br><br>Alternative name: pCG30	p123-derived plasmid for the expression of *stp6s-HA* under control of the native promoter and the *nos* terminator.	The 3´portion of *stp6s* was amplified with oCG101/oCG125 from SG200 gDNA. The 3´part of stp6 was amplifier with oCG126/oCG102 from plasmid p123-Pstp6-stp6. The two fragments were integrated in plasmid p123-stp6 via Gibson assembly.
p123-Ppep1-pep1-HA	p123-derived plasmid for the expression of *pep1-HA* under control of the *pep1* promoter and the *nos* terminator.	Ref.[4]
p123-Ppep1-pep1$_{\Delta 27\text{-}42}$-HA<br><br>Alternative name: pMM231	p123-derived plasmid for the expression of *pep1*$_{\Delta 27\text{-}42}$*-HA* under control of the *pep1* promoter and the *nos* terminator.	Generated by inverse PCR with primers oMM662/oMM663 on p123-Ppep1-pep1-HA as template.
p123-Potef-stp2-HA<br><br>Alternative name: pNL27	p123-mCherry-HA-derived plasmid for the expression of *stp2–HA* under control of the *otef* promoter and the *nos* terminator.	The promoter and gene sequence of *stp2* were PCR amplified from SG200 gDNA with primers oNL188/oNL74. The resulting PCR product was integrated into the p123-mCherry-HA Nhe1/XbaI backbone via Gibson assembly.
p123-Potef-stp3-HA<br><br>Alternative name: pNL28	p123-mCherry-HA-derived plasmid for the expression of *stp3–HA* under control of the *otef* promoter and the *nos* terminator.	The promoter and gene sequence of *stp3* were PCR amplified from SG200 gDNA with primers oNL189/oNL72. The resulting PCR product was integrated into the p123-mCherry-HA backbone NheI/XbaI via Gibson assembly.
p123-Potef-stp4-HA<br><br>Alternative name: pNL29	p123-mCherry-HA-derived plasmid for the expression of *stp4–HA* under control of the *otef* promoter and the *nos* terminator.	The promoter and gene sequence of *stp4* were PCR amplified from SG200 gDNA with primers oNL190/oNL191. The resulting PCR product was integrated into the p123-mCherry-HA backbone NheI/XbaI via Gibson assembly.
p123-Potef-stp1-HA<br><br>Alternative name: pKS205	p123-derived plasmid for the expression of *stp1–HA* under control of the *otef* promoter and the *nos* terminator.	The promoter and gene sequence of s*tp1* were PCR amplified from SG200 gDNA with primers oKS153/oKS224. The resulting PCR product was integrated into the p123 NcoI/NotI backbone.
p123-Potef-HA-stp5<br><br>Alternative name: pLSM58	p123-stp5-derived plasmid expressing *HA-stp5* fused to the signal peptide of Stp5 under the constitutive *otef* promoter.	To generate Potef-HA-stp5, two PCR fragments were amplified using primer pairs oLSM85/oLSM86 and oLSM83/oLSM84 and pHA-stp5 as template. The PCR products were integrated in the NdeI/BalI backbone of p123-stp5 via Gibson assembly.
p123-Potef-stp6s-HA<br><br>Alternative name: pCG41	p123-derived plasmid for the expression of *stp6s-HA* under control of the *otef* promoter and the *nos* terminator.	The *stp6s* was amplified with oCG140/oCG125 from pCG30. The *nos* terminator was amplified with oCG98/oCG102 from plasmid p123. The two fragments were integrated in the

		EcoRI backbone of plasmid p123 via Gibson assembly.
p123-Potef-stp6cDNA-HA<br><br>Alternative name: pCG40	p123-derived plasmid for the expression of *stp6-HA* under control of the *otef* promoter and the *nos* terminator.	The *stp6* was amplified with oCG139/oCG140 from pCG26. The *nos* terminator was amplified with oCG98/oCG102 from plasmid p123. The two fragments were integrated in the EcoRI backbone of plasmid p123 via Gibson assembly.
p123-Potef-cmu1-3xHA	p123-derived plasmid for the expression of c*mu1-3xHA* under control of the *otef* promoter and the *nos* terminator.	Kindly provided by A. Djamei, unpublished.
p123-Potef-pit1-HA<br><br>Alternative name:pSR36	p123-derived plasmid for the expression of *pit1-HA* under control of the *otef* promoter and the *nos* terminator.	The promoter and gene sequence of *pit1* were PCR amplified from SG200 gDNA with primers oSR17/oSR18. The resulting PCR product was digested with Nde1/ Asc1and integrated into the p123 Nde1/ Asc backbone.
p123-Pstp1-stp1-mCherry-HA<br><br>Alternative name: pKS145	p123-derived plasmid for the expression of *stp1-mCherry-HA* under control of the *stp1* promoter and the *nos* terminator.	The *stp1* gene including the promoter region was PCR amplified with primers oKS259/ oKS265, thereby introducing an upstream HindIII and a codon optimized downstream short linker sequence (GGTGGCGATCGAGCG) followed by a BspHI site. The derived HindIII/BspHI fragment was integrated into the HindIII/NcoI linearized backbone of p123-mCherry-HA.
p123-Pstp2-stp2-mCherry-HA<br><br>Alternative name: pNL13	p123-mCherry-HA-derived plasmid for the expression of *stp2–mCherry-HA* under control of the *stp2* promoter and the *nos* terminator.	The promoter and gene sequence of *stp2* were PCR amplified from SG200 gDNA with primers oNL57/oNL73. The resulting PCR product was integrated into the p123-mCherry-HA BmtI/XmaI backbone via Gibson assembly.
p123-Pstp3-stp3-mCherry-HA<br><br>Alternative name: pNL15	p123-mCherry-HA-derived plasmid for the expression of *stp3–mCherry-HA* under control of the *stp3* promoter and the *nos* terminator.	The promoter and gene sequence of *stp3* were PCR amplified from SG200 gDNA with primers oNL65/oNL71. The resulting PCR product was integrated into the p123-mCherry-HA BmtI/XmaI backbone via Gibson assembly.
p123-Pstp4-stp4-mCherry-HA<br><br>Alternative name: pSW24	p123-mCherry-HA-derived plasmid for the expression of *stp4–mCherry-HA* under control of the *stp4* promoter and the *nos* terminator.	The promoter and gene sequence of *stp4* were PCR amplified from SG200 gDNA with primers oSW64o/SW77. The resulting PCR product was integrated into the p123-mCherry-HA KpnI/XmaI backbone via Gibson assembly.
p123-Pwt-pep1-mCherry-HA<br>Alternative name: pDA37	p123-mCherry-HA-derived plasmid containing *pep1-mCherry-HA* under control of the *pep1* promoter and the *nos* terminator.	Promoter and gene sequence of *pep1* were amplifed by PCR with primers oDA165/oDA166 and SG200 gDNA as template and ligated after digestion with

		enzymes SphI/BmtI into SphI/BmtI digested vector p123-Potef-mCherry-HA.
p123-Pstp1-SPstp1-mCherry-HA<br><br>Alternative name: pDA85	*mCherry-HA* under control of signal peptide and promoter of *stp1*.	Fragment containing promoter and signal peptide of *stp1* were generated by PCR with primers oDA312/oDA313 and SG200 gDNA as template and integrated into HindIII/BmtI digested plasmid pDA54 via Gibson assembly.
p123-Pstp5-SPstp5-mCherry-HA-stp5<br><br>Alternative name: pDA111	p123-derived plasmid expressing *mCherry-HA-stp5* under the native *stp5* promoter and signal peptide and the *nos* terminator.	Promoter and signal peptide sequence of *stp5* were PCR amplified from SG200 gDNA with primers oDA398/oDA399.<br>The *mCherry* sequence was PCR amplified with primers oDA402/oDA403 from plasmid pDA85.<br>The gene sequence of *stp5* was PCR amplified from SG200 gDNA with primers oDA400/oDA401.<br>The 3 fragments were integrated into PvuII/Not digested vector p123 via Gibson assembly.
p123-stp6-mCherry-HA<br><br>Alternative name: pCG25	p123-derived plasmid for the expression of *stp6-mCherry-HA* under the native promoter and *nos* terminator.	The promoter and gene sequence of *stp6* were PCR amplified from SG200 gDNA with primers oCG114/oCG101. The mCherry-HA *nos* fragment was amplified with oCG115/oCG102 from plasmid p123-mCherry-HA. The two fragments were integrated into the EcoRI backbone of p123 via Gibson assembly.
p123-Pstp3-SigPstp3-mCherry-HA<br><br>Alternative name: pDA54	p123-derived plasmid containing *mCherry-HA* under control of signal peptide and promoter of *stp3* and the *nos* terminator.	Fragment containing 398 bp upstream *stp3* and signal petide *stp3*was generated by PCR with primers oDA207/oDA208 and SG200 gnDNA as template and integrated into SphI/BmtI digested plasmid pDA37 via Gibson assembly.
p123-Pstp4-SigPstp4-mCherry-HA<br><br>Alternative name: pDA86	p123-derived plasmid containing *mCherry-HA* under control of signal peptide and promoter of *stp4* and the *nos* terminator.	Fragment 1 containing 902 bp upstream *stp4* and signal peptide of *stp4* was generated by PCR with primers oDA321/oDA322 and SG200 gDNA as template and integrated into HindIII/BmtI digested plasmid pDA54 via Gibson assembly.
pJET1-stp3-mCherry-HA<br><br>Alternative name: pDA99	pJET1-derived plasmid containing 1002 bp upstream *stp3* stop codon fused to *mCherry-HA* followed by 1218 bp downstream of *stp3* containing mutated PAM site.	Fragement 1 containing 1002 bp upstream *stp3* stop codon and a linker was generated by PCR with primers oDA153/oDA347 and gDNA of strain DA183 (SG200 stp3-V5-HA-YFP_N) as template.<br>Fragement 2 containing *mCherry-HA* was generated by PCR with primers oDA348/oDA349 and plasmid pDA86 as template.<br>Fragement 3 containing 1217 bp downstream *stp3* including mutated PAM site was generated by PCR with primers

		oDA350/oDA351 and gDNA of strain DA183 (SG200 stp3-V5-HA-YFP$_N$) as template.<br>The 3 fragments were integrated into EcoRV linearized vector pJET1 via Gibson assembly.
pMS73	CRISPR/Cas9 vector for multiplexed genome editing. Expresses *U. maydis* codon-optimized Cas9 under the *hsp70* promoter, contains a short version of *U6* promoter.	Ref.[80]
gBlock L28	gBlock containing overlap to *PU6* promoter, guide sequence of *stp1*, sgRNA scaffold, *PolIII* terminator and an overlap to the *hsp70* promoter.	At IDT Integrated DNA Technologies ordered gBlock containing overlap to *PU6* promoter, guide sequence of *stp1*, sgRNA scaffold, *PolIII* terminator and an overlap to the *hsp70* promoter.
pMS73-PU6-stp3-guide + Phsp70-UmSpCas9<br><br>Alternative name: pDA30	pMS73-derived plasmid containing *stp3* guide sequence under control of the *U6* promoter and *UmSpCas9* under control the *hsp70* promoter.	Fragment containing an overlap to *PU6* promoter, *stp3* guide sequence, sgRNA scaffold, *PolIII* terminator and an overlap to the *hsp70* promoter was generated by PCR with primers oDA175/oDA176 and gBlock L28 (guide *stp1-Phsp70*) as template and integrated via Gibson assembly into Acc65I linearized vector pMS73.
pMS73-PU6-stp1-guide + Pleu-stp3-guide + Phsp70-UmSpCas9<br><br>Alternative name: pDA33	pMS73-derived plasmid containing *stp1* guide sequence under control of the *U6* promoter, *stp3* guide sequence under control of the *Leu* promoter and *UmSpCas9* under control of the *hsp70* promoter.	gBlock1 consits of an overlap to *PU6* promoter, *stp1* guide sequence,sgRNA scaffold, *PolIII* terminator and *stp3* guide under control of the *Leu* promoter.<br>gBlock 2 includes guide sequence of *stp3*, sgRNA scaffold, *PolIII* terminator and an overlap to the *hsp70* promoter.<br>Both gBlocks were ordered at IDT Integrated DNA Technologies and integrated via Gibson assembly into Acc65I linearized vector pMS73.
pMS73-PU6-stp1 (1_166)-guide + Pleu-stp3-guide + Phsp70-UmSpCas9<br><br>Alternative name: pDA42	pMS73-derived plasmid containing *stp1*(1_166) guide sequence under control of the *U6* promoter, *stp3* guide sequence under control of the *Leu* promoter and *UmSpCas9* under control of the *hsp70* promoter.	Fragment 1 containing an overlap to the *PU6* promoter, *stp1* (1_166)-guide sequence, sgRNA scaffold , *PolIII* terminator and guide sequence of *stp3* under control of the *Leu* promoter was generated by PCR with primers oDA186/oDA187 and pDA33 as template.<br>Fragment 2 containing guide sequence of *stp3*, sgRNA scaffold, *PolIII* terminator and an overlap to the *hsp70* promoter was ordered as gBlock at IDT Integrated DNA Technologies.<br>Both fragments were integrated via Gibson assembly into Acc65I linearized vector pMS73.

pJET1-stp1-HA<br><br>Alternative name: pDA26	pJET1-derived plasmid containing 1000 bp upstream *stp1* stop codon fused to *HA* followed by 1270 bp downstream of *stp1*containing mutated PAM site.	Fragment 1 containing 1000 bp upstream *stp1* stop codon was generated by PCR with primers oDA149/oDA150 and SG200 gDNA as template.<br>Fragment 2 containing 25 bp overlap to *stp1* 3`end, linker, *HA* tag, stop codon, NotI site, 247 bp downstream *stp1* stop codon, mutated PAM site of *stp1* and 25 bp downstream guide sequence as overlap was ordered as gBlock at IDT Integrated DNA Technologies.<br>Fragment 3 containing 1000 bp downstream guide sequence *stp1* was generated by PCR with primers oDA151/oDA152 and SG200 gDNA as template.<br>The 3 fragments were integrated into EcoRV linearized vector pJET11 via Gibson assembly.
pJET1-stp1 (1_166)-HA<br><br>Alternative name: pDA40	pJET1-derived plasmid containing 1000 bp upstream *stp1* stop codon fused to *HA* followed by 1270 bp downstream of *stp1*containing mutated PAM site.	Fragment 1 containing 1000 bp upstream *stp1* stop codon was generated by PCR with primers oDA149/oDA150 and SG200 gDNA as template.<br>Fragment 2 containing *HA* and 266 bp downstream *stp1* and mutated PAM site was generated by PCR with primers oDA184/oDA185 and SG200 gDNA as template.<br>Fragment 3 containing 1037 bp downstream PAM site *stp1* was generated by PCR with primers ODA183/ODA152 and pDA26 as template.<br>The 3 fragments were integrated into EcoRV linearized vector pJET1 via Gibson assembly.
pEX-A2-YFP_Um-c.o.<br><br>Alternative name: pDA55	pEX-A2-derived plasmid containing *U. maydis* codon optimized *YFP*.	At eurofins ordered gene syntheses contains *U. maydis* codon optimized *YFP* flanked by 5´restriction site BamHI and 3´restriction site NotI cloned into pEX-A2 backbone.
pJET1-stp1-2xHA-YFP_C<br><br>Alternative name: pDA56	pJET1-derived plasmid containing 1000 bp 3'end of *stp1* fused to *2xHA-*YFP_C followed by 1270p downstream of *stp1* containing mutated PAM site.	Fragment containing *HA-*YFP_C was generated by PCR with primers oDA231/oDA232 and pDA55 as template.<br>The fragment was integrated into AvrII linearized plasmid pDA40 via Gibson assembly.
pJET1-stp3-V5<br><br>Alternative name: pDA36	pJET1-derived plasmid containing 1001 bp upstream *stp3* stop codon fused to *V5* followed by 1218 bp downstream of *stp3* containing mutated PAM site.	Fragment 1 containing 1001 bp upstream *stp3* stop codon was generated by PCR with primers oDA153/oDA154 and SG200 gDNA as template.<br>Fragment 2 containing 26 bp overlap to *stp3* 3`end, linker, *V5* tag, stop codon, NotI site, 177 bp downstream *stp3* stop codon,

		stp3 guide sequence, mutated PAM site and 25 bp downstream PAM site as overlap was ordered as gBlock at IDT Integrated DNA Technologies.<br>Fragment 3 containing 1018 bp downstream guide sequence *stp3* was generated by PCR with primers oDA155/oDA156 and SG200 gDNA as template.<br>The 3 fragments were integrated into EcoRV linearized vector pJET1 via Gibson assembly.
pJET1-stp3-V5-HA-YFP$_N$<br><br>Alternative name: pDA59	pJET1-derived plasmid containing *stp3* fused to *V5-HA-YFP$_N$* followed by 1218 bp downstream of *stp3* containing mutated PAM site.	Fragment containing *HA-YFP$_N$* was generated by PCR with primers oDA237/oDA238 and pDA55 as template. The fragment was integrated into AvrII linearized plasmid pDA36 via Gibson assembly.
pJET1-stp1-2xHA-YFP$_N$<br><br>Alternative name: pDA57	pJET1-derived plasmid containing 1000 bp 3'end of *stp1* fused to *2xHA-YFP$_N$* followed by 1270 bp downstream of *stp1* containing mutated PAM site.	Fragment containing *HA-YFP$_N$* was generated by PCR with primers oDA233/oDA234 and pDA55 as template. The fragment was integrated into AvrII linearized plasmid pDA40 via Gibson assembly.
pJET1-stp3-V5-HA-YFP$_C$<br><br>Alternative name: pDA58	pJET1-derived plasmid containing *stp3* fused to *V5-HA-YFP$_C$* followed by 1218b p downstream of *pep1* containing mutated PAM site.	Fragment containing *HA-YFP$_C$* was generated by PCR with primers oDA235/oDA236 and pDA55 as template. The fragment was integrated into AvrII linearized vector pDA36 via Gibson assembly.
pMS73-PU6-stp1-guide + Phsp70-UmSpCas9<br><br>Alternative name: pDA29	pMS73-derived plasmid containing *stp1* guide sequence under control of the *U6* promoter and *UmSpCas9* under control the *hsp70* promoter.	gBlock L28 containing an overlap to *PU6* promoter, *stp1 (1_166)* guide sequence, sgRNA scaffold, *PolIII* terminator and an overlap to the *hsp70* promoter was ordered at IDT Integrated DNA Technologies and integrated via Gibson assembly into Acc65I linearized vector pMS73.
pMS73-PU6-stp1 (1_166)-guide + Phsp70-UmSpCas9<br><br>Alternative name: pDA41	pMS73-derived plasmid containing *stp1 (1_166)* guide sequence under control of the *U6* promoter and *UmSpCas9* under control the *hsp70* promoter.	Fragment containing an overlap to *PU6* promoter, *stp1 (1_166)* guide sequence, sgRNA scaffold, *PolIII* terminator and an overlap to the *hsp70* promoter was generated by PCR with primers oDA176/oDA186 and pDA29 as template and integrated via Gibson assembly into Acc65I linearized vector pMS73.
pJET1-stp1-HA-YFP<br><br>Alternative name: pKM1	pJET1-derived plasmid containing 1000 bp upstream *stp1* stop codon fused to *HA-YFP* followed by 1270 bp downstream of *stp1* containing mutated PAM site.	Fragment containing *HA-YFP* was generated by PCR with primers oK582/oDA232 and pDA55 as template. The fragment was integrated into AvrII linearized plasmid pDA40 via Gibson assembly.

pJET1-stp3-HA-YFP<br><br>Alternative name: pKM3	pJET1-derived plasmid containing 981 bp upstream *stp3* stop codon fused to *HA-YFP* followed by 1218 bp downstream of *stp3* containing mutated PAM site.	Fragment 1 containing 981 bp upstream stp3 stop codon was generated by PCR with primers oDA153/oDA154 and SG200 gDNA as template.<br>Fragment 2 containing *HA-YFP* was generated by PCR with primers oK585/oDA236 and pDA55 as template.<br>Fragment 3 containing overlap to YFP, stop codon, 189 bp downstream *stp3* stop codon, mutated PAM site of *stp3* and 25 bp downstream PAM sequence as overlap was generated by PCR with primers oK586/oDA180 and SG200 gDNA as template.<br>Fragment 4 containing 1018 bp downstream PAM sequence of *stp3* was generated by PCR with primers oDA155/oDA156 and SG200 gDNA as template.<br>The 4 fragments were integrated into EcoRV linearized vector pJET1 via Gibson assembly.
gBlock L32_2	gBlock containing *stp3* guide sequence, sgRNA scaffold, *PolIII* terminator and an overlap to the *hsp70* promoter.	At IDT Integrated DNA Technologies ordered gBlock containing *stp3* guide sequence, sgRNA scaffold, *PolIII* terminator and an overlap to the *hsp70* promoter.
pMS73-PU6-stp1-guide + Pleu-stp4-guide + Phsp70-UmSpCas9<br><br>Alternative name: pDA75	pMS73-derived plasmid containing *stp1*-guide under control of the *U6* promoter, *stp4* guide under control of the *Leu* promoter and *UmSpCas9* under control of the *hsp70* promoter.	Fragment 1 containing overlap to the *PU6* promoter, guide sequence of *stp1*, sgRNA scaffold, *PolIII* terminator, guide sequence of *stp4* under control of the *Leu* promoter was generated by PCR with primers oDA186/oDA301 and pDA42 as template.<br>Fragement 2 containing guide sequence of *stp4*, sgRNA scaffold, *PolIII* terminator and an overlap to the *hsp*70 promoter was generated by PCR with primers oDA302/oDA176 and gBlock L32_2 (guide stp3-Phsp70) as template.<br>Fragments were integrated via Gibson assembly into Acc65I linearized vector pMS73.
pMS73-PU6-stp4-guide + Pleu-stp3-guide + Phsp70-UmSpCas9<br><br>Alternative name: pDA76	pMS73-derived plasmid containing *stp4*-guide under control of the *U6* promoter, *stp3* guide under control of the *Leu* promoter and *UmSpCas9* under control of the *hsp70* promoter.	Fragement containing an overlap to the *PU6* promoter, guide sequence of *stp4*, sgRNA scaffold, *PolIII* terminator, guide sequence of *stp3* under control of the *Leu* promoter, sgRNA scaffold, *PolIII* terminator and an overlap to the *hsp70* promoter was generated by PCR with primers oDA299/oDA176 and pDA63 as template.

		The fragment was integrated into Acc65I linearized vector pMS73 via Gibson assembly.
pMS73-PU6-stp4-guide + Pleu-pep1-guide + Phsp70-UmSpCas9<br><br>Alternative name: pDA34	pMS73-derived plasmid containing *stp4* guide under control of the *U6* promoter, *pep1* guide under control of the *Leu* promoter and *UmSpCas9* under control of the *hsp70* promoter.	gBlock 1 contains an overlap to *PU6* promoter, guide sequence of *stp4*, sgRNA scaffold, *PolIII* terminator and guide sequence of *pep1* under control of the *Leu* promoter. gBlock 2 contains guide sequence of *pep1*, sgRNA scaffold, *PolIII* terminator and an overlap to the *hsp70* promoter.<br>gBlocks were orderd at IDT Integrated DNA Technologies and integrated via Gibson assembly into Acc65I linearized vector pMS73.
pJET1-pep1-strep<br><br>Alternative name: pDA28	pJET1-derived plasmid containing 1003 bp upstream *pep1*stop codon fused to *strep* followed by 1222 bp downstream of *pep1*containing mutated PAM site.	Fragment 1 containing 1003 bp upstream *pep1* stop codon was generated by PCR with primers oDA161/oDA162 and SG200 gDNA as template.<br>Fragment 2 containing 25 bp overlap to *pep1* 3`end, linker, *strep* tag, stop codon, NotI site, 190 bp downstream *pep1* stop codon, mutated PAM site of *pep1* and 25 bp downstream PAM sequence as overlap was ordered as gBlock at IDT Integrated DNA Technologies.<br>Fragment 3 containing 1029 bp downstream guide sequence of *pep1* was generated by PCR with primers oDA163/oDA164 and SG200 gDNA as template.<br>The 3 fragments were integrated into EcoRV linearized vector pJET1 via Gibson assembly.
pJET1-pep1-strep-HA-YFP_C<br><br>Alternative name: pDA73	pJET1-derived plasmid containing a 1003 bp fragment upstream *pep1* stop codon fused to *strep-HA-*YFP_C followed by 1222 bp downstream of *pep1* containing mutated PAM site.	Fragment 1 containing 1003 bp upstream *pep1* stop codon tagged with *strep-HA* was generated by PCR with primers oDA161/oDA292 and pDA28 as template.<br>Fragment 2 containing YFP_C was generated by PCR with primers oDA289/oDA293 and pDA55 as template.<br>Fragment 3 containing 1222 bp downstream of *pep1* including mutated PAM site was generated by PCR with primers oDA294/oDA164 and pDA28 as template.<br>The 3 fragments were integrated via Gibson assembly into EcoRV linearized vector pJET1.
pJET1-stp4-myc<br><br>Alternative name: pDA27	pJET1-derived plasmid containing 999 bp upstream *stp4* stop codon fused to *myc* followed by 1031 bp	Fragment 1 containing 999 bp upstream *stp4* stop codon was generated by PCR

	downstream of *stp4* containing mutated PAM site.	with primers oDA157/oDA158 and SG200 gDNA as template.<br>A 161 bp gBlock containing *myc*-tag and mutated PAM site was ordered at IDT Integrated DNA Technologies as fragment 2.<br>Fragment 3 containing 979 bp downstream guide sequence *stp4* was generated by PCR with primers oDA159/oDA160 and SG200 gDNA as template.<br>The 3 fragments were integrated into EcoRV linearized vector pJET1 via Gibson assembly.
pJET1-stp4-myc-HA-YFP_N<br><br>Alternative name: pDA74	pJET1-derived plasmid containing a 999 bp fragment upstream *stp4* stop codon fused to *myc-HA-*YFP_N followed by 1031 bp downstream of *stp4* containing mutated PAM site.	Fragment 1 containing 999 bp upstream *stp4* stop codon tagged with *myc-HA* was generated by PCR with primers oDA157/oDA288 and pDA27 as template.<br>Fragment 2 containing YFP_N was generated by PCR with primers oDA295/oDA296 and pDA55 as template.<br>Fragment 3 containing 1031 bp downstream of *stp4* including mutated PAM site was generated by PCR with primers oDA297/oDA160 and pDA27 as template.<br>The 3 fragments were integrated via Gibson assembly into EcoRV linearized vector pJET1.
p123-Pstp1-stp1-HA-HA-YFP_N-Tnos<br><br>Alternative name: pDA79	p123-derived plasmid containing *stp1-2xHA-*YFP_N under control of the *stp1* promoter and the *nos* terminator.	Fragment *stp1-2xHA-*YFP_N under control of the *stp1* promoter and the *nos* terminator was generated by PCR with primers oDA303/oDA304 and gDNA of strain DA107 (SG200 stp3-V5-HA-YFP_C stp1-HA-HA-YFP_N) as template and integrated into HindIII/NotI digested plasmid pDA37.
p123-Pstp1-stp1-HA-HA-YFP_N-Tnos + Pstp3-stp3-V5-HA-YFP_C-Tnos<br><br>Alternative name: pDA80	p123-derived plasmid containing *stp1-2xHA-*YFP_N under control of the *stp1* promoter and the *nos* terminator and *stp3-V5-HA-*YFP_C under control of the *stp3* promoter and the *nos* terminator.	Fragment 1 containing promoter and gene sequence of *stp3* tagged with *V5-HA-*YFP_C was generated by PCR with primers oDA305/oDA306 and gDNA of strain DA107 (SG200 stp3-V5-HA-YFP_C stp1-HA-HA-YFP_N) as template.<br>Fragment 2 containing the *nos* terminator was generated by PCR with primers oDA307/oDA308 and plasmid pDA37 as template.<br>The 2 fragments were integrated via Gibson assembly into HpaI linearized plasmid pDA79.
P123-Pstp1-stp1-3xHA<br><br>Alternative name:pLL194	p123-derived plasmid for the expression of *stp1–3xHA* under control of the *stp1* promoter and the *nos* terminator.	The promoter and gene sequence of *stp1* were PCR amplified from SG200 gDNA. The reverse primer contained *3xHA* to generate a fusion protein of Stp1-3xHA.

		The resulting PCR product was digested with HindIII/NotI and integrated into the p123 HindIII/NotI backbone.
p123-Potef-pep-HA<br><br>Alternative name: pNL31	p123-mCherry-HA-derived plasmid for the expression of *pep1–HA* under control of the *otef* promoter and the *nos* terminator.	The promoter and gene sequence of *pep1* were PCR amplified from SG200 gDNA with primers oNL192/oNL193. The resulting PCR product was integrated into the p123-mCherry-HA Nhe1/XbaI backbone via Gibson assembly.
p123_Phsp70-stp1-HA-Tnos, cbx<br><br>Alternative name: pDA127	p123-derived plasmid to overexpress *stp1-HA* under control of the *hsp70* promoter and the nos terminator.	From plasmid pKS205 the *otef* promoter was removed using enzymes SbfI/AvrII and replaced with the *hsp70* promoter generated via PCR with primers oDA451/oDA452 and gDNA of strain SG200 as template.<br>Both fragments were merged via Gibson assembly.
p123_Pactin-stp3-HA-Tnos, cbx<br><br>Alternative name: pDA128	p123-derived plasmid to overexpress *stp3-HA* under control of the *actin* promoter and the *nos* terminator.	From plasmid pNL28 the *otef* promoter was removed using enzymes SbfI/BmtI and replaced with the *actin* promoter generated via PCR with primers oDA453/oDA454 and gDNA of strain SG200 as template.<br>Both fragments were merged via Gibson assembly.
p123_Pum02442-pep1-HA-Tnos, cbx<br><br>Alternative name: pDA129	p123-derived plasmid to overexpress *pep1-HA* under control of the *UMAG_02442* promoter and the *nos* terminator.	From plasmid pNL_Potef-pep1-HA-Tnos the *otef* promoter was removed using enzymes SbfI/BmtI and replaced with the *UMAG_02442* promoter generated via PCR with primers oDA455/oDA456 and gDNA of strain SG200 as template.<br>Both fragments were merged via Gibson assembly.
p123_Pum05031-stp4-HA-Tnos, cbx<br><br>Alternative name: pDA130	p123-derived plasmid to overexpress *stp4-HA* under control of the *UMAG_05031* promoter and the *nos* terminator.	From plasmid pNL29 the *otef* promoter was removed using enzymes SbfI/BmtI and replaced with the *UMAG_05031* promoter generated via PCR with primers oDA457/oDA458 and gDNA of strain SG200 as template.<br>Both fragments were merged via Gibson assembly.
p123_Phsp70-stp1-HA-Tstp1, cbx<br><br>Alternative name: pDA133	p123-derived plasmid to overexpress *stp1-HA* under control of the *hsp70* promoter and its native terminator.	From plasmid pDA127 the *nos* terminator was removed using enzymes NotI/EcoRI and replaced with the *stp1* terminator generated via PCR with primers oDA480/oDA481 and gDNA of strain SG200 as template.<br>Both fragments were merged via Gibson assembly.
p123_Pactin-stp3-HA-Tstp3, cbx	p123-derived plasmid to overexpress *stp3-HA* under control of the *actin* promoter and its native terminator.	From plasmid pDA128 the *nos* terminator was removed using enzymes NotI/EcoRI and replaced with the *stp3* terminator

Alternative name: pDA134		generated via PCR with primers oDA482/oDA483 and gDNA of strain SG200 as template.<br>Both fragments were merged via Gibson assembly.
p123_Pum02442-pep1-HA-Tpep1, cbx<br><br>Alternative name: pDA135	p123-derived plasmid to overexpress *pep1-HA* under control of the *UMAG_02442* promoter and its native terminator.	From plasmid pDA129 the *nos* terminator was removed using enzymes NotI/EcoRI and replaced with the *pep1* terminator generated via PCR with primers oDA484/oDA485 and gDNA of strain SG200 as template.<br>Both fragments were merged via Gibson assembly.
p123_Pum05031-stp4-mcherry-HA-Tnos, cbx<br><br>Alternative name: pDA136	p123-derived plasmid to overexpress *stp4-mcherry-HA* under control of the *UMAG_05031* promoter and the *nos* terminator.	Fragment 1 containing the promoter *UMAG_05031* was generated via PCR with primers oDA457/oDA486 and plasmid pDA130 as template.<br>Fragment 2 containing *stp4-mcherry-HA-Tnos* was generated via PCR with primers oDA487/oDA491 and plasmid pSW24 as template.<br>Both fragments were merged via Gibson assembly to p123 backbone obtained after digest of plasmid pNL29 with enzymes SbfI/EcoRI.
p123_Pum05031-stp4-mcherry-HA-Tnos + Phsp70-stp1-HA-Tstp1 + Pactin-stp3-HA-Tstp3 + Pum02442-pep1-HA-Tpep1, cbx<br><br>Alternative name: pDA140	p123-derived plasmid for overexpression of *stp4-mcherry-HA* under control of the *um05031* promoter and the *nos* terminator, *stp1-HA* under control of the *hsp70* promoter and its native terminator, *stp3-HA* under control of the *actin* promoter and its native terminator and *pep1-HA* under control of the *UMAG_02442* promoter and its native terminator.	To obtain fragment 1 containing the p123 backbone and *stp4-mcherry-HA* under the control of the *UMAG_05031* promoter and the *nos* terminator plasmid pDA136 was linearized with SpeI.<br>Fragment 2 containing *stp1-HA* under control of the *hsp70* promoter and its native terminator was generated via PCR with primers oDA492/oDA493 and pDA133 as template.<br>Fragment 3 containing *stp3-HA* under the control of the *actin* promoter and its native terminator was generated via PCR with primers oDA494/oDA495 and pDA134 as template.<br>Fragment 4 containing *pep1-HA* under control of the *UMAG_02442* promoter and its native terminator was generated via PCR with primers oDA496/oDA497 and plasmid pDA135 as template.<br>All 4 fragments were merged with Gibson assembly.
p123_ p123_ Phsp70-stp2-HA-Tnos, cbx<br><br>Alternative name: pDA131	p123-derived plasmid to overexpress *stp2-HA* under control of the *hsp70* promoter and the *nos* terminator.	From plasmid pNL27 the *otef* promoter was removed using enzymes SbfI/AvrII and replaced with the *hsp70* promoter generated via PCR with primers oDA467/oDA468 and gDNA of strain SG200 as template.

		Both fragments were merged via Gibson assembly.
p123_Pum02442-SPstp5-HA-stp5-Tnos, cbx<br><br>Alternative name: pDA132	p123-derived plasmid to overexpress *HA-stp5* under control of the *UMAG_02442* promoter and the *nos* terminator.	Fragment 1 containing the *UMAG_02442* promoter was generated via PCR with primer oDA473/oDA474 and gDNA of strain SG200 as template.<br>Fragment 2 containing the signalpeptide of *stp5* fused to *HA-stp5* was generated via PCR with primers oDA475/oDA401 and gDNA of strain SG200 as template.<br>Both fragments were merged via Gibson assembly to p123 backbone with *nos* terminator obtained with digest of plasmid pDA111 with enzymes NdeI/NotI.
p123_Phsp70-stp2-HA-Tstp2, cbx<br><br>Alternative name: pDA137	p123-derived plasmid to overexpress *stp2-HA* under control of the *hsp70* promoter and its native terminator.	From plasmid pDA131 the *nos* terminator was removed using enzymes NotI/EcoRI and replaced with the *stp2* terminator generated via PCR with primers oDA498/oDA499 and gDNA of strain SG200 as template.<br>Both fragments were merged via Gibson assembly.
p123_Pum02442-SPstp5-HA-stp5-Tstp5, cbx<br><br>Alternative name: pDA138	p123-derived plasmid to overexpress *HA-stp5* under control of the *UMAG_02442* promoter and its native terminator.	From plasmid pDA132 the *nos* terminator was removed using enzymes NotI/EcoRI and replaced with the *stp5* terminator generated via PCR with primers oDA404/oDA405 and gDNA of strain SG200 as template.<br>Both fragments were merged via Gibson assembly.
Fragment Pum05031-stp6<br><br>Alternative name: F134	PCR product containing *stp6* under control of the *UMAG_05031* promoter.	Fragment obtained via Overlap-PCR with primers oDA469/oDA472 and PCR product 1 (PCR with primers oDA469/oDA470 and gDNA of SG200 as template) and PCR product 2 (PCR with primers oDA471/oDA472 and gDNA of SG200 as template) as templates.
p123_Pum05031-stp6-HA-Tstp6, neo<br><br>Alternative name: pDA139	p123-derived plasmid to overexpress *stp6-HA* under control of the *UMAG_05031* promoter and its native terminator.	Fragment containing *stp6-HA* overexpressed under control of the *UMAG_05031* promoter was generated via PCR with primers oDA500/oDA501 and F134 as template.<br>Fragment containing the *stp6* terminator was generated via PCR with primers oDA502/oDA503 and gDNA of strain SG200 as template.<br>With Gibson assembly both fragments were fused to a backbone derived after digestion of plasmid pCG48 with enzymes SbfI/ClaI.

p123_Ppst1-pst1-mcherry-HA-Tnos<br><br>Altenative name: pCG44	p123-derived plasmid containing *pst1-mcherry-HA* under control of its native promoter and the *nos* terminator.	Fragment 1 containing *Ppst1-pst1* was generated by PCR with primers oCG49/oCG147 and gDNA of strain SG200 as template.<br>Fragment 2 containing *mcherry-HA* was generated by PCR with primers oCG133/oCG148 and plasmid pCG25 as template.<br>Fragment 3 containing the nos terminator and the backbone of p123 was derived via digest of plasmid p123 with enzymes NotI and Acc65I.<br>Fragments were merged via Gibson assembly.
p123_Potef-pst1-mcherry-HA-Tnos, cbx<br><br>Altenative name: pCG45	p123-derived plasmid containing *pst1-mcherry-HA* under control of the *otef* promoter and the *nos* terminator.	Fragment 1 containing backbone of p123 and the *otef* promoter was derived via digest of plasmid p123 with enzyme EcoRI.<br>Fragment 2 containing *UMAG_12357-mcherry-HA-Tnos* was generated by PCR with primers oCG140/oCG102 and plasmid pCGG44 as template.<br>Both fragments were merged via Gibson assembly.
Potef-pst1-mcherry-HA-Tnos, neo<br><br>Alternative name: pCG48	Plasmid for integration into mig2-6-locus containing *pst1-mcherry-HA* under control of the *otef* promoter and *nos* terminator.	Fragment 1 containing *Potef-pst1-mcherry-HA-Tnos* was derived via digest of plasmid oCG45 with enzymes BsiWI and BglII.<br>Fragment 2 containing backround of p123 with ampicillin resistence was generated by PCR with primers oCG152/oCG195 and plasmid pCG45 as template.<br>Fragment 3 containing neomycin under control of the *otef* promoter and the *cyc1* terminator was cut from plasmid pUMa1057 with enzyme SfiI.<br>Fragment 4 containing *mig2-6* unter control of its native promoter and terminator was generated by PCR with primers oCG150/oCG196 and gDNA of strain SG200 as template.<br>All fragments were merged via Gibson assembly.
p123_Pum05031-stp6-HA-Tstp6 + Phsp70-stp2-HA-Tstp2 + Pum02442-SPstp5-HA-stp5-Tstp5, neo<br><br>Alternative name: pDA141	p123-derived plasmid containing *stp6-HA* under control of the *UMAG_05031* promoter, *stp2-HA* under control of the *hsp70* promoter and *HA-stp5* under control of the *UMAG_02442* promoter. All 3 each under their native terminator.	To obtain fragment 1 containing the backbone of pCG48 and *stp6-HA* under control of the *UMAG_05031* promoter and its native terminator plasmid pDA139 was linearized with SbfI.<br>Fragment 2 containing *stp2-HA* under control of the *hsp70* promoter and its native terminator was generated via PCR with primers oDA506/oDA507 and pDA137 as template.

		Fragment 3 containing *HA-stp5* under the control of the *UMAG_02442* promoter and its native terminator was generated via PCR with primers oDA508/oDA509 and pDA138 as template.<br>All 3 fragments were merged with Gibson assembly.
fDL15,16,17	synthetized gBlocks.	Ref.[3]
pMS73_PU6-stp2-guide (KO) + Pleu-stp5-guide (KO) + Pgly-stp6-guide (KO) + Phsp70-UmSpCas9<br><br>Alternative name: pPH52	pMS73-derived plasmid containing *stp2* guide sequence under control of the *U6* promoter, *stp5* guide sequence under control of the *leu* promoter, stp6 guide sequence under control of the *gly* promoter, and *UmSpCas9* under control the *hsp70* promoter.	Fragment 1 containing an overlap to *PU6* promoter, *stp2* guide sequence, sgRNA scaffold, *PolIII* terminator, *leu* promoter and *stp5* guide sequence was generated by PCR with primers oPH376/oPH377 and synthesized fDL15 as template.<br>Fragment 2 containing *stp5* guide sequence, sgRNA scaffold, *PolIII* terminator, *gly* promoter *stp6* guide sequence was generated by PCR with primers oPH378/oPH379 and synthesized fDL16 as template.<br>Fragment 2 containing *stp6* guide sequence, sgRNA scaffold, *PolIII* terminator and an overlap to the *hsp* promoter was generated by PCR with primers oPH380/oPH138 and synthesized fDL17 as template.<br>All three fragments were integrated via Gibson assembly into Acc65I linearized vector pMS73.
pMS73_PU6-stp1_2_0-guide_KO + Pleu-stp3_6_0-guide_KO + Phsp70-UmSpCas9 #5<br><br>Alternative name: pDA107	pMS73-derived plasmid containing *stp1* guide sequence under control of the *U6* promoter, *stp3* guide sequence under control of the *leu* promoter and *UmSpCas9* under control the *hsp70* promoter.	Fragment 1 containing an overlap to *PU6* promoter, *stp1* guide sequence, sgRNA scaffold, *PolIII* terminator, *leu* promoter and an overlap to the *stp3* guide sequence was generated by PCR with primers oDA375/oDA389 and plasmid pDA75 as template.<br>Fragment 2 containing *stp3* guide sequence, sgRNA scaffold, *PolIII* terminator and an overlap to the *hsp* promoter was generated by PCR with primers oDA390/oDA176 and gBlock L32_2 as template.<br>Both fragments were integrated via Gibson assembly into Acc65I linearized vector pMS73.
gBlock NL1	gBlock containing overlap to *PU6* promoter, guide sequence of *stp1*, sgRNA scaffold, *PolIII* terminator, *Leu TAA tRNA* promoter and an	At IDT Integrated DNA Technologies ordered gBlock.

	assembly overhang (*stp3* guide sequence)	
gBlock NL2	gBlock containing *stp3* guide sequence, sgRNA scaffold, *PolIII* terminator *Gly GCC tRNA* promoter and an assembly overhang (*stp4* guide sequence)	At IDT Integrated DNA Technologies ordered gBlock.
gBlock NL3	gBlock containing *stp4* guide sequence, sgRNA scaffold, *PolIII* terminator *Tyr GTA tRNA* promoter and an assembly overhang (*pep1* guide sequence)	At IDT Integrated DNA Technologies ordered gBlock.
gBlock NL4	gBlock containing *pep1* guide sequence, sgRNA scaffold, *PolIII* terminator and an overlap to the *hsp70* promoter.	At IDT Integrated DNA Technologies ordered gBlock.
pMS73_PU6-stp1-guide (KO) + Pleu-stp3-guide (KO) + Pgly-stp4-guide (KO) + Ptyr-pep1-guide (KO) + Phsp70-UmSpCas9<br><br>Alternative name: pNL26	pMS73-derived plasmid containing *stp1* guide sequence under control of the *U6* promoter, *stp3* guide sequence under control of the *leu* promoter, *stp4* guide sequence under control of the *gly* promoter, *pep1* under control of the *tyr* promoter and *UmSpCas9* under control the *hsp70* promoter.	gBlocks NL1, NL2, NL3 and NL4 were ordered at IDT Integrated DNA Technologies and integrated via Gibson assembly into Acc65I linearized vector pMS73.
pMS73_PU6-stp1_2_0-guide (KO) + Pleu-stp3_6_0-guide (KO) + Pgly-stp4-guide (KO) + Ptyr-pep1-guide (KO) + Phsp70-UmSpCas9<br><br>Alternative name: pDA155	pMS73-derived plasmid containing *stp1* guide sequence under control of the *U6* promoter, *stp3* guide sequence under control of the *leu* promoter, *stp4* guide sequence under control of the *gly* promoter, *pep1* under control of the *tyr* promoter and *UmSpCas9* under control the *hsp70* promoter.	Fragment 1 containing an overlap to *PU6* promoter, *stp1* guide sequence, sgRNA scaffold, *PolIII* terminator, *leu* promoter and *stp3* guide sequence was generated by PCR with primers oDA375/oDA389 and plasmid pDA107 as template. Fragment 2 containing *stp3* guide sequence, sgRNA scaffold, *PolIII* terminator, *gly* promoter stp4 guide sequence, again sgRNA scaffold, *PolIII* terminator, *tyr* promoter followed by *pep1* guide sequence and sgRNA scaffold, *PolIII* terminator and an overlap to the *hsp* promoter was generated by PCR with primers oDA556/oDA557 and plasmid pNL26 as template.<br>Both fragments were integrated via Gibson assembly into Acc65I linearized vector pMS73.
pLL97	p123–derived plasmid for the expression of *mCherry-Avitag-HA* under control of the *cmu1* promoter and the *nos* terminator.	Ref.[73]
pUMa1057	Containing geneticin resistance cassette.	Ref.[81]
pJet1_Δpep::Neo<br><br>Alternative name: pNL39	pJet1-derived plasmid containing the *pep1* deletion construct which consists of a geneticin resistance	The left border and right border of *pep1* were PCR amplified from SG200 gDNA with primers oNL240/oNL241 and oNL242/oNL243. The geneticin resistance

	cassette flanked by the left and right border of the *pep1* gene.	cassette was obtained from SfiI digest of pUMa1057. The three fragments were integrated into the EcoRV linearized pJET1 backbone via Gibson assembly.
pJET1_stp1-KO (Donor)<br><br>Alternative name: pDA103	pJET1-derived plasmid containing 555 bp upstream of *stp1* followed by 957 bp downstream of *stp1*.	Fragment upstream of *stp1* was generated by PCR with primers oDA367/oDA368 and gDNA of strain SG200. Fragment downstream of *stp1* was generated by PCR with primers oDA369/oDA370 and gDNA of strain SG200.<br>The 2 fragments were integrated via Gibson assembly into EcoRV linearized vector pJET1.
pJET1_stp3-KO (Donor)<br><br>Alternative name: pDA104	pJET1-derived plasmid containing 398 bp upstream of *stp3* followed by 207 bp downstream of *stp3*.	Fragment upstream of *stp3* was generated by PCR with primers oDA371/oDA372 and gDNA of strain SG200.<br>Fragment downstream of *stp3* was generated by PCR with primers oDA373/oDA374 and gDNA of strain SG200.<br>The 2 fragments were integrated via Gibson assembly into EcoRV linearized vector pJET1.
p123_Pwt-cmu1-mcherry-HA-Tnos, cbx<br><br>Alternative name: pDA123	p123-derived plasmid containing *cmu1-mcherry* under control of the native promoter and the *nos* terminator	Fragment 1 containing *cmu1* and its native promoter was generated by PCR with primers oDA443/oDA44 and gDNA of strain SG200 as template.<br>Fragment 2 containing *mcherry-HA-Tnos* was derived from digestion of pDA37 with enzymes BmtI/SphI.<br>Both fragments were merged with Gibson assembly.
pGADT7-Pep1	pGADT7-derived plasmid for the expression of *HA-pep1* fused to the Gal4AD under control of the *Adh1* promoter and terminator in *S. cerevisiae.*	Ref.[6]
pGADT7-Pep1$_{\Delta 27\text{-}42}$<br><br>Alternative name: pMM283	pGADT7-derived plasmid for the expression of *HA-pep1*$_{\Delta 27\text{-}42}$ fused to the Gal4AD under control of the *Adh1* promoter and terminator in *S. cerevisiae.*	Generated by inverse PCR with primers oMM662/oMM780 on pGADT7-Pep1 as template.
pGBKT7-POX12	pGBKT7-derived plasmid for the expression of *cMyc-ZmPOX12* fused to the Gal4BD under control of the *Adh1* promoter and terminator in *S. cerevisiae.*	Ref.[6]

Supplementary Table 3: Oligonucleotides used in the study.

Name	Sequence	Restriction site
oKS9	AACCAGACAAAGTATCAGTTACAGC	
oKS10	CACGGCCATCTAGGCCTCTGCTTTATTTTCTCGATAAAGTTG	SfiI
oKS11	TTTCTCGCAGCAATCGGTGCTGAG	
oKS12	GTGGGCCTGAGTGGCCAGCTCTGCTGTAAAGAATCACGAA	SfiI
oKS117	GTGAAGCTTATTCCAAGCACAAAGTCCACTGCAC	HindIII
oKS50	AGCGGCCGCCTAACGAGAAGGAGGAGGTGCCATGGTC	NotI
oNL18	CTCGAGTTTTTCAGCAAGATAATATTTTCAAACGCGGCTAACTC	
oNL19	AGAATAGGAACTTCTGGCCATCTAGGCCTGCAGCAGTGAGAAAGAC	
oNL20	AGTATAGGAACTTCTGGCCTGAGTGGCCACTTGATCTCCTGGCTTC	
oNL21	AGGAGATCTTCTAGAAAGATAATATTCTCTCGTGTCTGTTACTG	
oNL57	GAGCAGCTGAAGCTTGCATGCTGTGGCGGGCTTAGCGCC	
oNL58	ACGATCTGCAGCCGGGCGGCCGCCGAAGCCAGGAGATCAAG	
oNL10	CTCGAGTTTTTCAGCAAGATAATATTGATCAGGTACGCGACAAG	
oNL11	AGAATAGGAACTTCTGGCCATCTAGGCCGTGTGGCGATCGTACGTC	
oNL12	AGTATAGGAACTTCTGGCCTGAGTGGCCGGCGCAAAAAAGCCTTGG	
oNL13	AGGAGATCTTCTAGAAAGATAATATTTCACCGCGGAAGATTCAC	
oNL65	GAGCAGCTGAAGCTTGCATGCGGCCTTTCCAGGACTCAA	
oNL66	ACGATCTGCAGCCGGGCGGCCGCAAGGCTTTTTTGCGCCTT	
oSW37	CTCGAGTTTTTCAGCAAGATAATATTTGATCCACATCAGCAGTC	
oSW38	TAGAGAATAGGAACTTCTGGCCATCTAGGCCACACCGCTTGAACAAGT G	
oSW39	GAAAGTATAGGAACTTCTGGCCTGAGTGGCCCCAGCCTTCTTGGCATT C	
oSW40	AGGAGATCTTCTAGAAAGATAATATTACAGTGCAGACGACTTTG	
oSW64	GAGCTCGGTACCACAGCGTTGACGATATGG	

oSW65	AGCCGGGCGGCCGCTGGCGAATGCCAAGAAGG	
oZA56:	TAGCACGATGCGGTGACA	
oZA55:	TTCGGCCATCTAGGCCCGAGTGAAACAGTGCTGG	
oZA57:	CACGGCCTGAGTGGCCCTTACATGCTAGGCTACG	
oZA58:	GGAAAAAAAACCCCCCAG	
oPH82:	TATAGAACTCGAGCAGCTGAAGCTTGGTACTCAATCCGAGTAG	
oPH83:	CGATCTGCAGCCGGGCGGCCGCTCTTACCTGGCTCTTTCG	
oCG35	TGGCTCGAGTTTTTCAGCAAGATAATATTGTCCCTCCTTCACACTTG	SspI
oCG36	GCAATTGTCACGCCATGGTGGCCATCTAGGCCGACTGCAGCTGGAGA ATC	
oCG44	GTGCGGCCGCATTAATAGGCCTGAGTGGCCTGGTATCGTAATTGGCAA C	NotI, SfiI
oCG45	GTAGGAGATCTTCTAGAAAGATAATATTGAGGTGCAAGAACTTAGC	SspI
oCG84	TGCCTGCAGGTCGAAATTCGAGCTCGGTACCGCGCATGGAGGATCAG AAC	Acc65I
oGC85	TTGAACGATCTGCAGCCGGGCGGCCGCATGCTCTGCCTGGCTAGTTG	NotI
oNL2	CTCGAGTTTTTCAGCAAGATAATATTGCGGACGCACAAGAATAC	
oNL3	AGAATAGGAACTTCTGGCCATCTAGGCCCTTGAACAGATGTCAGGC	
oNL4	AGTATAGGAACTTCTGGCCTGAGTGGCCTTGTCATGCGGACATACC	
oNL5	AGGAGATCTTCTAGAAAGATAATATTGAGGAGCTTGGAGAACTG	
oLL76	TTTAAAGCTACCGACGTGCC	
oLL77	TTTCTGGCTCGCAGCTTCG	
oLL78	TGCATACTCCCAACTACTCC	
oLL79	CAGGGAAAGGGTAGATGCC	
stp1HA-rev	GTGTACGCGGCCGCCTAGGCGTAGTCGGGGACGTCGTAGGGGTAACG AGAAGGAGGAGGTGCCATGGTC	NotI
oNL74	TCGGGCACGTCGTAGGGGTATCTAGAGACGTGTGCAACATTGCC	
oNL72	TCGGGCACGTCGTAGGGGTATCTAGAAGCAGAAGTGCTGTCGAT	

oSW76	GGGGTATCTAGAGTGGTGTACTTGGGACCAG	
K597	CGGTAGCATGTTTGGCATGTCTAGATACCCCTACGACGTGCCCGACTA CGCC	
K598	GGCGTAGTCGGGCACGTCGTAGGGGTATCTAGACATGCCAAACATGC TACCG	
oCG97	GGCGTAGTCGGGCACGTCGTAGGGGTAGGCAATGCTGCGAATCTTCAT GTCAGATGTCGCTGCTGTG	
oCG98	TACCCCTACGACGTGCCCGACTACGCCTGAGCGGCCGCCCGGCTGCAG ATCGTTCAAACA	NotI
oCG101	CTCAGATTGCAGACGGACCTTAC	
oCG102	CCGGCAGATCTGATATCATC	
oLSM63	CATGCAACGCAGTAGAGG	
oLSM62	ATAGTCGGGGACGTCGTAGGGATAAGCTTGGGCCGTGAAGG	
oLSM65	TACGACGTCCCCGACTATGCCGCGCCTGCCTCGGCCAAAGATGAATTG TTA	
oLSM64	CGCACCATCGGGAGCAGTAACAC	
oCG47	CCGATCATCCGAGATTTC	
oCG57	ATGAAGGTTGCCCTGCTC	
oCG62	TCACATGTCAGATGTCGC	
oCG63	GCGCTCCTATGAGCCATC	
oCG65	TGTCCAATCCGGTGACAG	
oCG71	CGTCGACCTGAACCATCCTC	
AP	GGCCACGCGTCGACTAGTACTTTTTTTTTTTTTTTTT	MluI, SpeI ,SalI, NotI
AUAP	GGCCACGCGTCGACTAGTAC	
oCG72	GCCTGCAGGTCGAAATTCGAGCTCGGTACCTGGAGACCTTGGAAAGG G	Acc65I
oCG73	CTATAGGGAGACCGGCAGATCTGATATCTCCATTGGCACCAGTATC	EcoRV
oCG116	ATGGCACAGAGCAGGGCAACCTTCATGACTGCAGCTGGAGAATC	
oCG119	CACAGCAGCGACATCTGACATGTGATGGTATCGTAATTGGCAAC	
oCG52	TAGGGAGACCGGCAGATCTGATATCGAGGTGCAAGAACTTAGC	

oCG117	ACTGAATGATTCTCCAGCTGCAGTCATGAAGGTTGCCCTGCTC	
oCG118	CTGGCTAGTTGCCAATTACGATACCATCACATGTCAGATGTCGC	
oCG125	GGCACGTCGTAGGGGTAGGCAATGCTGCGAATCTTTACCTTGCGGTGG CGAAGGG	
oCG126	TCGCAGCATTGCCTACCCCTACGACGTGCCCGACTACGCCTGACTTTC CTTTTGCCCC	
oMM662	CCTCTGGCCAGCACGTTTTACTG	
oMM663	GGCATGGACGGGAACGGTGCTGCCC	
oNL188	GGGGGCCCGCGCCTAGGGCGGCTAGCATGATGTTGCCCTTCCAA	
oNL189	GGGGGCCCGCGCCTAGGGCGGCTAGCATGCAGCTAAATCGTACC	
oNL190	GGGGGCCCGCGCCTAGGGCGGCTAGCATGCATCGACCAACTAGC	
oNL191	TCGGGCACGTCGTAGGGGTATCTAGAGTGGTGTACTTGGGACCA	
oKS153	CACTCATGAGAGCCGTGCTCTCGCTCAAC	BspHI
oKS224	AGCGGCCGCCTAACGAGAAGGAGGAGGTGCCATGGTC	NotI
oLSM85:	CTATGCGGCATCAGAGCAG	
oLSM86:	GCATCTAGACTTAGGCCTGGATCCCGTGGATGATG	
oLSM83:	CCAGGCCTAAGTCTAGATGCGAGTCGGGGCATTG	
oLSM84:	CTCCGACTACACTCGAGATG	
oCG140	ATCCACGGGATCCCCCGGGCTGCAGGAATTCATGAAGGTTGCCCTGCT C	EcoRI
oCG125	GGCACGTCGTAGGGGTAGGCAATGCTGCGAATCTTTACCTTGCGGTGG CGAAGGG	
oCG139	GGCGTAGTCGGGCACGTCGTAGGGGTACATGTCAGATGTCGCTGCT	
oSR17	CGCGGCGCGCCTCAGGCATAGTCGGGGACGTCGTAGGGATAGCCGCC CGACATGTATGGGGCCGG	
oSR18	TATACATATGGAGCGTCACGATGG	
oKS259	CACGTGAAGCTTATTCCAAGCACAAAGTCCACTGCACG	HindIII
oKS265	TCTTCATGACGGTGGCGATCGAGCGACGAGAAGGAGGAGGTGCCATG GTCATGTTAGTC	BspHI
oNL73	TCCTCGCCCTTGCTCACCATCCATGGGACGTGTGCAACATTGCC	

oNL71	TCCTCGCCCTTGCTCACCATGCTAGCAGCAGAAGTGCTGTCGAT	
oSW77	GGGCCCCCCGGGGTGGTGTACTTGGGACCAG	
oDA165	GTGCATGCCAGGGCAGCTCAGAGTGTG	SphI
oDA166	GGGCTAGCCATGCCAAACATGCTACCG	BmtI
oDA312	CACTATAGAACTCGAGCAGCTGAAGCTTATTCCAAGCACAAAGTCCAC TGCAC	HindIII
oDA313	CCTCCTCGCCCTTGCTCACCATGGATCCGGCCTGAACGGCGACAAGTA TCGGC	BamHI
oDA398	GGTGACACTATAGAACTCGAGCAGCATTCCCGGATCCAGGTCGAGAG AAG	
oDA399	GTTATCCTCCTCGCCCTTGCTCACCATACTCCCACTGGCGTAGTCGGGC ACGTCGTAGGGGTATCCGCTACCAGCTTGGGCCGTGAAGGCTAAG	
oDA402	CGTGCCCGACTACGCCAGTGGGAGTATGGTGAGCAAGGGCGAG	
oDA403	GGCCGAGGCAGGCGCGGCAATGCTGCGAATCTTCTTGTACAGCTCGTC CATGCC	
oDA400	GTACAAGAAGATTCGCAGCATTGCCGCGCCTGCCTCGGCCAAAGATG	
oDA401	GAACGATCTGCAGCCGGGCGGCCGCTTAACCGATACCGTTAAATGTCA TGGTAGCAGCAGC	NotI
oCG114	CCCTTGCTCACCATGCTAGCCATGTCAGATGTCGCTGCTGTGTC	BmtI
oCG115	GACACAGCAGCGACATCTGACATGGCTAGCATGGTGAGCAAGGG	BmtI
oDA207	CTGCATGCGGCCTTTCCAGGACTCAAAC	SphI
oDA208	CTGCTAGCAGCAGAGACGCGTGAGACG	BmtI
oDA321	GGTGACACTATAGAACTCGAGCAGCTGAAGCTTCCTTTGATCCACATC AGCAGTCGACTCGCAC	HindIII
oDA322	CCTCCTCGCCCTTGCTCACCATGGATCCCGCTCGAACGCTCATCACCGT TCC	BamHI
oDA153	GATGGCTCGAGTTTTTCAGCAAGATAATATTGGACGTACGATCGCCAC ACG	SspI
oDA347	CCTCCTCGCCCTTGCTCACCATGGCAATGCTGCGAATCTTAGCAG	
oDA348	CTGCTAAGATTCGCAGCATTGCCATGGTGAGCAAGGGCGAGGAGG	
oDA349	GGCTTTTTTGCGCCTTGCGGCCGCTTAGGCGTAGTCGGGCACGTCGTA GGGGTATCTAGACTTGTACAGCTCGTCCATGCCGC	NotI, XbaI
oDA350	CTACGACGTGCCCGACTACGCCTAAGCGGCCGCAAGGCGCAAAAAAG CC	NotI
oDA351	GTAGGAGATCTTCTAGAAAGATAATATTCCCAGATGACGTTCCTGAGG ATCCTCCTGCCC	SspI

oDA175	CAAAATTCCATTCTACAACGGCCGCTGAAAATTAGCAGAGGTTTTAGA GCTAGAAATAGC	
oDA176	GGCGTTCGACTCTTGGCAGGTAC	
oLL182	TTCCAGATTACGCT**GC**GGACGAGCTGTACAAGTACCCAT	
oLL183	CATCGTATGGGTAGTCCATGCCGCCAGCGTAAT	
oDA186	GTAGCAGTCTGTCAGCATTCAAAATTCCATTCTACAACGGAGTATGTG CAATGTAGCCAGTTTTAGAGCTAGAAATAG	
oDA187	CTCTGCTAATTTTCAGCGGCCTTGACCAGACGGGATTCG	
oDA149	GATGGCTCGAGTTTTTCAGCAAGATAATATTAGGTGTGCCCGGGGGTA TGTC	SspI
oDA150	ACGAGAAGGAGGAGGTGCCATGGTCATGTTAGTCTCGATCATCTT	
oDA151	CAAACACGACCTTTTGAGGTCTAGACTGAAACGTTGGTCTGAGCC	
oDA152	GTAGGAGATCTTCTAGAAAGATAATATTCCGTTGCCAGCGGG	SspI
oDA184	GACCATGGCACCTCCTCCTTCTCGTAAGATTCGCAGCATTGCCTACCC CTACGACGTGCCCGACTACGCCTAGGCGGCCGCAGCTCTGCTGTAAAG AATCACG	NotI
oDA185	CTCTCAAACGGCTTTCCAGGCTTCGTCTTGGCTACATTGCACATACTCG ACCAATTCGATTCATTGTTTCGGCCAGCGCCTTCTCAGTGACGCTGG	
oDA183	CGAAGCCTGGAAAGCCGTTTGAGAG	
oDA231	CTACGACGTGCCCGACTACGCCGGTAGCGGATACCCATACGATGTTCC AGATTACGCTAGTGGGAGTGATAAGCAGAAGAACGGAATC	
oDA232	CAGCAGAGCTGCGGCCGCCTAGCTACTTGTACAGTTCGTCCATACCAA GCGTGATACC	NotI
oDA153	GATGGCTCGAGTTTTTCAGCAAGATAATATTGGACGTACGATCGCCAC ACG	SspI
oDA154	AGCAGAAGTGCTGTCGATCGCAGCATCGTCGACG	
oDA155	TGTGGGCAGATGCTACGAAGTAGGTGCAAC	
oDA156	GTAGGAGATCTTCTAGAAAGATAATATTCCCAGATGACGTTCCTGAGG	SspI
oDA237	CTCCTCGGCCTCGACTCGACCGGTAGCGGATACCCATACGATGTTCCA GATTACGCTAGTGGGAGTATGGTCAGCAAAGGCGAAG	
oDA238	GCGCCTTGCGGCCGCCTAGCTAGGCCATGATGTAGACGTTGTGCGAGT TGTAGTTG	NotI
oDA233	CGACGTGCCCGACTACGCCGGTAGCGGATACCCATACGATGTTCCAGA TTACGCTAGTGGGAGTATGGTCAGCAAAGGCGAAG	
oDA234	CAGCAGAGCTGCGGCCGCCTAGCTAGGCCATGATGTAGACGTTGTGC GAG	NotI
oDA235	CCTCGGCCTCGACTCGACCGGTAGCGGATACCCATACGATGTTCCAGA TTACGCTAGTGGGAGTGATAAGCAGAAGAACGGAATC	

oDA236	GCGCCTTGCGGCCGCCTAGCTACTTGTACAGTTCGTCCATACCAAGCGTGATACCG	NotI
oK582	CGACGTGCCCGACTACGCCGGTAGCGGAATGGTCAGCAAAGGCGAAG	
oK585	GCTGCGATCGACAGCACTTCTGCTAAGATTCGCAGCATTGCCTACCCATACGATGTTCCAGATTACGCTAGTGGGAGTATGGTCAGCAAAGGCGAAGAGC	
oK586	AACTGTACAAGTAGCTAGGCGGCCGCAAGGCGCAAAAAAGCCTTGG	
oDA180	ACCTACTTCGTAGCATCTGCCCACAGCGCTCTGCTAATTTTCAGCGGCTGCCTCAC	
oDA301	GATTATGGCGAATGCCAAGACTTGACCAGACGGGATTCG	
oDA302	TCTTGGCATTCGCCATAATCGTTTTAGAGCTAGAAATAGCAAG	
oDA299	GTAGCAGTCTGTCAGCATTCAAAATTCCATTCTACAACGTCTTGGCATTCGCCATAATCGTTTTAGAGCTAGAAATAG	
oDA161	GATGGCTCGAGTTTTTCAGCAAGATAATATTGCTTGTGGCTCACAAACGTC	SspI
oDA162	GCGACCAGGCAATGCTGCGAATCTTCATGCCAAACATGCTACCG	
oDA163	CCGTGGACGAAAGAGGTGATTGTTAAAGCC	
oDA164	GTAGGAGATCTTCTAGAAAGATAATATTGGCAGTCACACACTACGCAGGACC	SspI
oDA292	CACTCCCACTAGCGTAATCTGGAACATCGTATGGGTATCCGCTACCCTTCTCGAACTGCGGGTGCGACCAGGCAATGCTGCGAATCTTCATG	
oDA289	CGATGTTCCAGATTACGCTAGTGGGAGTGATAAGCAGAAGAACGGAATC	
oDA293	CAACGACGTCGCAGCGGTGCGGCCGCCTACTTGTACAGTTCGTCCATACC	NotI
oDA294	TTGGTATGGACGAACTGTACAAGTAGGCGGCCGCACCGCTGCGACGTCGTTGATG	NotI
oDA157	GATGGCTCGAGTTTTTCAGCAAGATAATATTGCAGATCGGATCTTTCTCAG	SspI
oDA158	GTGGTGTACTTGGGACCAGGCGGTTTCG	
oDA159	CTTCGAGTTGCGCGTCCATACCACCACACC	
oDA160	GTAGGAGATCTTCTAGAAAGATAATATTCGTGAATCAAGGCTGCTACCGCG	SspI
oDA288	CTCCCACTAGCGTAATCTGGAACATCGTATGGGTATCCGCTACCGAGGTCCTCCTCCGAGATCAGCTTCTGTTCGG	
oDA295	CGATGTTCCAGATTACGCTAGTGGGAGTATGGTCAGCAAAGGCGAAGAGC	
oDA296	GGGGGGAAGCGGGCGGCCGCCTAGGCCATGATGTAGACGTTGTGCGAG	NotI
oDA297	CATCATGGCCTAGGCGGCCGCCCGCTTCCCCCCTCCCGTCGCTCGTCCAGAC	

oDA303	GACACTATAGAACTCGAGCAGCTGATCTAGAATTCCAAGCACAAAGT CCACTGCACGACTCG	XbaI
oDA304	GAACGATCTGCAGCCGGGCGGCCGCCTAGGCCATGATGTAGACGTTG TGCGAGTTGTAG	NotI
oDA305	GTCGTATTAATTTCGATAAGCCAGGTTGGATCCGGCCTTTCCAGGACT CAAACATAATTTCATCTTGC	
oDA306	GAACGATCTGCAGCCGGGCGGCCGCACTAGTCTACTTGTACAGTTCGT CCATACCAAGCGTG	NotI
oDA307	CACGCTTGGTATGGACGAACTGTACAAGTAGACTAGTGCGGCCGCCC GGCTGCAGATCGTTC	NotI
oDA308	GGCCGATTCATTAATGCAGGTTCTCATGTTTGACAGCTTATCATCGGA TCTAGTAACATAGATGACACCGCGCGCG	
oNL192	GGGGGCCCGCGCCTAGGGCGGCTAGCATGATGACCACACTGGTG	
oNL193	TCGGGCACGTCGTAGGGGTATCTAGACATGCCAAACATGCTACC	
oDA451	GAGCAGCTGAAGCTTGCATGCCTGCATGCCAAGAGTCGAACGCCGAG	
oDA452	CACGGCTCTCATGCTAGCCGCCCTAGTGTGAAATCTAAAACTTTTGCT GTGGTGAAGGG	
oDA453	GAGCAGCTGAAGCTTGCATGCCTGCATGTCAATCAACTTTTCCAGGCC	
oDA454	GATCCAGGTACGATTTAGCTGCATGGATGACTGTGAACTAGGTTTTGG TG	
oDA455	CTGAAGCTTGCATGCCTGCAGCTGTCAAGCGTCAGTGTAGCTTG	
oDA456	GCACCAGTGTGGTCATCATGTTTGTTGGAGTGGTATATGAGTGACTAT GGGTG	
oDA457	GAGCAGCTGAAGCTTGCATGCCTGCAGATTGCTAGCTTTGACCAAGAT GGTTG	
oDA458	GTAGAGGCTAGTTGGTCGATGCATGCTTGACGTTGGTCGTGGGGG	
oDA480	CGACGTGCCCGACTACGCCTAAGCAGCTCTGCTGTAAAGAATCACG	
oDA481	GCAGATCTGATATCATCGATGAATTTTATCAACATTTCTCTCGTTGTCG	
oDA482	CGACGTGCCCGACTACGCCTAAGCAAGGCGCAAAAAAGCCTTGGTAA AGCTAG	
oDA483	CTATAGGGAGACCGGCAGATCTGATCATCTGCCCACACCGCTCTGC	
oDA484	CGACGTGCCCGACTACGCCTAAGCACCGCTGCGACGTCGTTGATGATG	
oDA485	GCAGATCTGATATCATCGATGAATTCTGCTTCGTATTTACCCCAGACT GGGTTG	EcoRI
oDA486	CGTAGAGGCTAGTTGGTCGATGCATCTTGACGTTGGTCGTGGGG	
oDA487	GTCACCCCCACGACCAACGTCAAGATGCATCGACCAACTAGCCTC	

oDA491	GCAGATCTGATATCATCGATGAATTACTAGTCTCATGTTTGACAGCTT ATC	SpeI
oDA492	CTAGATCCGATGATAAGCTGTCAAACATGAGACTAGTTGCCAAGAGTC GAACGCCGAGAAGG	SpeI
oDA493	GGACAACAACAAAGAGGCCTGGAAAAGTTGATTGACATTATCAACAT TTCTCTCGTTGTCGGCTCGACGCACTCGTTGTTGC	
oDA494	GTGCGTCGAGCCGACAACGAGAGAAATGTTGATAATGTCAATCAACT TTTCCAGGCCTCTTTG	
oDA495	CAAAACAAGCTACACTGACGCTTGACAGCCATCTGCCCACACCGCTCT G	
oDA496	CTGAAAATTAGCAGAGCGGTGTGGGCAGATGGCTGTCAAGCGTCAGT GTAG	
oDA497	GCAGATCTGATATCATCGATGAATTACTAGCTGCTTCGTATTTACCCC AGAC	
oDA467	CTCGAGCAGCTGAAGCTTGCATGCCTGCATGCCAAGAGTCGAACGCC GAG	
oDA468	GGAAGGGCAACATCATGCTAGCCGCCCTAGTGTGAAATCTAAAACTTT TGCTGTGGTGAAGGG	
oDA473	CATCAGAGCAGATTGTACTGAGAGTGCACCAGCTGTCAAGCGTCAGT GTAG	
oDA474	CAAGCAAAAGAGAAGCAATGCCCCGACTCGCATTTTGTTGGAGTGGT ATATGAGTGAC	
oDA475	ATGCGAGTCGGGGCATTGCTTCTCTTTTGCTTGGCATGCTTAGCCTTCA CGGCCCAAGCTTCTAGATACCCCTACGACGTGCCCGACTACGCCTCTA GAGCGCCTGCCTCGGCCAAAG	
oDA498	CGACGTGCCCGACTACGCCTAAGCATTACTTGATCTCCTGGCTTCGG	
oDA499	GACCGGCAGATCTGATATCATCGATGAATTTACACGTTCTGCAAGCGT AAC	
oDA404	CAACCAGCTAGTGGCACG	
oDA405	GAGATCAGGATGACGGGC	
oDA469	CATTTCGCCTTCTCGGCGTTCGACTCTTGGCAGATTGCTAGCTTTGACC AAGATGGTTG	
oDA472	CGTAGTCGGGCACGTCGTAGGGGTATCTAGACATGTCAGATGTCGCTG CTG	XbaI
oDA470	GACGATGGCACAGAGCAGGGCAACCTTCATCTTGACGTTGGTCGTGG GGG	
oDA471	CCACCAGTCACCCCCACGACCAACGTCAAGATGAAGGTTGCCCTGCTC TG	
oDA500	GAACTCGAGCAGCTGAAGCTTGCATGCCTGCAGATTGCTAGCTTTGAC CAAG	
oDA501	CTCTGCCTGGCTAGTTGCCAATTACGATACCATCAGGCGTAGTCGGGC ACGTCGTAGGGGTATCTAGAC	
oDA502	GATACCCCTACGACGTGCCCGACTACGCCTGATGGTATCGTAATTGGC AACTAG	
oDA503	CTATAGGGAGACCGGCAGATCTGATATCATCGCCTGCAGGAGGTGCA AGAACTTAGCTTTG	SbfI

oCG49	CAGGTCGAAATTCGAGCTCGGTACCATTCTGCTCTGGGGCTATC	Acc65I
oCG147	CCTCGCCCTTGCTCACCATGCTAGCTACCTTGCGGTGGCGAAGGG	
oCG133	GCTAGCATGGTGAGCAAGGGCGAGGAGGATAAC	
oCG148	GAACGATCTGCAGCCGGGCGGCCGCTCAGGCGTAGTCGGGCAC	NotI
oCG140	ATCCACGGGATCCCCCGGGCTGCAGGAATTCATGAAGGTTGCCCTGCT C	EcoRI
oCG102	CCGGCAGATCTGATATCATC	
oCG152	CGCAATTGTCACGCCATGGTGGCCATCTAGGCCTGAAAAAGGAAGAG TATGAG	SfiI
oCG195	ACATGAGAATTCATCGATGATATCAGATCTGCCGGTCTCCCTATAG	BglII
oCG150	CTGTAGGAGTGCGGCCGCATTAATAGGCCTGAGTGGCCCTATTGCATC GTAGGCATTGCG	SfiI
oCG196	CGCTCAACTCGTCTCGCTTTCGTACGTCGTGAGTACCACGCTGATG	BsiWI
oDA506	CATCAAAGCTAAGTTCTTGCACCTCCTGCATGCCAAGAGTCGAACGCC GAG	
oDA507	CATCAAAACAAGCTACACTGACGCTTGACAGCTACACGTTCTGCAAGC GTAACAATGTTAAAGTAGACCGAACAG	
oDA508	CTTTAACATTGTTACGCTTGCAGAACGTGTAGCTGTCAAGCGTCAGTG TAGCTTG	
oDA509	CTATAGGGAGACCGGCAGATCTGATATCATCGCCGCTGCATGCGGTCT TGACCG	
oPH376	AGCATTCAAAATTCCATTCTACAACGTCGAGAAACGAAACCTCGGGTT TTAGAGCTAGAAATAG	
oPH377	CTATTTCTAGCTCTAAAACTCACGGCCCAAGCTGCGCCTTGACCAGAC GGGATTCG	
oPH378	CGAATCCCGTCTGGTCAAGGCGCAGCTTGGGCCGTGAGTTTTAGAGCT AGAAATAG	
oPH379	CTATTTCTAGCTCTAAAACTGCTCTGTGCCATCGTCTTCTGCATCGACG GGGAATCGAAC	
oPH380	GTTCGATTCCCCGTCGATGCAGAAGACGATGGCACAGAGCAGTTTTAG AGCTAGAAATAG	
oPH138	GGCGTTCGACTCTTGGCAG	
oDA375	GTAGCAGTCTGTCAGCATTCAAAATTCCATTCTACAACGGCCGATACT TGTCGCCGTTCGTTTTAGAGCTAGAAATAG	
oDA389	TGGTGCACGTCTCCTTGAACCTTGACCAGACGGGATTCG	
oDA390	GGTTCAAGGAGACGTGCACCAGTTTTAGAGCTAGAAATAGCAAG	
oDA556	CTCGTTTGTCGCGGGTTCGAATCCCGTCTGGTCAAGGTTCAAGGAGAC GTGCACCAGTTTTAG	
oDA557	GCATTTCGCCTTCTCGGCGTTCGACTCTTGGCAGGTAC	

oNL240	CTCGAGTTTTTCAGCAAGATAATATTATGGTCACGAGCGTAATC	
oNL241	TTGTCACGCCATGGTGGCCATCTAGGCCCGTTGATGTCGAGAGTCC	
oNL242	GCGGCCGCATTAATAGGCCTGAGTGGCCCGTCGTTGATGATGACTC	
oNL243	AGGAGATCTTCTAGAAAGATAATATTCGGCTGAATACAAGTCAC	
oDA367	GATGGCTCGAGTTTTTCAGCAAGATAATATTCCAAGCACAAAGTCCAC TGC	SspI
oDA368	CGTGATTCTTTACAGCAGAGCTCGTGTTCTCTGCTTTATTTTCTCGATA AAGTTG	
oDA369	CAACTTTATCGAGAAAATAAAGCAGAGAACACGAGCTCTGCTGTAAA GAATCACG	
oDA370	GTAGGAGATCTTCTAGAAAGATAATATTCAACATTTCTCTCGTTGTCG G	SspI
oDA371	GATGGCTCGAGTTTTTCAGCAAGATAATATTGGCCTTTCCAGGACTCA AACATAATTTC	SspI
oDA372	CTTTACCAAGGCTTTTTTGCGCCTTCGTGTGGCGATCGTACGTCC	
oDA373	GGACGTACGATCGCCACACGAAGGCGCAAAAAAGCCTTGGTAAAG	
oDA374	GTAGGAGATCTTCTAGAAAGATAATATTGCCCACACCGCTCTGCTAAT TTTC	SspI
oDA443	GAACTCGAGCAGCTGAAGCTTGCATGCTCACGGCTTGGTGTAGATG	SphI
oDA444	GTTATCCTCCTCGCCCTTGCTCACCATGCTAGCGGTGCACTTGTTGGCG TG	BmtI
HvPR1_RT_fw	GGACTACGACTACGGCTCCA	
HvPR1_RT_rv	GGCTCGTAGTTGCAGGTGAT	
HvPR10_RT_fw	GGAGGGCGACAAGGTAAGTG	
HvPR10_RT_rv	CGTCCAGCCTCTCGTACTC	
HvWRKY22_RT-fw	GGAGAAACGGAAGCATCAG	
HvWRKY22_RT-rv	GCTTGGCTCCAAGAATGTC	
HvGAPDH-RT_fw	GGCCGCAAGCTTTAACATC	
HvGAPDH-RT_rv	GGAACCCGGAAAGACATAC	
oMM780	CATATGAGCGTAATCTGGTACG	

Supplementary Information References:

References 1-72 can be found in the main part of the manuscript.

73 Lo Presti, L. *et al.* An assay for entry of secreted fungal effectors into plant cells. *New Phytol.* **213**, 956-964 (2017).

74 Sarkari, P. *et al.* Improved expression of single-chain antibodies in *Ustilago maydis*. *J. Biotechnol.* **191**, 165-175 (2014).

75 Banuett, F. & Herskowitz, I. Different a alleles of *Ustilago maydis* are necessary for maintenance of filamentous growth but not for meiosis. *Proc. Natl Acad. Sci.* **86**, 5878-5882 (1989).

76 Linning, R. *et al.* Marker-based cloning of the region containing the *UhAvr1* avirulence gene from the basidiomycete barley pathogen *Ustilago hordei*. *Genetics* **166**, 99-111 (2004).

77 Aichinger, C. *et al.* Identification of plant-regulated genes in *Ustilago maydis* by enhancer-trapping mutagenesis. *Mol. Genet. Genomics* **270**, 303-314 (2003).

78 Khrunyk, Y., Münch, K., Schipper, K., Lupas, A. N. & Kahmann, R. The use of FLP-mediated recombination for the functional analysis of an effector gene family in the biotrophic smut fungus *Ustilago maydis*. *New Phytol.* **187**, 957-968 (2010).

79 Christianson, T. W., Sikorski, R. S., Dante, M., Shero, J. H. & Hieter, P. Multifunctional yeast high-copy-number shuttle vectors. *Gene* **110**, 119-122 (1992).

80 Schuster, M., Schweizer, G. & Kahmann, R. Comparative analyses of secreted proteins in plant pathogenic smut fungi and related basidiomycetes. *Fungal Genet. Biol.* **112**, 21-30 (2018).

81 Baumann, S., Pohlmann, T., Jungbluth, M., Brachmann, A. & Feldbrügge, M. Kinesin-3 and dynein mediate microtubule-dependent co-transport of mRNPs and endosomes. *Journal of Cell Science* **125**, 2740-2752 (2012).
