# Supplementary material for: A cell surface-exposed protein complex with an essential virulence function in Ustilago maydis
Source: Nat Microbiol. 2021 May 3;6(6):722–30. doi: 10.1038/s41564-021-00896-x (PMC8159752; doi:10.1038/s41564-021-00896-x)
Supplement: Supplementary file 2 — Reporting Summary [file 41564_2021_896_MOESM2_ESM.pdf]

nature research

Corresponding author(s): Regine Kahmann

Last updated by author(s): 13/3/21

Reporting Summary

Nature Research wishes to improve the reproducibility of the work that we publish. This form provides structure for consistency and transparency in reporting. For further information on Nature Research policies, see our Editorial Policies and the Editorial Policy Checklist.

Statistics

For all statistical analyses, confirm that the following items are present in the figure legend, table legend, main text, or Methods section.

n/a	Confirmed	
☐	☒	The exact sample size (*n*) for each experimental group/condition, given as a discrete number and unit of measurement
☐	☒	A statement on whether measurements were taken from distinct samples or whether the same sample was measured repeatedly
☐	☒	The statistical test(s) used AND whether they are one- or two-sided<br>*Only common tests should be described solely by name; describe more complex techniques in the Methods section.*
☒	☐	A description of all covariates tested
☒	☐	A description of any assumptions or corrections, such as tests of normality and adjustment for multiple comparisons
☐	☒	A full description of the statistical parameters including central tendency (e.g. means) or other basic estimates (e.g. regression coefficient) AND variation (e.g. standard deviation) or associated estimates of uncertainty (e.g. confidence intervals)
☒	☐	For null hypothesis testing, the test statistic (e.g. *F*, *t*, *r*) with confidence intervals, effect sizes, degrees of freedom and *P* value noted<br>*Give P values as exact values whenever suitable.*
☒	☐	For Bayesian analysis, information on the choice of priors and Markov chain Monte Carlo settings
☒	☐	For hierarchical and complex designs, identification of the appropriate level for tests and full reporting of outcomes
☒	☐	Estimates of effect sizes (e.g. Cohen's *d*, Pearson's *r*), indicating how they were calculated

Our web collection on statistics for biologists contains articles on many of the points above.

Software and code

Policy information about availability of computer code

Data collection

qPCR data was collected by CFX manager software (Biorad). Confocal microscopy was performed using a Leica TCS SP8x/WLL (White Light Laser) confocal laser-scanning microscope (Leica, Wetzlar, Germany). For image deconvolution the HyVolution software package (Leica and Scientific Volume Imaging B.V., Hilversum, Netherlands) was used.

Data analysis

Exel 2013 (Microsoft), Mascot 2.5 (Matrix Science), Scaffold (v4.6.2, Proteome Software), Cytoscape (v3.3.0), MaxQuant (Version 1.6.3.4), Perseus (v1.5.2.6), CLC genomics workbench (Qiagen, version 9.5.3), Augustus gene prediction program (Version 2019 (http://bioinf.uni-greifswald.de/augustus/)), Clustal Omega provided by the Swiss Institute of Bioinformatics (Version 2019 (https://www.ebi.ac.uk/Tools/msa/clustalo/)), BOXSHADE (Version 3.21 provided by the EMBnet (https://embnet.vital-it.ch/software/BOX_form.html)), SignalP v. 5 (http://www.cbs.dtu.dk/services/SignalP/), TMHMM v. 2.0. (http://www.cbs.dtu.dk/services/TMHMM/), GraphPad Software, Inc. Prism 8.1 (https://www.graphpad.com), Leica Application Suite X, version 3.1.5.

For manuscripts utilizing custom algorithms or software that are central to the research but not yet described in published literature, software must be made available to editors and reviewers. We strongly encourage code deposition in a community repository (e.g. GitHub). See the Nature Research guidelines for submitting code & software for further information.

Data

Policy information about availability of data

All manuscripts must include a data availability statement. This statement should provide the following information, where applicable:

- Accession codes, unique identifiers, or web links for publicly available datasets
- A list of figures that have associated raw data
- A description of any restrictions on data availability

U. maydis genes and encoding protein sequences are available at NCBI under the following accession numbers: U. maydis stp1 (UMAG_02475), XP_011388756.1; U.

maydis stp2 (UMAG_10067), XP_011388794.1; U. maydis stp3 (UMAG_00715), XP_011386505.1; U. maydis stp4 (UMAG_12197), XP_011389576.1; U. maydis pep1 (UMAG_01987), XP_011387901.1; U. maydis stp5 (UMAG_04342), XP_011391052.1; U. maydis stp6 (UMAG_01695), XP_011387671.1

Protein and DNA sequences of stp orthologues from 11 sequenced smuts were derived from the following data sources: U. esculenta: DDBJ/EMBL/GenBank accession number JTLW00000000, version JTLW0100000059, U. trichophora RK089: DDBJ/ENA/GenBank accession number LVYE00000000, version LVYE0100000060, U. tritici: DDBJ/ENA/GenBank accession number NSHH0000000, version NSHH0100000061, Sporisorium reilianum f.sp. zeae SRZ2: European Molecular Biology Laboratory database accession numbers FQ311430 to FQ31147462, Sporisorium reilianum f.sp. reilianum SRS1_H2-8: European Nucleotide Archive (ENA) at http://www.ebi.ac.uk/ena/data/view/LT795054-LT79507663,64, Sporisorium scitamineum Sscl8: ENA at http://www.ebi.ac.uk/ena/data/view/ LK056649-LK05669565. U. maydis 521: https://mycocosm.jgi.doe.gov/Ustma2_2/Ustma2_2.home.html18, U. hordei Uhor01: DDBJ/ENA/GenBank accession number NSDP00000000 version NSDP0100000061, U. hordei Uh4875-4: ENA at http://www.ebi.ac.uk/ena/data/view/ accession numbers CAGI01000001 to CAGI0100071366, U. bromivora UB2112: ENA at http://www.ebi.ac.uk/ena/data/view/PRJEB7751 accession number PRJEB775167, M. pennsylvanicum Mp4: ENA at accession number PRJEB4565, accession IDs HG529494 to HG52992868. M. pennsylvanicum Mp4 was resequenced by PacBio and gene information from this project was included (R. K., unpublished).
The data that support the findings of this study are available from the corresponding author upon request.

Field-specific reporting

Please select the one below that is the best fit for your research. If you are not sure, read the appropriate sections before making your selection.

☒ Life sciences ☐ Behavioural & social sciences ☐ Ecological, evolutionary & environmental sciences

For a reference copy of the document with all sections, see nature.com/documents/nr-reporting-summary-flat.pdf

Life sciences study design

All studies must disclose on these points even when the disclosure is negative.

Sample size	Plant infections with Ustilago maydis strains to be tested were done in 3 biological replicates with about 30 to 40 plants for each replicate. Individual plants were scored for disease severity using a published scoring scheme. Only infections done at the same time are grouped together.
Data exclusions	No data were excluded from the analysis.
Replication	In cases where representative pictures are shown the experiments were repeated at least twice with similar results.
Randomization	No randomization was necessary.
Blinding	Blinding was not relevant for our study.

Reporting for specific materials, systems and methods

We require information from authors about some types of materials, experimental systems and methods used in many studies. Here, indicate whether each material, system or method listed is relevant to your study. If you are not sure if a list item applies to your research, read the appropriate section before selecting a response.

Materials & experimental systems

n/a	Involved in the study	
☐	☒	Antibodies
☒	☐	Eukaryotic cell lines
☒	☐	Palaeontology and archaeology
☒	☐	Animals and other organisms
☒	☐	Human research participants
☒	☐	Clinical data
☒	☐	Dual use research of concern

Methods

n/a	Involved in the study	
☒	☐	ChIP-seq
☒	☐	Flow cytometry
☒	☐	MRI-based neuroimaging

Antibodies

Antibodies used	Rabbit anti-HA primary antibody from rabbit for HA-protein fusion detection (1:10.000 dilution, Sigma-Aldrich, H6908, polyclonal, Lot#015M4868V),<br><br>Mouse anti-HA monoclonal primary antibody from mouse for HA-protein fusion detection (1:5.000 dilution, Sigma-Aldrich, H9658, clone HA-7, Lot#127M4869V)<br><br>Mouse anti-myc monoclonal primary antibody from mouse for myc-protein fusion detection (1:10.000 dilution, Sigma-Aldrich, M4439, Clone9E10, Lot#087M4765V)

Anti-a-tubulin monoclonal primary antibody from mouse (1:2.000 dilution, Calbiochem®, CP06, Clone DM1A, Lot#2681308)

Anti-rabbit lgG secondary antibody (1:10.000 dilution, Cell Signaling Technology #7074)
Anti-mouse lgG secondary antibody (1:10.000 dilution Cell Signaling Technology #7076)

For Immunolocalization:
Mouse anti-HA monoclonal primary antibody from mouse for HA-protein fusion detection (1 : 1500 dilution, Sigma-Aldrich, H9658, clone HA-7, Lot#089M4796V)
Alexa Fluor 488 goat anti-mouse lgG (H+L) (1 : 1500 dilution, Life Technologies Lot#1890503)

For Immuno-TEM:
Primary: 1:60 dilution, Thermofisher Catalog #26183 Invitrogen HA Tag Monoclonal Antibody (2-2.2.14),
Secondary: 1:50 dilution, Electron Microscopy Sciences #25121 Aurion Goat anti-mouse IgG Ultra Small Lot# GG-71025/2

Validation

All antibodies directed against epitope-tags were validated by using them on strains not expressing the epitope -tagged protein either by immuno-EM or by western. Respective controls are included and where antibodies recognize non-epitope-tagged proteins on western blots these are indicated.
All commercial antibodies are validated by the manufacturer and the statements are available on the manufacturer's website.
