# Supplementary figures and images for: A cell surface-exposed protein complex with an essential virulence function in Ustilago maydis
Source: Nat Microbiol. 2021 May 3;6(6):722–30. doi: 10.1038/s41564-021-00896-x (PMC8159752; doi:10.1038/s41564-021-00896-x)

Source Data Extended Data Fig. 1

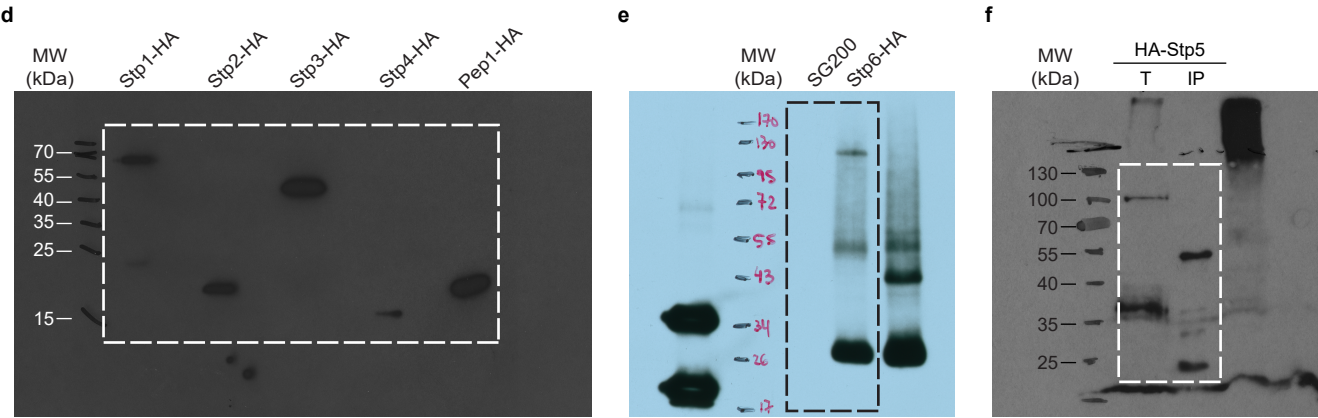

Supplement: Source Data Extended Data Fig. 1 — Unprocessed western blots. [file 41564_2021_896_MOESM6_ESM.pdf]

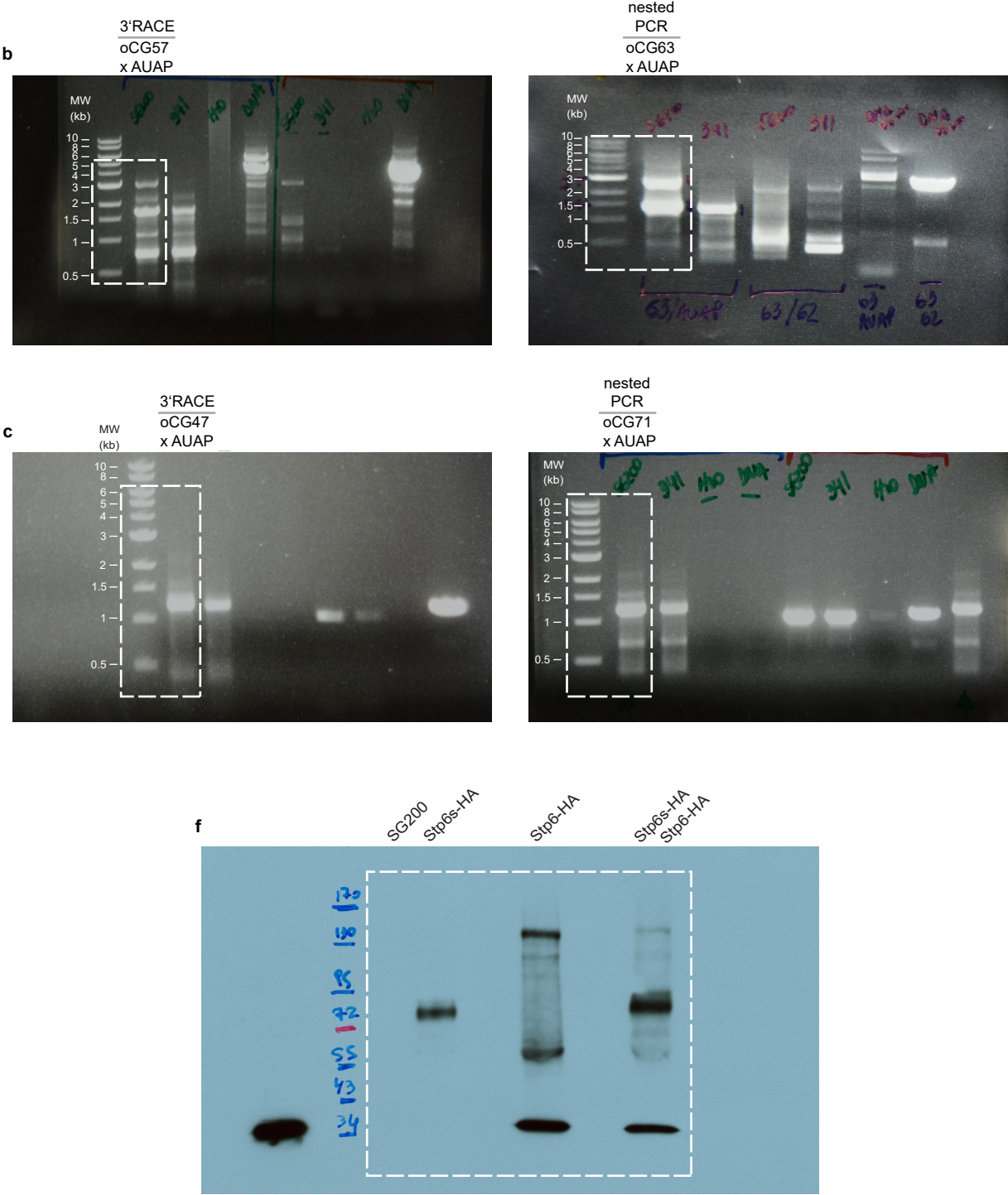

Supplement: Source Data Extended Data Fig. 2 — Unprocessed western blots and gels. [file 41564_2021_896_MOESM7_ESM.pdf]

Source Data Extended Data Fig. 3

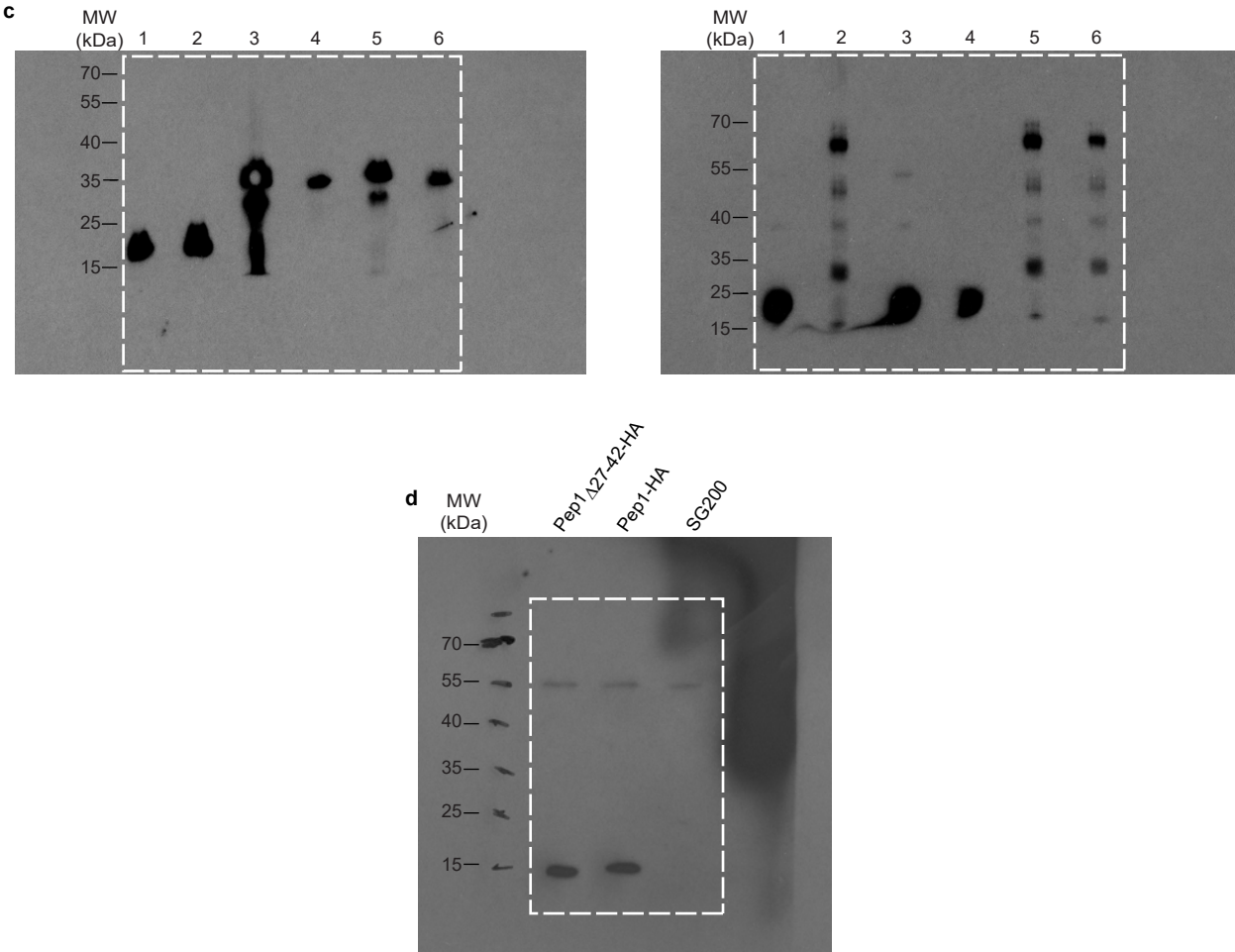

Supplement: Source Data Extended Data Fig. 3 — Unprocessed western blots. [file 41564_2021_896_MOESM8_ESM.pdf]

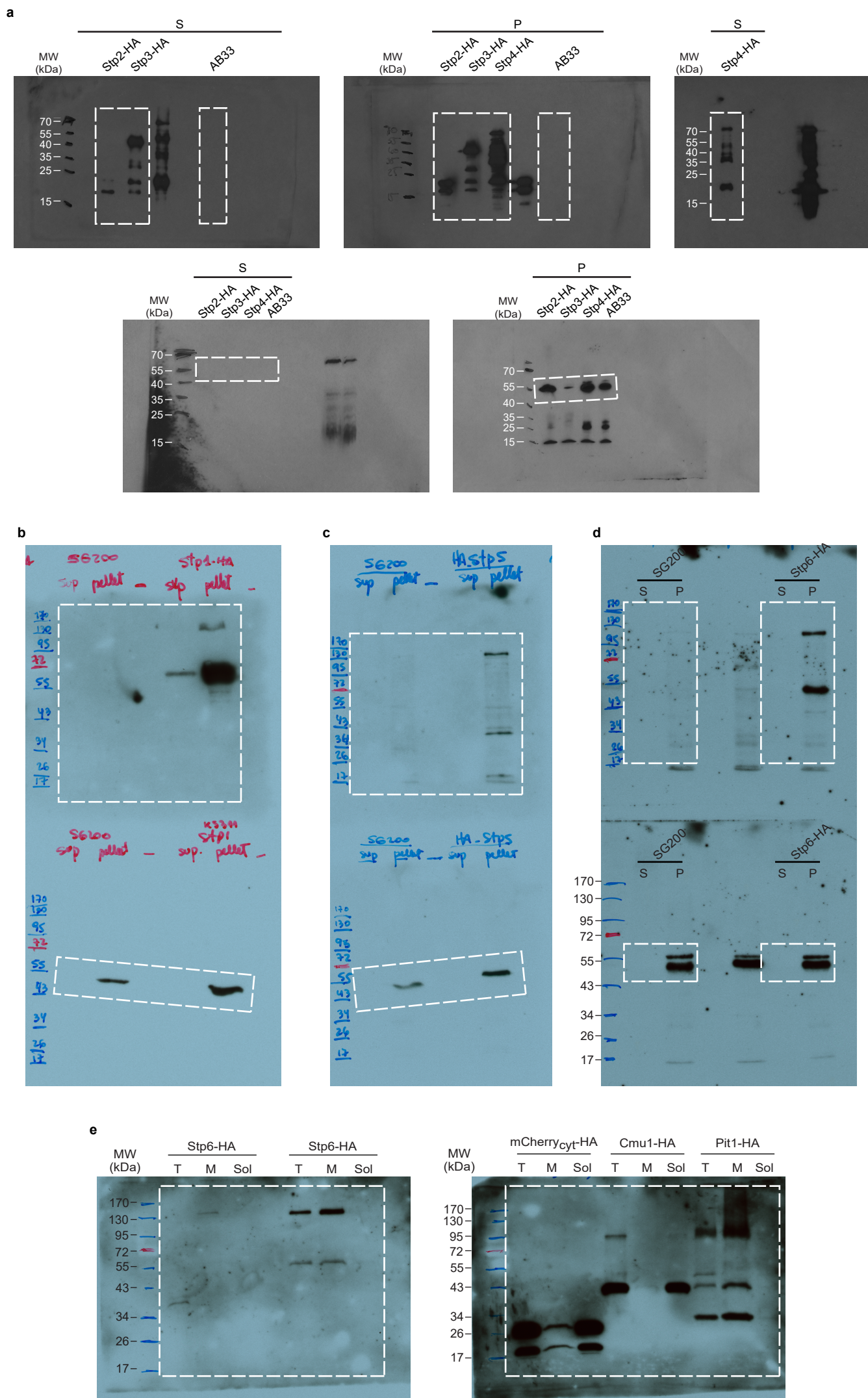

Supplement: Source Data Extended Data Fig. 4 — Unprocessed western blots. [file 41564_2021_896_MOESM9_ESM.pdf]

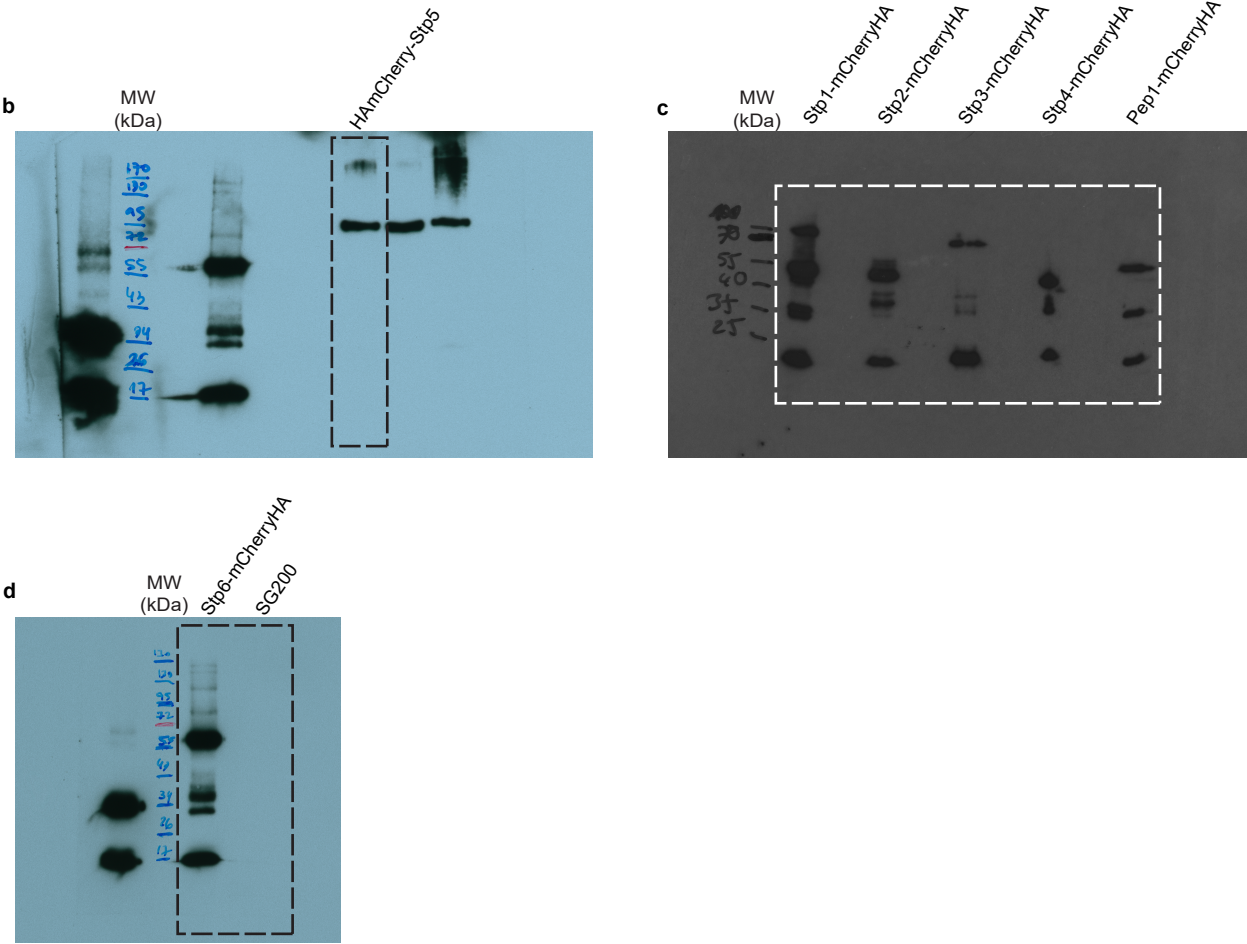

Supplement: Source Data Extended Data Fig. 5 — Unprocessed western blots. [file 41564_2021_896_MOESM10_ESM.pdf]
